# Selectivity and stability of N-terminal targeting protein modification chemistries†

**DOI:** 10.1039/d2cb00203e

**Published:** 2022-11-17

**Authors:** Lydia J. Barber, Nicholas D. J. Yates, Martin A. Fascione, Alison Parkin, Glyn R. Hemsworth, Paul G. Genever, Christopher D. Spicer

**Affiliations:** a Department of Chemistry, University of York Heslington YO10 5DD UK chris.spicer@york.ac.uk; b York Biomedical Research Institute, University of York Heslington YO10 5DD UK; c Department of Biology, University of York Heslington YO10 5DD UK; d Astbury Centre for Structural Molecular Biology and School of Molecular and Cellular Biology, Faculty of Biological Sciences, University of Leeds Leeds LS2 9JT UK

## Abstract

Protein N-termini provide uniquely reactive motifs for single site protein modification. Though a number of reactions have been developed to target this site, the selectivity, generality, and stability of the conjugates formed has not been studied. We have therefore undertaken a comprehensive comparative study of the most promising methods for N-terminal protein modification, and find that there is no ‘one size fits all’ approach, necessitating reagent screening for a particular protein or application. Moreover, we observed limited stability in all cases, leading to a need for continued innovation and development in the bioconjugation field.

## Abstract 2

Protein N-termini provide uniquely reactive motifs for single site protein modification. A comprehensive comparative study of the most promising modification reagents is performed, finding that there is no ‘one size fits all’ approach.

## Graphical abstract

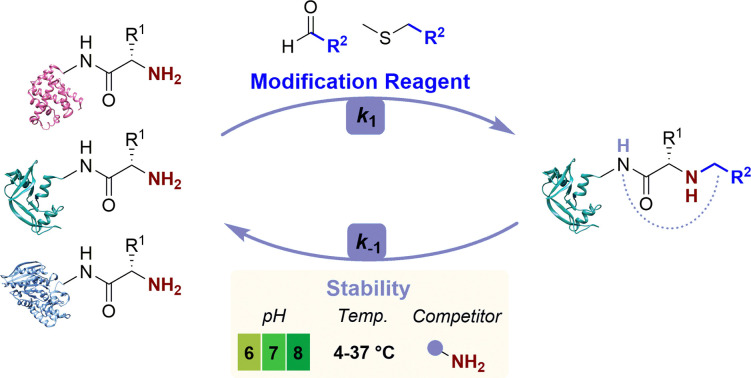

Chemically-modified proteins are at the forefront of innovation in biomedicine and biotechnology. By expanding the structure and function of proteins beyond what can be achieved in nature, site-selective modification chemistries are driving applications in fields as diverse as drug delivery, biosensing, and waste remediation.^1–3^ However, achieving site-selectivity remains a complex challenge, requiring exquisite regio- and chemo-selectivity under restrictive conditions.^4^ Reactions targeting naturally occurring amino acids, most commonly cysteine, are reliant on the presence of a singly reactive residue on the protein surface, in an accessible and suitable position for modification that will not adversely affect protein activity. In contrast, powerful technologies that allow the site-specific installation of unnatural amino acids bearing bio-orthogonal handles for modification typically require genetic and protein engineering processes that may not always be suitable or accessible.^5^

In this context, the development of chemical reactions that target the N-termini of proteins is particularly attractive for site-selective modification. Although up to 80-90% of the eukaryotic proteome is N-terminally acetylated,^6^ in most bacterial and secreted proteins (*e.g.* antibodies) the N-terminus is free, and chemically and sterically accessible.^7^ Moreover, N-termini are commonly positioned away from active or binding sites, providing a convenient handle to achieve labelling without a cost to bioactivity. In recent years, there has therefore been a surge in interest in the development of N-terminal targeting protein chemistries.

Despite these developments, most reported strategies for N-terminal modification lack universal sequence compatibility or show poor reactivity, selectivity, or conjugate stability. Furthermore, differences in target proteins and experimental set-ups make comparisons between different approaches challenging and the impact of these drawbacks on protein modification are therefore not always clear. In this paper, we therefore carried out a detailed comparative study of the most promising strategies for generalised N-terminal chemical modification, with the aim of elucidating more mechanistic and kinetic insight into the factors governing selectivity, reactivity, and stability. By doing so, we aim to provide valuable insight into the appropriate N-terminal targeting chemistry for a given protein/application, and a platform for researchers to develop new modification strategies which address current limitations in the field.

## Results and discussion

### Study design

As the only α-amido amine present in native proteins, the N-terminus provides a chemically unique target for chemical modification: (i) The lower p*K*_a_ of the N-terminal ammonium group (∼6.0–8.0), relative to the *ε*-ammonium of lysine (∼10.5), marks it as the most nucleophilic amine at physiological pH, allowing selective modification with electrophiles under careful pH control;^7^ and (ii) the α-amide itself can participate in conjugation, providing a unique chemical motif.^8^ With the goal of identifying generalisable strategies for N-terminal protein modification, we chose to study only reactions that possess near-universal sequence compatibility. We therefore excluded reactions that take place exclusively at N-terminal cysteines,^9,10^ serines,^11^ glycines,^12^ or biomimetic sequences,^13^ which though powerful techniques are not as generally translatable. Similarly, we omitted reactions which modify N-termini preferentially but which also readily modify lysine when >1 equiv. reagent is used,^14^ as well as for which organic co-solvents are necessary.^15^

**Fig. 1:**
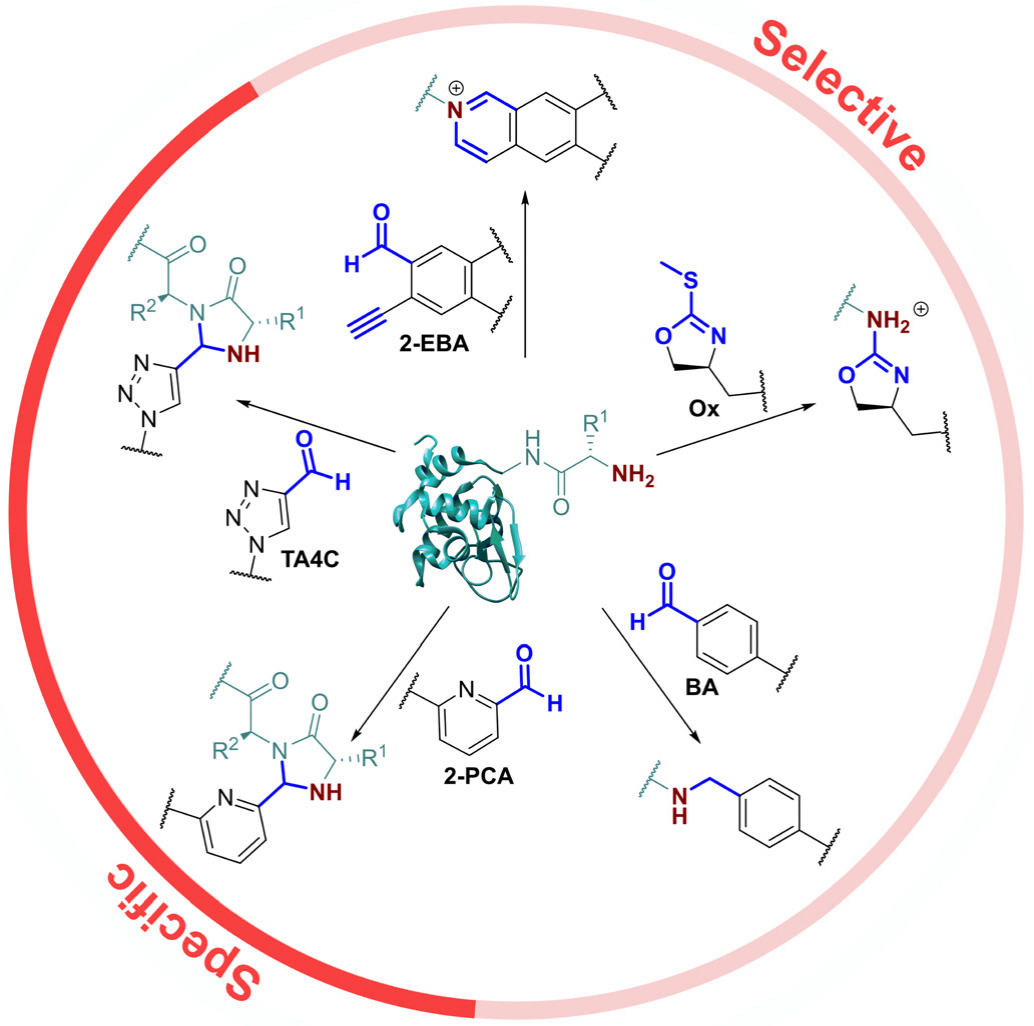
Previously reported universal strategies for targeting protein N-termini, which can be split into selective and specific methods.

In this context, the pioneering work of the Francis group on 2-pyridinecarboxaldehydes (2-PCAs) stands out as one of the earliest examples of a reagent that can target all protein N-terminal residues (other than with proline at the second position).^8^ Since then, a number of general conjugation strategies have been reported. We classify these into two categories (Fig. 1): (i) *Selective* strategies, which target the N-terminus through precise control of conditions such as pH and stoichiometry, to minimise side reactions at lysine. These strategies typically rely on the p*K*_a_ of the N-terminus; and (ii) *Specific* strategies which can only take place at the N-terminus, typically due to contributions from the α-amide.

The most classical selective reactions rely on benzaldehyde (BA) handles that can reversibly form imines at protein N-termini, and be trapped *via* reductive amination.^16^ More recently, 2-ethynylbenzaldehydes (2-EBA) have been shown to similarly target N-termini *via* the formation of isoquinolinium salts following initial imine formation.^17^ Selective modification has also been reported using oxazolines (Ox), *via* azolation.^18^

In contrast, the 2-PCAs developed by the Francis group undergo cyclisation *via* nucleophilic attack of the α-amide at the intermediate imine formed *via* initial condensation at the N-terminus.^8^ The resulting imidazolidinone can only form at the N-terminus, making these reagents specific. Imine formation at the ε-amine of lysine is also possible, but hydrolysis back to the aldehyde is favoured in an aqueous environment. A similar mode of reactivity has been reported for triazolecarbaldehyde (TA4C) reagents introduced recently by Onoda *et al*.^19^

**Fig. 2:**
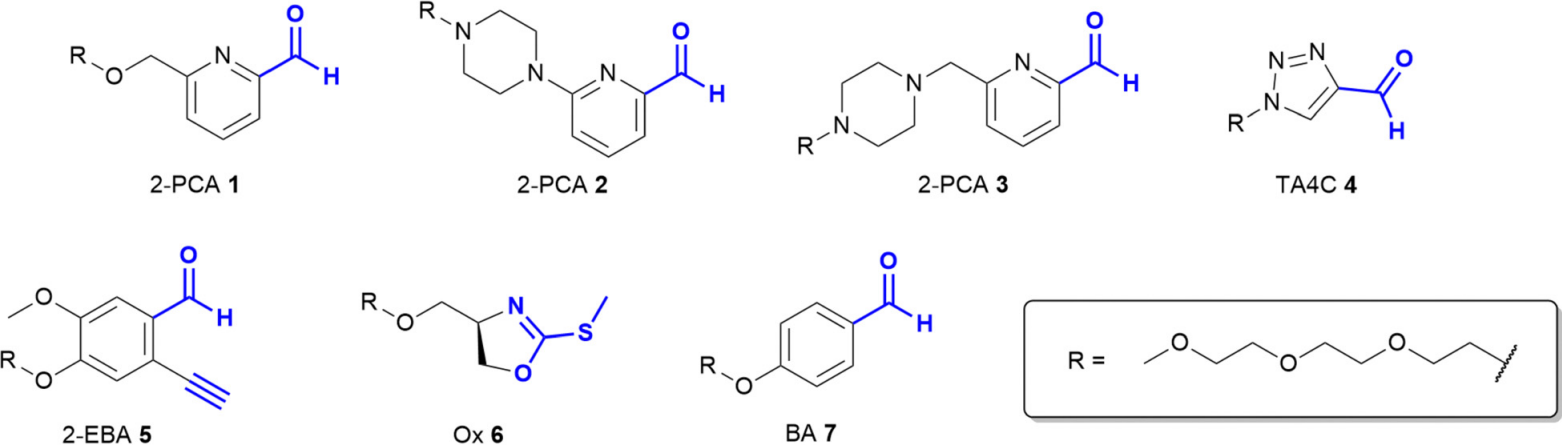
Structures of reagents 1–7, for N-terminal protein modification.

Based on these prior reports, we synthesised a panel of reagents, 1–7 which represented the key reagent classes discussed above (Fig. 2, see ESI† Scheme S1). Reagents 1–3 are based on the 2-PCAs reported by the Francis group, with 6-amino-2-PCAs (2) having been reported to increase reactivity relative to 6-methylamino derivatives (3).^20^ We also considered the synthetic accessibility of 6-methyloxy analogue 1, which can be synthesised directly from the corresponding alcohol *via* an etherification reaction. TA4C 4, 2-EBA 5, Ox 6, and BA 7 were similarly synthesised based on the most reactive structures reported in the relevant papers. A triethylene glycol monomethyl ether side chain was incorporated into each reagent to ensure water-solubility and eliminate any differences in reactivity that might arise from the side chain.

### Protein modification

The reactivity of 1–7 was initially validated by undertaking modification of either RNase A or myoglobin under identical conditions to those previously reported in the literature for each reagent (see ESI† Table S1 and Fig S5).^8,15,17–20^ Reactions were then analysed prior to removal of the excess reagent *via* intact protein liquid chromatography-mass spectrometry (LC-MS). Conversions observed for 2-PCAs 1–3, TA4C 4, and Ox 6 were comparable to the literature, however 2-EBA 5 and BA 7 gave lower conversions than reported. Deng *et al.* previously found that 2-EBA conjugation efficiency and selectivity varied greatly with protein target, reagent structure, number of equivalents, and reaction pH, and so differences resulting from subtle changes to conditions are not unexpected.^17^ For 7, possible negative contributions from aldehyde oxidation or from the instability of the intermediate imine, known complications of the use of BAs for protein modification, may be the source of our discrepancies.

With protein modification validated, we next made a direct comparison between the reactivity of 1–7. A generalised procedure (50 μM protein, 100 equiv. reagent, pH 7.5, 37 °C, 23 h) was used to maximise applicability to a wide range of protein substrates. These conditions are broadly similar to those used in the literature for N-terminal protein modification, with two notable differences: (i) For Ox 6, protein concentrations of 3 mM were used in the initial report by Tang *et al.*,^18^ but such concentrations are not generalisable due to the propensity of many proteins to undergo aggregation. The use of 50 μM protein was therefore chosen as a more relevant concentration within this study; and (ii) the reactivity of BA 7 is highest at pH 6.1, but again this leads to a loss of generalisability.^15^ All reactions were therefore performed at pH 7.5, with sodium cyanoborohydride required in all reactions involving 7 to reduce the intermediate imine.

Our representative reaction conditions were applied to a panel of model proteins (Table 1). RNase A (N-terminal Lys, 10 total Lys residues), equine myoglobin (N-terminal Gly, 20 Lys), and clostripain light chain (LC, N-terminal Asn, 14 Lys) were chosen due to their ease of MS detection. Our lack of protein purification prior to analysis during this study is notable, allowing us to observe varying levels of multi-site protein modification, due to either transient or stable adducts with other amino acids. Our results showed that all reagents exhibited some degree of off-target reactivity, highlighting the need for extensive protein purification to remove excess reagent, as discussed later.

**Table 1:** Conversions for the modification of a panel of proteins with reagents 1–7. Selectivity is shown in *italics*, and entries colour coded based on the yield of singly modified protein (green = highest; red = lowest; s = single, d = double, t = triple, q = quadruple, qu = quintuple, se = sextuple, sp = septuple modification). The N-terminal residue of each protein is given in brackets

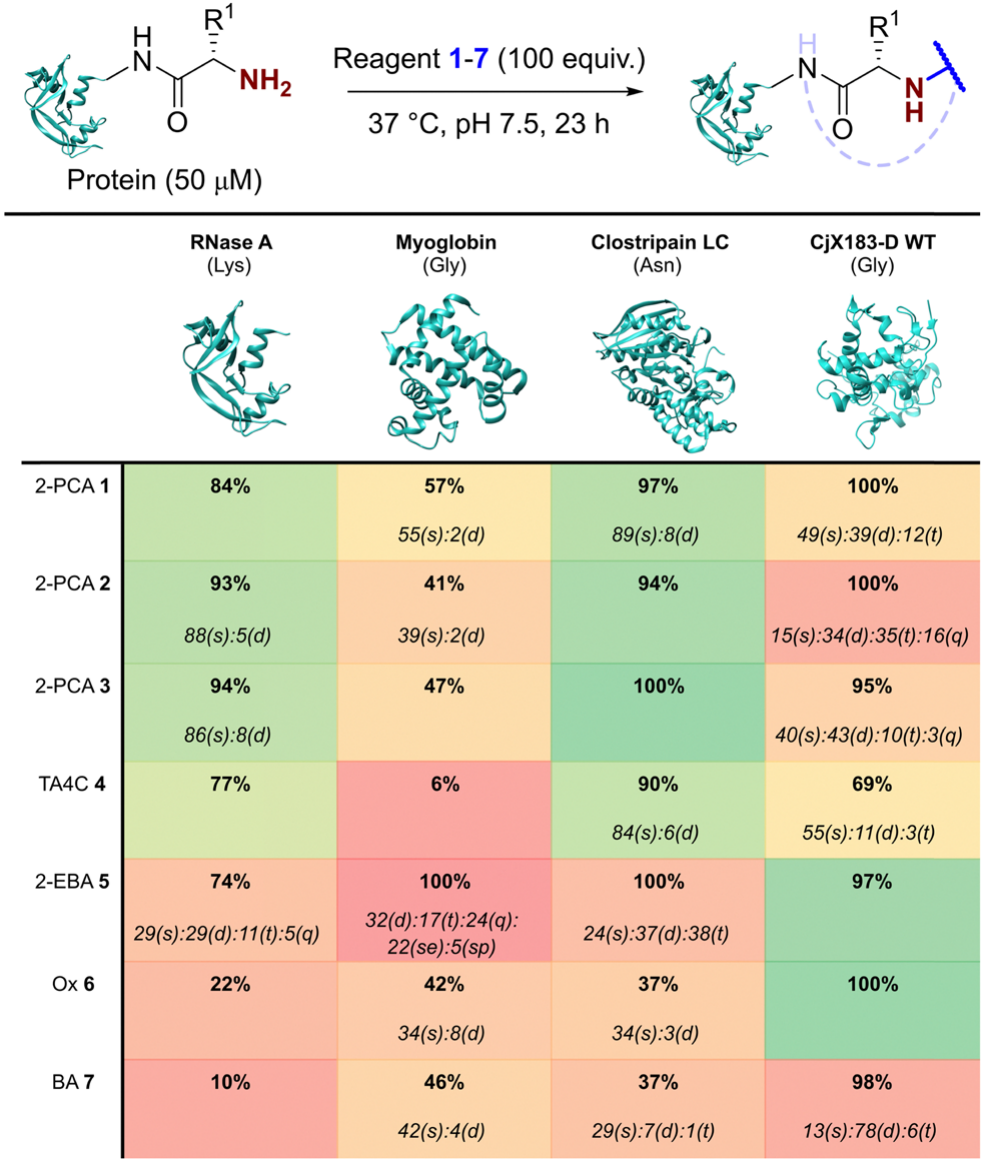

Studying this panel of proteins revealed that each reagent had distinct protein labelling preferences, with no generalisable trends observed whereby a decrease in conjugation efficiency for one reagent was mirrored in a decrease for all others too. In general, 2-PCAs 1–3 showed the highest reactivity, with modifications of RNase A of 84–94% and of clostripain LC of 94–100%. Conjugation efficiency for myoglobin was reduced (41–57%) with little difference in reactivity between the three constructs being studied. Koo *et al.* previously reported an increase in reactivity when the piperazine was attached directly to the PCA ring *via* an Ar–NR_2_ bond, as in reagent 2, rather than the Ar–CH_2_NR_2_ seen in reagent 3.^20^ However, these differences were not observed here with 1–3 behaving similarly. It is possible that the differences observed by Koo *et al.* may have been borne out at lower reagent concentrations or *via* a kinetic analysis. The reactivity of 1 indicated that the piperazine motif did not affect conjugation efficiency, opening up alternative structures for functional 2-PCAs.

In general, TA4C 4 showed similar but lower reactivity to the 2-PCA reagents, with conjugation to myoglobin (6%) notably low. In contrast, 2-EBA 5, Ox 6, and BA 7 all exhibited higher reactivity with myoglobin (100, 42, and 46% modification respectively) than for RNase A (74, 22, and 10% modification). Though this may indicate that labelling preferences are conserved when reagents are grouped as either N-terminal specific or N-terminal selective, a larger study would be required to confirm this.

During our studies, it became clear that the N-terminal selectivity of 2-EBA 5 was poor in our hands. At 100 equiv. reagent, high levels of double, triple, and even quadruple modification were observed. At 200 equiv. this off-target reactivity became so pronounced that protein signals could not be deconvoluted, presumably due to a mix of heterogeneously modified proteins bearing different numbers and sites of modification (See ESI† Table S3 and Fig. S7).^21^ Single site modification could be observed by using just 10 equiv. of 5, but at the cost of conversion. Moreover, given the poor selectivity observed at higher reagent loadings, it is plausible that for proteins bearing a single modification conjugation had taken place at another site.

Site-selectivity was generally lower for the N-terminal selective reagents, with 6 and 7 also exhibiting various levels of off-target modification. This is not surprising, given the subtle differences in basicity and nucleophilicity of the α- and ε-amines of the N-terminus and lysine side chain respectively, particularly given the importance of the surrounding environment in dictating protonation state and nucleophilicity. However, the lack of purification prior to analysis also allowed us to observe low levels of off-target reactivity for N-terminal specific reagents 1–4. We hypothesised that these modifications were predominantly a result of transient modifications at lysine, which would be expected to hydrolyse over time upon removal of excess reagent.

**Fig. 3:**
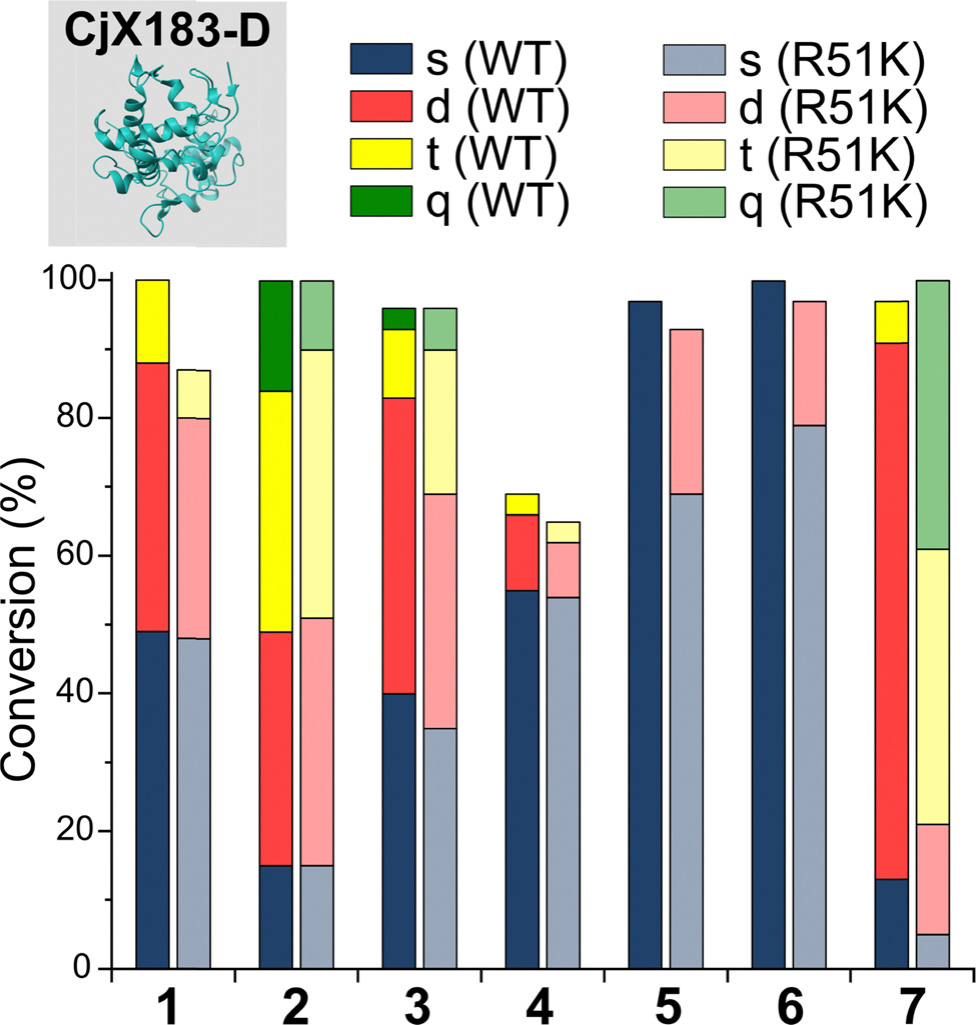
Conversions for the modification of CjX183-D WT (0 Lys) and CjX183-D R51K (1 Lys) prior to purification.

To probe this hypothesis, we studied the modification of CjX183-D, a naturally occurring cytochrome from *Cellvibrio japonicus* which does not contain any lysine residues.^22^ N-terminal selectivity was compared to the mutant CjX183-D R51K, in which a single lysine had been installed (Fig. 3). This study revealed a number of interesting results. For 2-EBA 5 and Ox 6, exclusive single-site modification was observed for CjX183-D, while significant levels of double modification were observed for CjX183-D R51K, strongly indicating that off-target modification with these reagents takes place primarily at lysine. Selectivity was poor for BA 7, but was worse for CjX183-D R51K, highlighting lysine as a possible, but not exclusive site for off-target modification.

To our surprise, 2-PCAs 1–3 and TA4C 4 had poor selectivity for the N-terminus of CjX183-D, with high levels of double, triple, and even quadruple modification being observed. This suggests that these reagents can undergo off-target modification at amino acids other than lysine, further highlighting the high dependence of modification on protein identity. Interestingly, no change in selectivity was observed for CjX183-D R51K, suggesting that the single lysine is not modified to an appreciable degree. It may be that the unique surface properties of CjX183-D create hyper-reactive residues that would not typically react with 1–4 in other proteins. The multi-site adducts observed had a mass of +18 Da relative to the mass that would result following reaction and dehydration of the 2-PCA/TA4C (as expected for imine formation), indicating a potentially new mode of reactivity. Unfortunately, our attempts to identify the site and nature of modification were unsuccessful, with low surface coverage after digestion complicated by the covalently conjugated haem group of the cytochrome.

### N-terminal selectivity/specificity after purification

**Fig. 4:**
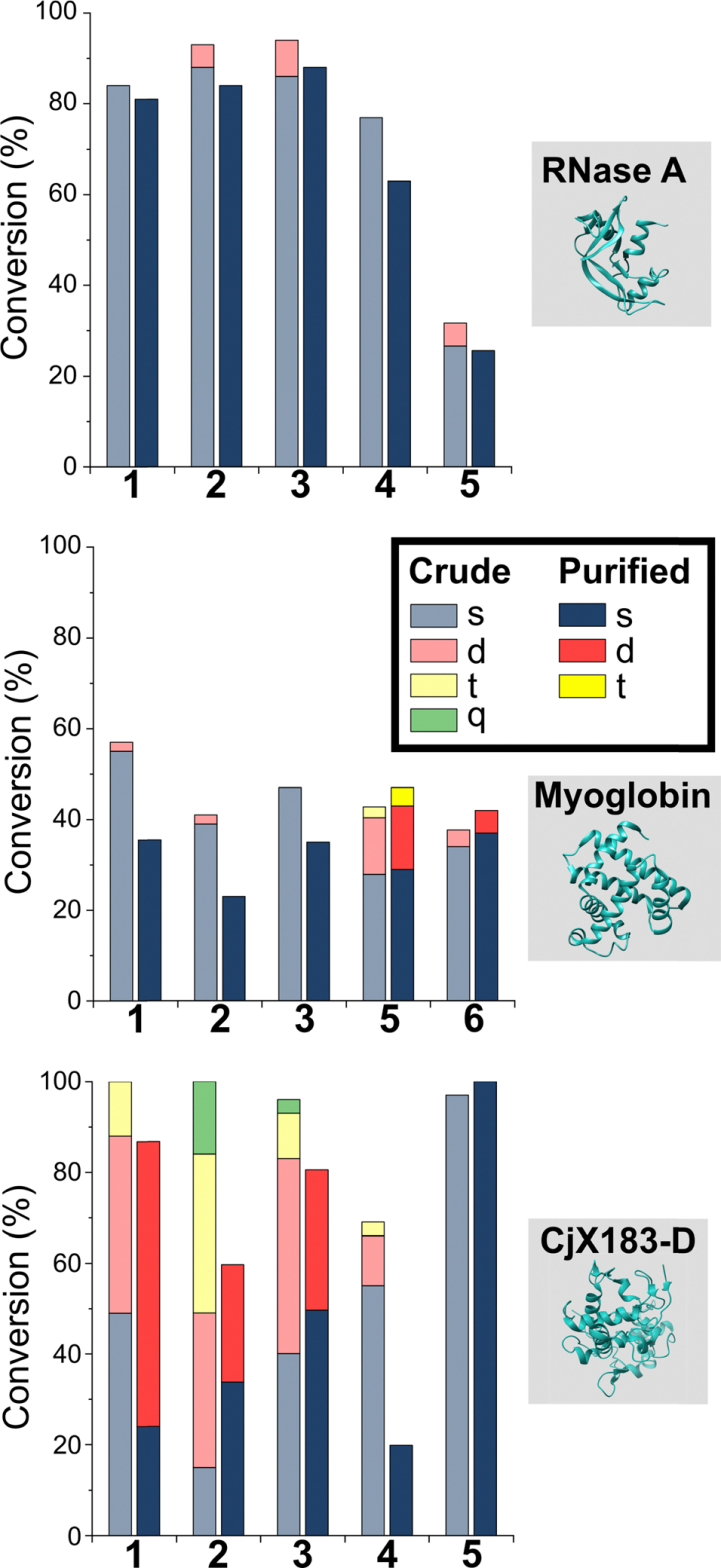
Conversions for the modification of RNase A, myoglobin, and CjX-193-D before (crude) and after dialysis at 4 °C to remove excess reagent and unstable conjugation. Nb, 100 equiv. reagent 1, 3, 4, 6, and 7; 67 equiv. 2 and 25 equiv. 5 were used with myoglobin to allow protein analysis and to prevent over-modification respectively.

Having studied N-terminal modification prior to purification, we next performed an analogous study following removal of excess reagent *via* dialysis at 4 °C (Fig. 4, see below for discussion: though some loss of conjugation took place under these conditions, sufficient levels were maintained to allow qualitative comparisons). In this scenario, transient or unstable modifications, such as hydrolytically sensitive imines, would be cleaved. For RNase A and myoglobin only single site modification was observed following the removal of excess 1–4. For 5 and 6, lower levels of off-target reactivity were found, indicating some level of transient/unstable modification prior to purification. However, significant levels of dual-site modification were still present, presumably at lysine based on our previous experiments.

These results emphasise the benefits of N-terminal *specific vs.* N-terminal *selective* reagents for most proteins. However, high levels of CjX183-D dual modification were still observed for 2-PCAs 1–3 (26–63%). This suggests that the hyper-reactive residues in CjX183-D that lead to this unexpected off-target reactivity produce conjugates of relatively high stability. It is plausible to consider that the singly modified protein that *is* observed may in fact be a mix of N-terminal and off-target conjugates, further reducing confidence in this modification as being targeted predominantly to the N-terminus.

### Conjugate stability

The stability of a modified protein to the conditions in which it is to be used is a critical, but often overlooked aspect of a bioconjugation strategy. MacDonald *et al.* previously reported that 2-PCA conjugates underwent a 20–30% decrease in conjugation over 12 h at 37 °C,^8^ while Deng *et al.* reported 2-EBA derivatives to possess superior stability.^17^ Similarly, Tang *et al.* found Ox-peptide conjugates to be stable at 37 °C for 48 h, though no studies on proteins were performed.^18^ In contrast, the stabilities of TA4C and BA conjugations have not previously been investigated. Moreover, differences in target protein, study design, and conjugation analysis make it difficult to draw fair comparisons between the stability of the reagents used in these separate reports. We therefore studied conjugate stability across a range of conditions that are representative of the expected applications of chemically modified proteins.

**Fig. 5:**
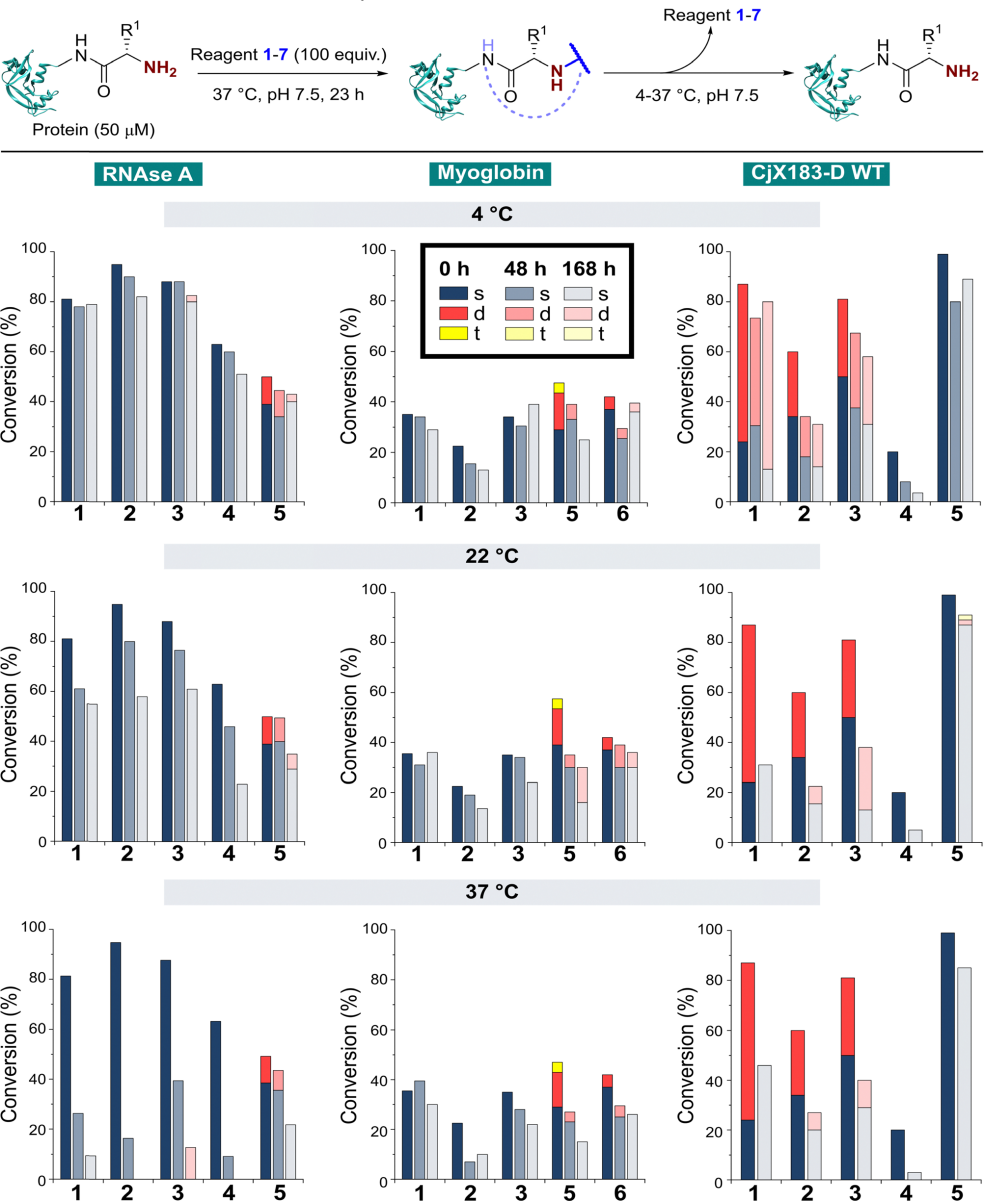
Stability of protein conjugates over time. After initial conjugation at 37 °C for 23 h, proteins were purified by dialysis at 4 °C. Samples were then incubated under the specified conditions and conjugation monitored over time by LC-MS.

RNase A, myoglobin, and CjX183-D were modified with 1–7, as described earlier, and purified by dialysis at 4 °C for 24 h to remove excess reagents. Purified conjugates were then subjected to a range of different conditions (pH 7 at 4 °C, 22 °C, and 37 °C; pH 6 and 8 at 22 °C) under dialysis conditions in sodium phosphate buffer, to remove any unconjugated reagents that were released over time and prevent reattachment. Conjugate stability was then monitored at different time points over 1 week (Fig. 5). In some cases, *e.g.* the modification of myoglobin with TA4C 4, the low initial conversion prior to purification prevented an accurate analysis being undertaken, and these samples were therefore omitted from the study.

With our previous observations that conjugation efficiency for each reagent was strongly protein dependent, it was unsurprising to observe similar effects for the conjugate stability. At 4 °C, all RNase A conjugates were found to be reasonably stable over a 48 h time period (0–9% loss), with 2-PCAs 1–3 and 2-EBA 5 only losing ∼2–12% conjugation over a week. In contrast, modifications of myoglobin were generally found to be less stable at 4 °C, with 2-PCA 2 being notable for the ∼30% loss in conjugation after 48 h. This instability was even more pronounced for CjX183-D, with major drops in conjugation for both 2-PCA 2 (44%) and TA4C 4 (59%).

At 22 °C, RNase A conjugates began to show instability. For N-terminal specific reagents 1–4, between 4–27% of conjugation was lost after 48 h, with 31–64% loss after a week. This instability was even more significant at 37 °C, with between 55–85% loss of conjugation for 1–4 after 48 h, and near complete cleavage after 7 days incubation. 2-EBA 5 adducts were more stable, but still exhibited a 29% loss after 7 days at 22 °C, or 56% at 37 °C. For myoglobin, differences in stability between 4 °C and 22 °C were minimal, but a more significant drop in conjugation upon incubation at 37 °C. Interestingly, among the 2-PCA reagents tested, myoglobin conjugates with 1 were considerably more stable (∼16% loss after 1 week), than those formed with 2 (69% loss after just 48 h). Koo *et al.* previously reported that Ar-NR_2_ based 2-PCAs, such as 2, reacted more rapidly than the corresponding Ar-CH_2_NR_2_ analogues,^20^ but these results suggest a more complex relationship between rate of conjugate formation and subsequent stability, which may differ on a protein by protein basis. Across all of the proteins and conditions tested, we found that pH had little effect on conjugate stability within the range pH 6–8 tested (See ESI† Table S4, Fig. S8).

The stability of off-target modifications relative to the desired N-terminal conjugation has important implications for the preparation of homogenous constructs. Double modifications observed for 2-EBA 5 and Ox 6, discussed above, were found to persist over extended incubation times, albeit with a small reduction over time, particularly for myoglobin. The unexpected dual-site modifications of CjX183-D with 2-PCAs 2–3 were also found to be surprisingly stable. Though some loss of dual conjugation was observed, over 1 week at 22 °C, and even 37 °C, significant levels persisted. This stability was less pronounced for 2-PCA 1, with almost complete conversion to the singly modified protein at both 22 °C and 37 °C. However, given the results discussed above it is not clear if this modification is at the N-terminus, and it is plausible to consider a scenario in which all N-terminal modification is cleaved (given the instability of RNase A and myoglobin modifications) and the undesired off-target modification has been retained. Efforts to probe the unique and intriguing reactivity of this protein are currently underway in our lab.

### Conjugate stability to competing N-termini

In many applications of modified proteins there are likely to be high concentrations of other unlabeled proteins (*e.g.* in blood, protein concentration is in the range 60–80 mg mL^−1^). The stability of an N-terminal conjugate in the presence of an excess of potentially competing N-termini is therefore a critical factor. Koo *et al.* demonstrated that proteins could be stably immobilised on polymeric supports for up to 9 days *via* 2-PCA handles, but in this scenario the local 2-PCA concentration was high (up to 27 mM), driving the equilibrium towards polymer-protein conjugates, even if individual bonding events might be unstable.^20^ When excess 2-PCA was reacted with hydroxylamine a shift in equilibrium was observed and the protein released from the polymer matrix, emphasising this point. We therefore sought to understand the effect an excess of competitive N-termini might have on the stability of protein conjugates formed with 1–7.

**Fig. 6:**
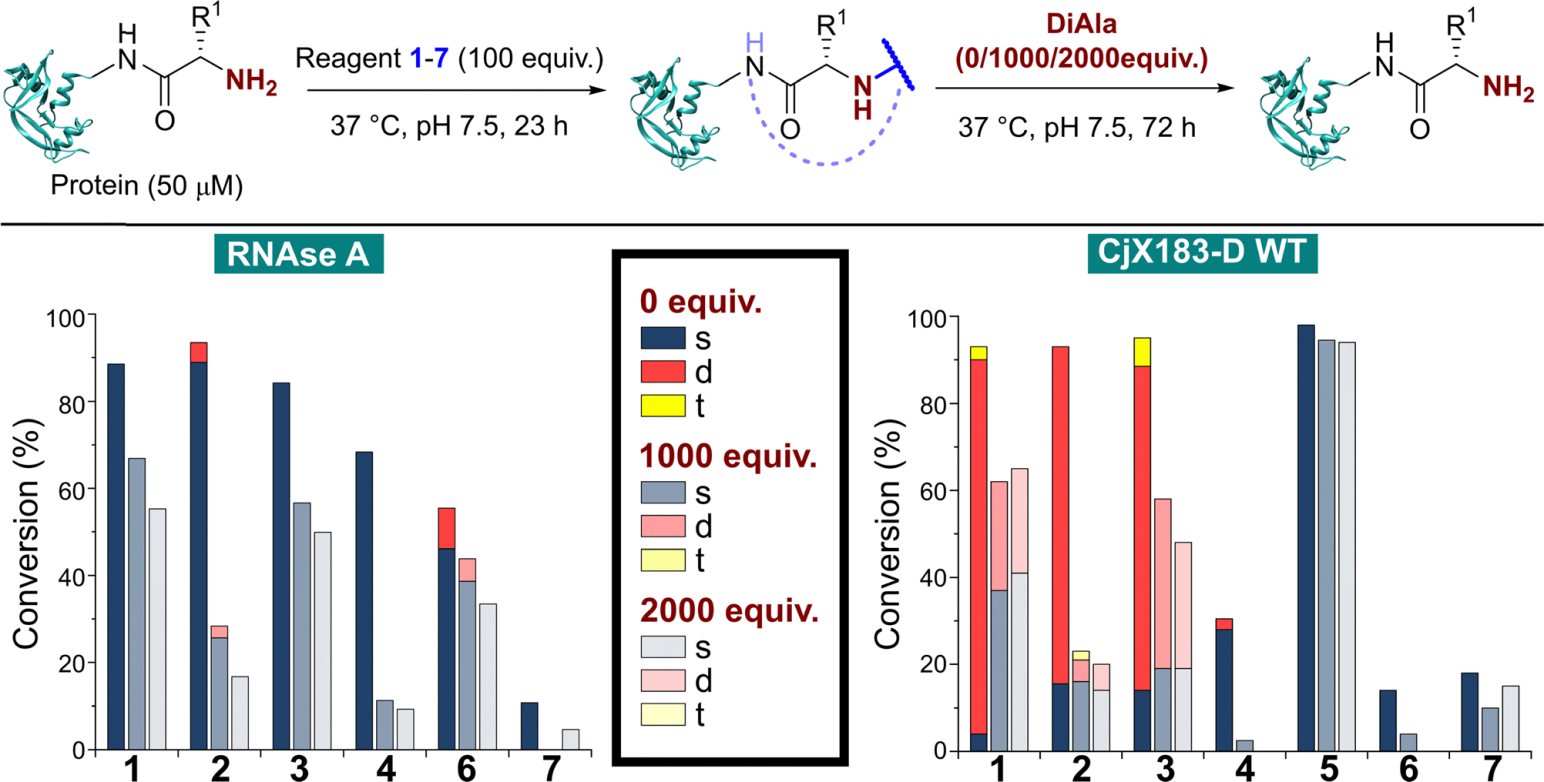
Stability of protein conjugates in the presence of DiAla as a competitive N-terminus mimic. After initial conjugation at 37 °C for 23 h, the stated number of equivalents of DiAla (w.r.t. protein) were added without purification, and the samples incubated at 37 °C for 72 h.

To do this, RNase A was first modified with 100 equiv. of reagents 1–7 as described above. Without purification, the dipeptide Ala–Ala (DiAla) was added as a simple model competitor, possessing an α-amido amine at varying concentrations (Fig. 6, 1000, or 2000 equiv. w.r.t. protein; 10 or 20 equiv. w.r.t. reagent 1–7). This large excess was expected to serve two roles, first reacting with excess reagent and nullifying its presence, and secondly scavenging any that was released as a result of dynamic binding at the N-terminus. Reactions were incubated at 37 °C for 24–72 h, and in all cases a large drop in protein conjugation was observed relative to the control (0 equiv. DiAla). Though a decrease in stability was observed upon doubling the DiAla concentration, the effect was minimal implying a dissociative mechanism of cleavage. As seen during the stability studies described above, conjugates formed from 2-PCA 2 and TA4C 4 were particularly susceptible to cleavage, while conjugates formed with Ox 6 were less susceptible to competition. In contrast, when these experiments were repeated with CjX183-D 6 was completely cleaved over 72 h, again demonstrating high protein dependence.

For 2-EBA 5, the weak protein signal that resulted from the use of 100 equiv. of reagent necessitated the experiment be performed with just 25 equiv. w.r.t. RNase A, followed by incubation with 250, or 500 equiv. of DiAla. Under these conditions minimal protein modification was observed for 6 and 7 and so comparisons to these reagents could not be made. However, relative to 1–4 conjugates formed with 5 were found to be the most stable and least sensitive to competitive cleavage (See ESI† Table S5 and Fig. S9).

### Kinetics of N-terminal modification

**Fig. 7:**
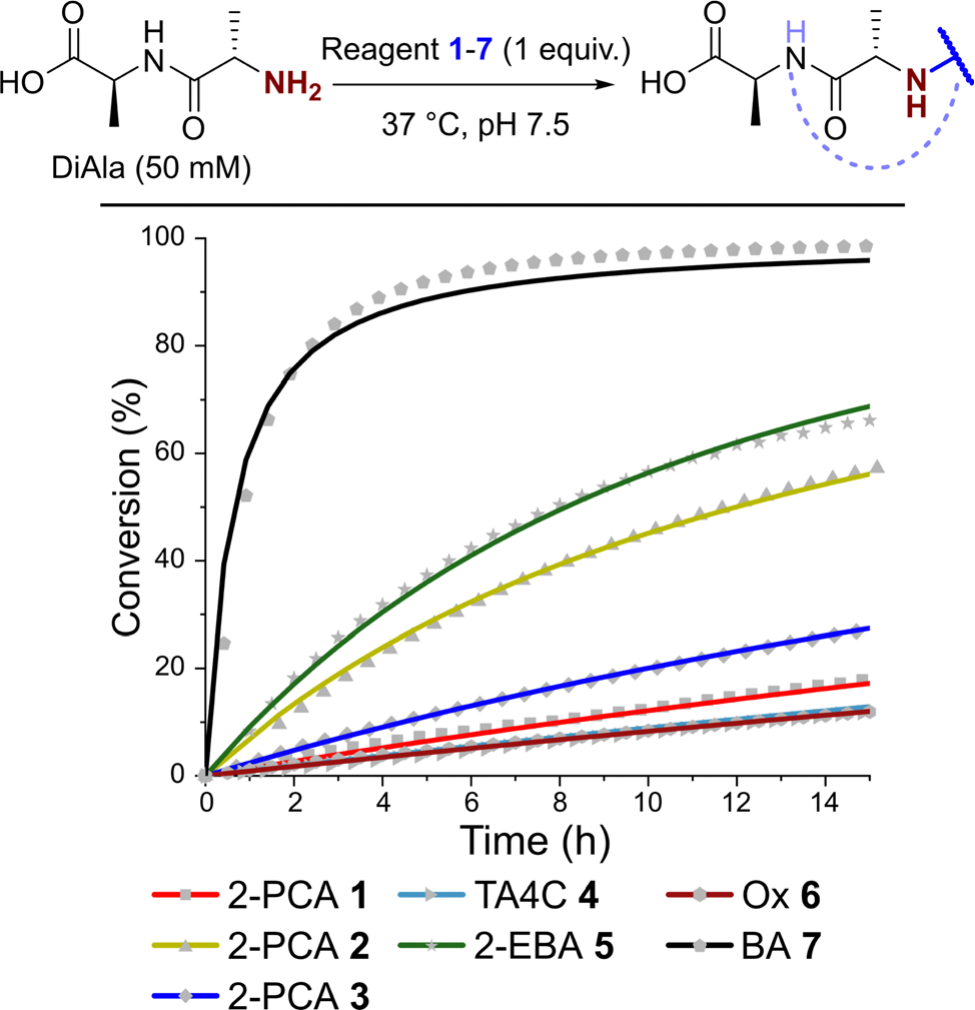
Plots of conversion against time for the reactions of DiAla with reagents 1–7 under second order conditions at a concentration of 50 mM, as measured by ^1^H NMR analysis. Fits are based on second order reversible or irreversible models.

Having observed N-terminal conjugate instability for all of the reagents tested, we sought to gain better insight into the underlying conjugation chemistry. DiAla (50 mM), as a small molecule mimic of a protein N-terminus, was therefore reacted with reagents 1–7 (50 mM) under second-order reaction conditions at 37 °C in pH 7.5 sodium phosphate buffer, and conjugate formation was followed over time by ^1^H NMR spectroscopy (Fig. 7).

Data were then fit to a reversible or irreversible second-order kinetic model, as appropriate, allowing us to calculate forward and backward rate constants, *k*_1_ and *k*_−1_ respectively, as well as the dissociation constant, *K*_d_, for each reaction where appropriate (Table 2).^23^ These studies revealed that reactions between the N-terminus of DiAla and reagents 1–6 were all fairly slow (*k*_1_ < 5 × 10^−4^ M^−1^ s^−1^) relative to many other bioconjugation strategies,^4^ explaining the need for large excesses of reagent and long reaction times to drive significant conversions. Under these conditions, the data fit an irreversible model for reagents 1, 3, and 6, though this does not preclude reversibility at levels below our threshold for detection (*K*_d_ < 20 μM). Interestingly, our data conflicts with those of Tang *et al.* who reported a second order rate constant of ∼1 × 10^−2^ for the reaction of an unmodified Ox reagent with the tripeptide GAF. This may indicate that glycine possesses particularly high reactivity with Ox reagents, relative to bulkier α-substituted amino acids, but further studies are required to investigate this effect.

**Table 2:** Tabulated forward (*k*_1_) and backward (*k*_−1_) rate constants, and dissociation constants (*K*_d_) where relevant, for the reaction of reagents 1–7 with DiAla, as calculated by ^1^H NMR

	*k*_1_/M^−1^ s^−1^	*k*_−1_/s^−1^	*K*_d_/mM
1	7.6 ± 0.1 × 10^−5^	—	—
2	4.2 ± 0.02 × 10^−4^	2.5 ± 0.3 × 10^−7^	0.58
3	1.4 ± 0.01 × 10^−4^	—	—
4	5.4 ± 0.1 × 10^−5^	2.3 ± 0.2 × 10^−7^	4.27
5	5.4 ± 0.04 × 10^−4^	1.6 ± 0.1× 10^−6^	2.91
6	5.0 ± 0.1 × 10^−5^	—	—
7	5.1 ± 0.3 × 10^−3^	—	—

In contrast, for 2-PCA 2 (*K*_d_ ∼ 600 μM), TA4C 4 (*K*_d_ ∼ 4 mM), and 2-EBA 5 (*K*_d_ ∼ 3 mM) significant levels of reversibility were observed. These results partly rationalise the protein conjugate stability data we observed, whereby conjugates were slowly cleaved following the removal of excess reagent, with 2 and 4 being the most sensitive in many cases (though not always). The high *K*_d_ recorded for 2-EBA 5 was more surprising, given protein modification with 5 seemed to lead to relatively stable conjugation (albeit with challenges associated with controlling the site of modification). This highlights the challenges faced in extrapolating results from small molecule models to challenging protein substrates, where effects from the local environment can make a major contribution, both to stability and instability.

## Conclusions

We have carried out a detailed comparative study of the current leading strategies for N-terminal protein modification, probing the conversion, selectivity, stability, and kinetics of modification under standardised conditions. While protein modification chemistries are typically developed on small molecule or peptide model systems, our results emphasise the important influence protein structure plays in dictating conjugation efficiency – in no case was one reagent found to outperform the others, and large differences in protein-dependent conjugate behaviour were observed throughout the study. Predicting the most suitable reagent for efficient modification of a target protein is therefore challenging, with no ‘one size fits all’ reagent for N-terminal modification.

Multiple factors are at play, likely intertwined to act in both a synergistic and antagonistic fashion, including steric accessibility at the protein surface, interactions between reagents and the neighbouring environment, and the influence of localized differences in pH/p*K*_a_. For aldehydes 1–5 and 7, this variability is exacerbated by the nature of the reagents themselves, with differing propensities to equilibrate between aldehyde and hydrate, and the dynamic nature of imine chemistry potentially leading to different product structures being formed depending on the local environment. It is therefore critical that results from small molecule and peptide models are adequately validated on a range of protein substrates. Our results advocate for the screening of a panel of reagents to ensure optimal conjugation is realised. Moreover, by highlighting the limitations of current methods, particularly with regards to conjugate stability, this study supports the need for further investigation in the field, to provide new fast, selective, and stable N-terminal modification strategies that address these limitations. Efforts to develop such methods are currently underway in our lab.

## Author contributions

LJB performed all chemical synthesis and protein modifications. NDJY undertook plasmid mutagenesis and protein expression, under the supervision of MAF and AP. GRH supplied plasmids and protocols for CjX183-D expression. PGG and CDS supervised the study. CDS developed the project concept, managed the study. LJB and CDS wrote the manuscript, with all authors contributing to editing.

## Conflicts of interest

The authors declare no competing financial interests.

## Supplementary Material

CB-004-D2CB00203E-s001
