# Supplementary material for: Selectivity and stability of N-terminal targeting protein modification chemistries
Source: RSC Chem Biol. 2022 Nov 17;4(1):56–64. doi: 10.1039/d2cb00203e (PMC9811658; doi:10.1039/d2cb00203e)

Electronic Supplementary Material (ESI) for RSC Chemical Biology.
This journal is © The Royal Society of Chemistry 2022

Selectivity and stability of N-terminal targeting protein modification chemistries

Lydia J. Barber,[a,b,c] Nicholas D. Yates,[a,b] Martin A. Fascione,[a,b] Alison Parkin,[a] Glynn R. Hemsworth,[d] Paul G. Genever,[b,c] and Christopher D. Spicer*[a,b,c]

[a]Department of Chemistry, University of York, Heslington, YO10 5DD, UK
[b]York Biomedical Research Institute, University of York, Heslington, YO10 5DD, UK
[c]Department of Biology, University of York, Heslington, YO10 5DD, UK
[d]Astbury Centre for Structural Molecular Biology and School of Molecular and Cellular Biology, Faculty of Biological Sciences, University of Leeds, Leeds LS2 9JT, UK

* chris.spicer@york.ac.uk

Table of contents

S2 General considerations

S3 Reagent synthesis

S18 Protein expression

S22 Protein modification

S40 Conjugate stability

S44 Stability to competitive peptide

S47 Kinetic studies

S50 References

S52 NMR spectra of novel compounds

General considerations

Proton nuclear magnetic resonance (^{1}H NMR) spectra were recorded on a Bruker AVII (300 MHz), Jeol ECX-400 (400 MHz), Bruker AVIIIHD (500 MHz) or Bruker AVIIIHD (600 MHz) spectrometer. Carbon nuclear magnetic resonance (^{13}C NMR) spectra were recorded on a Jeol ECX-400 (100 MHz) or Bruker AVII (75 MHz) spectrometer. NMR shifts were assigned using COSY, HSQC and HMBC spectra. All chemical shifts are quoted on the δ scale in ppm using residual solvent as the internal standard (^{1}H NMR: $CDCl_3$ = 7.26; CD_3OD = 3.31; D_2O = 4.69; DMSO-d_6 = 2.50 and ^{13}C NMR: $CDCl_3$ = 77.16, CD_3OD = 49.00, DMSO-d_6 = 39.52). Coupling constants (J) are reported in Hz with the following splitting abbreviations: s = singlet, d = doublet, t = triplet, q = quartet, m = multiplet, app = apparent, br = broad. Melting points (m.p.) were recorded on a Gallenkamp melting point apparatus. Infrared (IR) spectra were recorded on a Perkin Elmer UATR Two FT-IR spectrometer or a Bruker Alpha II ATR spectrometer with Opus build 8.5.29. High resolution electrospray ionisation (ESI) mass spectra (HRMS) were recorded on a Bruker Compact TOF-MS or a Jeol AccuTOF GCx-plus spectrometer. Nominal and exact *m/z* values are reported in Daltons (Da).

Thin layer chromatography (TLC) was carried out using aluminium backed sheets coated with 60 F_{254} silica gel (Merck). Visualization of the silica plates was achieved using a UV lamp (λ_{max} = 254 nm), potassium permanganate (5% $KMnO_4$ in 1M NaOH with 5% potassium carbonate), or ninhydrin (1.5% ninhydrin, 3% AcOH in *n*-butanol). Flash column chromatography was carried out using Geduran Si 60 (40-63 µm) (Merck). Mobile phases are reported as % volume of more polar solvent in less polar solvent. Anhydrous solvents were dried over a PureSolv MD 7 Solvent Purification System. Deionized water was used for chemical reactions and for protein manipulations. All other solvents were used as supplied (Analytical or HPLC grade), without prior purification. Reagents were purchased from Sigma-Aldrich, VWR, or Fluorochem and used as supplied, unless otherwise indicated. Brine refers to a saturated solution of sodium chloride. Petrol refers to the fraction of petroleum ether boiling in the range 40-60 °C. Anhydrous magnesium sulfate ($MgSO_4$) was used as the drying agent after reaction workup unless otherwise stated. RNase A from bovine pancreas (powder, 50 units/mg protein) and myoglobin from equine skeletal muscle (powder, 95-100%) were purchased from Sigma Aldrich, and Clostripain histolyticum (Endoproteinase-Arg-C) was purchased from BioServ UK Limited.

Liquid chromatography-mass spectrometry (LC-MS) was performed on a HCTultra ETD II ion trap spectrometer, coupled to an Ultimate300 HPLC using an Accucore C18 column (150 × 2.1 mm, 2.6 µm particle size). Water (solvent A) and acetonitrile (solvent B), both containing 0.1% formic acid, were used as the mobile phase at a flow rate of 0.3 mL min^{-1}. LC traces were measured via UV absorption at 220, 270, and 280 nm. The gradient was programmed as shown below:

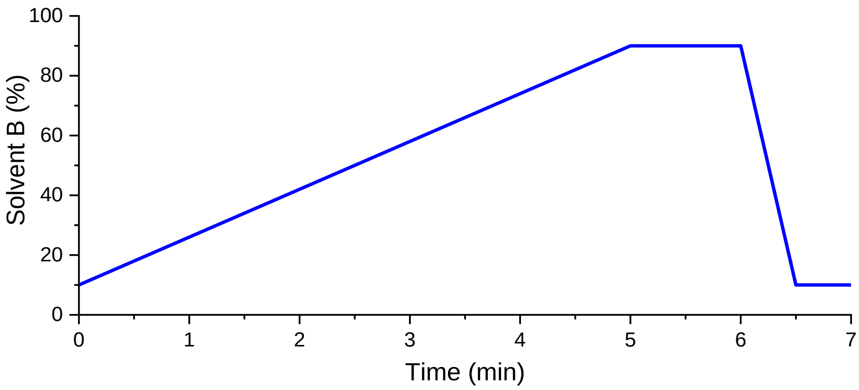

Spectra were analysed using the Bruker Data Analysis 4.4 software. Spectra were charge deconvoluted using ESI Compass 1.3 for RNase A (13000-16000 Da), myoglobin (16000-20000 Da), CjX183-D WT and R51K (11000-15000 Da). Clostripain light chain (LC) spectra were charge deconvoluted using MaxEnt (8000-20000 Da). Data are presented showing the raw ion series MS data on the left, and the deconvoluted spectra on the right. Expected masses were calculated relative to reported [**SM**+H]$^+$ values of 13681 Da (RNase A)[1], 16951 Da (myoglobin)[2] and 14941 Da (Clostripain LC)[3]. Observed masses of CjX183-D WT and R51K mutant were typically ca. 10 Da higher than theoretical masses (11226 Da and 11198 Da respectively), so expected masses for CjX183-D were calculated from the smallest species present in each individual sample. **SM** = unmodified starting material, **P** = modified product, s = single modification, d = double modification, t = triple modification, q = quadruple modification, qu = quintuple modification, se = sextuple modification, sp = septuple modification.

1. Reagent synthesis

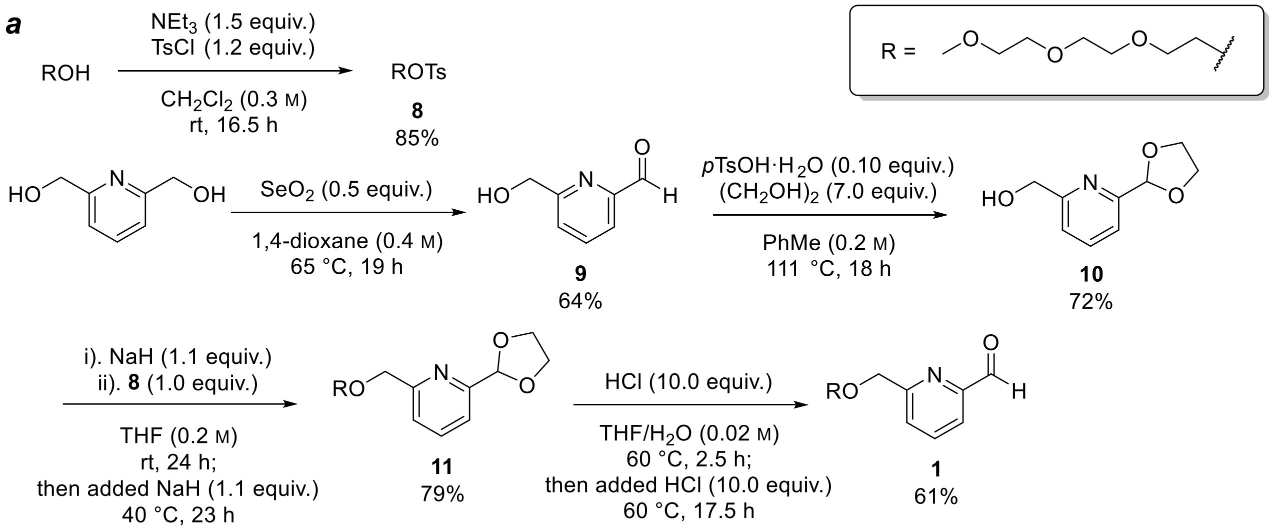

b

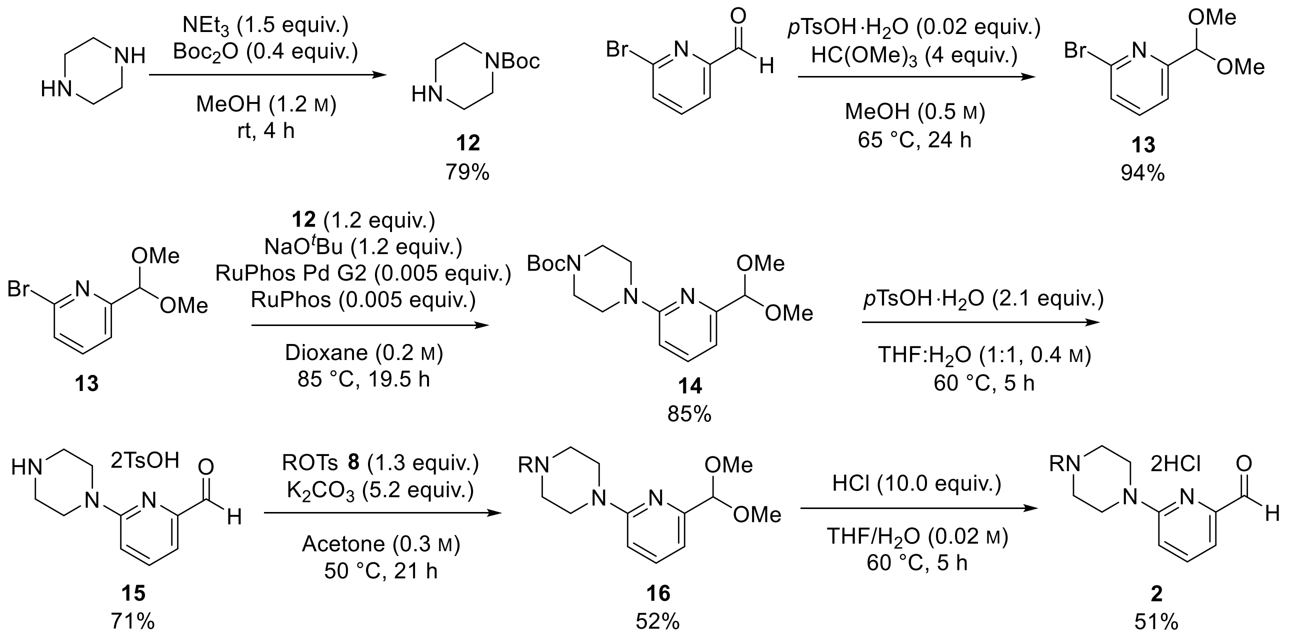
NEt3 (1.5 equiv.)
Boc2O (0.4 equiv.)
MeOH (1.2 M)
rt, 4 h
12
79%
pTsOH·H2O (0.02 equiv.)
HC(OMe)3 (4 equiv.)
MeOH (0.5 M)
65 °C, 24 h
13
94%
12 (1.2 equiv.)
NaOtBu (1.2 equiv.)
RuPhos Pd G2 (0.005 equiv.)
RuPhos (0.005 equiv.)
Dioxane (0.2 M)
85 °C, 19.5 h
13
14
85%
pTsOH·H2O (2.1 equiv.)
THF:H2O (1:1, 0.4 M)
60 °C, 5 h
2TsOH
15
71%
ROTs 8 (1.3 equiv.)
K2CO3 (5.2 equiv.)
Acetone (0.3 M)
50 °C, 21 h
16
52%
HCl (10.0 equiv.)
THF/H2O (0.02 M)
60 °C, 5 h
2HCl
2
51%

c

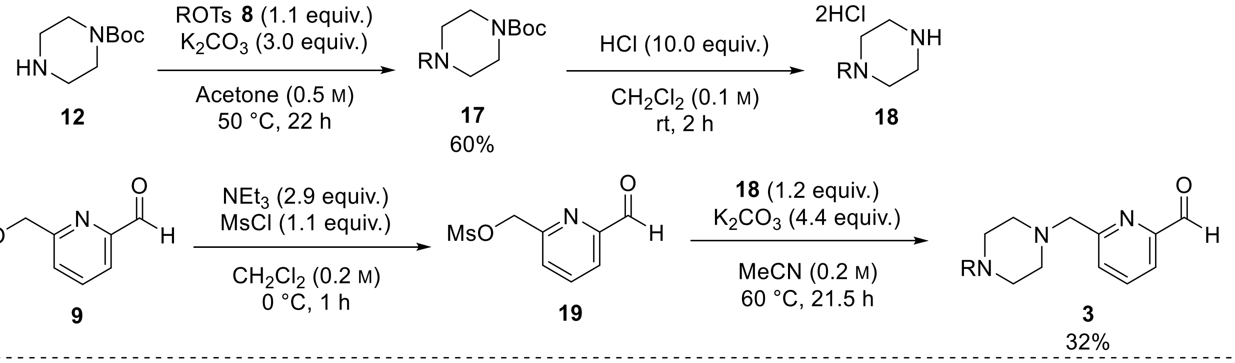
12
ROTs 8 (1.1 equiv.)
K2CO3 (3.0 equiv.)
Acetone (0.5 M)
50 °C, 22 h
17
60%
HCl (10.0 equiv.)
CH2Cl2 (0.1 M)
rt, 2 h
2HCl
18
9
NEt3 (2.9 equiv.)
MsCl (1.1 equiv.)
CH2Cl2 (0.2 M)
0 °C, 1 h
19
18 (1.2 equiv.)
K2CO3 (4.4 equiv.)
MeCN (0.2 M)
60 °C, 21.5 h
3
32%

d

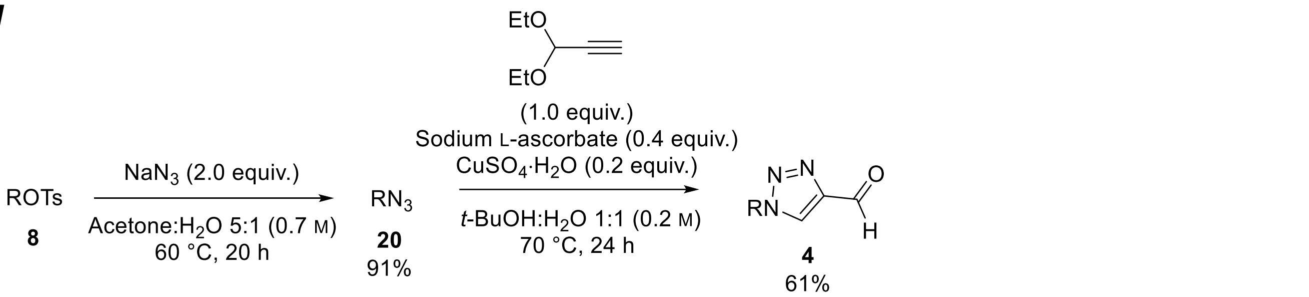
ROTs
8
NaN3 (2.0 equiv.)
Acetone:H2O 5:1 (0.7 M)
60 °C, 20 h
RN3
20
91%
(1.0 equiv.)
Sodium L-ascorbate (0.4 equiv.)
CuSO4·H2O (0.2 equiv.)
t-BuOH:H2O 1:1 (0.2 M)
70 °C, 24 h
4
61%

e

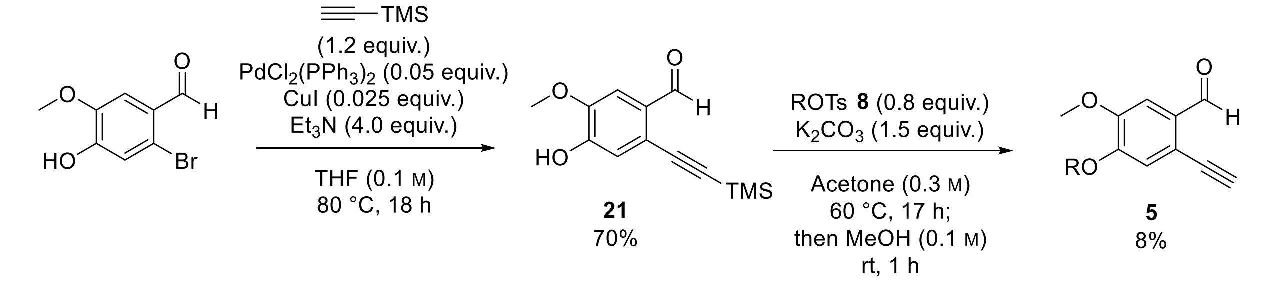
TMS
(1.2 equiv.)
PdCl2(PPh3)2 (0.05 equiv.)
CuI (0.025 equiv.)
Et3N (4.0 equiv.)
THF (0.1 M)
80 °C, 18 h
21
70%
ROTs 8 (0.8 equiv.)
K2CO3 (1.5 equiv.)
Acetone (0.3 M)
60 °C, 17 h;
then MeOH (0.1 M)
rt, 1 h
5
8%

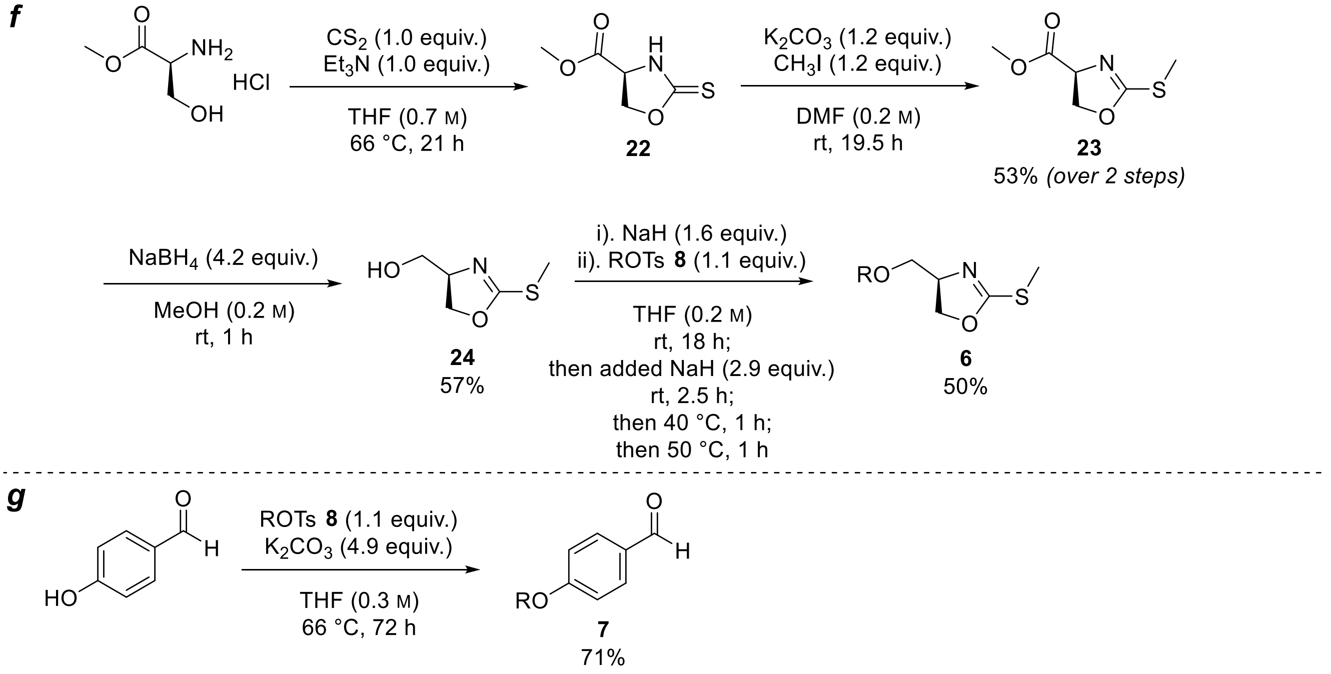

Scheme S1. Synthetic route for reagents: (*a*) 2-PCA **1**; (*b*) 2-PCA **2;** (*c*) 2-PCA **3**; (*d*) TA4C **4**; (*e*) 2-EBA **5**; (*f*) Ox **6**; and (*g*) BA **7**.

(a) *2-PCA* ***1***

2-(2-(2-Methoxyethoxy)ethoxy)ethyl 4-methylbenzenesulfonate (8)

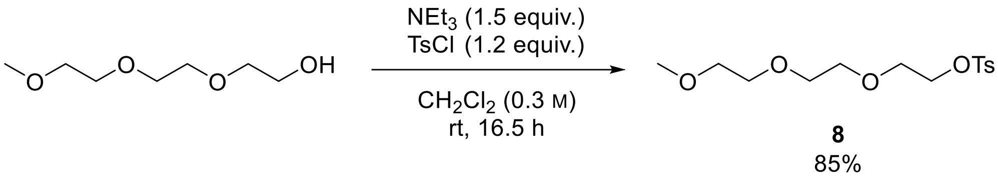

A solution of *p*-toluenesulfonyl chloride (1.88 g, 9.9 mmol, 1.2 equiv.) in anhydrous CH_2Cl_2 (5.8 mL) was added to a solution of triethylene glycol monomethyl ether (1.3 mL, 8.1 mmol, 1.0 equiv.) and triethylamine (1.7 mL, 12.2 mmol, 1.5 equiv.) in anhydrous CH_2Cl_2 (19.3 mL, 0.3 M) under nitrogen, and the reaction mixture was stirred at room temperature for 16.5 h. The reaction was then washed sequentially with saturated aqueous $NaHCO_3$ solution (10 mL) and brine (10 mL), dried over $MgSO_4$, filtered, and the solvent removed under reduced pressure. The resulting pale yellow oil was purified by flash column chromatography (66% EtOAc:Petrol, R_f 0.52 in 75% EtOAc:Petrol), and pure fractions were concentrated under reduced pressure to afford the title compound (2.20 g, 6.9 mmol, 85%) as a pale yellow oil with spectroscopic data in accordance with the literature.[4] **^{1}H NMR** (300 MHz, $CDCl_3$) $\delta_{H:}$ 2.44 (3H, s, ArMe), 3.36 (3H, s, OMe), 3.49-3.71 (10H, m, -OC$\underline{H}_2$), 4.15 (2H, t, J = 4.9 Hz, -C$\underline{H}_2$OTs), 7.33 (2H, d, J = 8.3 Hz, Ar$\underline{H}$2), 7.79 (2H, d, J = 8.3 Hz, Ar$\underline{H}$3).

6-(Hydroxymethyl)-2-pyridinecarboxaldehyde (9)

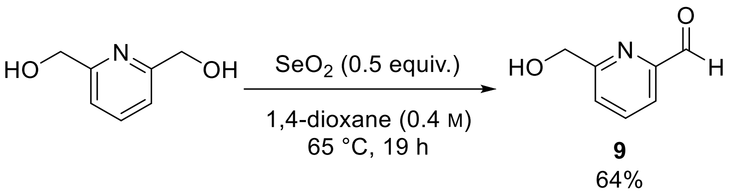

Selenium dioxide (0.59 g, 5.3 mmol, 0.5 equiv.) was added to a solution of 2,6-pyridinedimethanol (1.50 g, 10.8 mmol, 1.0 equiv.) in 1,4-dioxane (30 mL, 0.4 M) at room temperature. The resulting mixture was sonicated for 2 min and then stirred at 65 °C for 19 h. After cooling to room temperature, CH_2Cl_2 (50 mL) was added, the resulting mixture was filtered through Celite, and the filtrate was concentrated under reduced pressure to afford a yellow oil. The residue was purified by flash column chromatography (2.5% MeOH:CH_2Cl_2, R_f 0.20), and pure fractions were concentrated under reduced pressure to afford the title compound (0.95 g, 6.9 mmol, 64%) as a yellow oil with spectroscopic data in accordance with the literature.[3] **^{1}H NMR** (300 MHz, $CDCl_3$) $\delta_{H:}$ 3.59 (1H, br s, -OH), 4.87 (2H, s, -CH_2-), 7.47-7.57 (1H, m, ArH4), 7.83-7.93 (2H, m, ArH3, ArH5), 10.08 (1H, s, -CHO).

(6-(1,3-Dioxolan-2-yl)pyridin-2-yl)methanol (10)

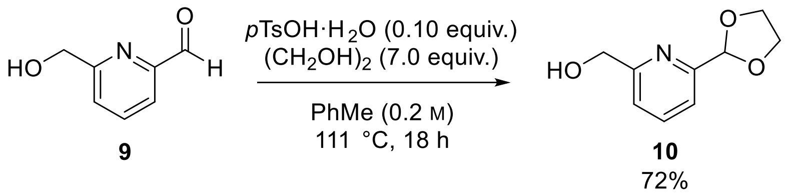

A mixture of **9** (3.56 g, 26 mmol, 1.0 equiv.), *p*-toluenesulfonic acid monohydrate (0.49 g, 2.6 mmol, 0.10 equiv.), and ethylene glycol (10.1 mL, 181 mmol, 7.0 equiv.) in toluene (150 mL, 0.2 M) was refluxed under Dean-Stark conditions for 18 h. After cooling to room temperature, the reaction was concentrated under reduced pressure and the residue purified by flash column chromatography (0-2% MeOH:CH_2Cl_2, R_f 0.38 in 100% EtOAc). Pure fractions were concentrated under reduced pressure to afford the title compound (3.38 g, 18.7 mmol, 72%) as a yellow oil. **^{1}H NMR** (400 MHz, $CDCl_3$) $\delta_{H:}$ 4.03-4.19 (5H, m, -OCH_2 & -OH), 4.76 (2H, s, ArCH_2), 5.84 (1H, s, -CHO_2R), 7.26 (1H, d, J = 7.8 Hz, ArH5), 7.44 (1H, d, J = 7.3 Hz, ArH3), 7.67 (1H, dd, J = 7.8, 7.3 Hz, ArH4); **^{13}C NMR** (101 MHz, $CDCl_3$) $\delta_{C:}$ 64.42 (ArCH_2), 65.80 (-OCH_2), 103.68 (-CHO_2R), 119.31 (ArC3), 121.09 (ArC5), 137.77 (ArC4), 156.19 (ArC2), 159.41 (ArC6); **ν_{max}** (ATR, cm^{-1}) 3350, 2890, 1598, 1455, 1368, 1212, 1104, 1027, 991, 945, 781, 649; **HRMS** (ESI^+) $C_9H_{11}NO_3$ $[M+Na]^+$ found 204.0632, calculated 204.0631.

2-(1,3-Dioxolan-2-yl)-6-(2,5,8,11-tetraoxadodecyl)pyridine (11)

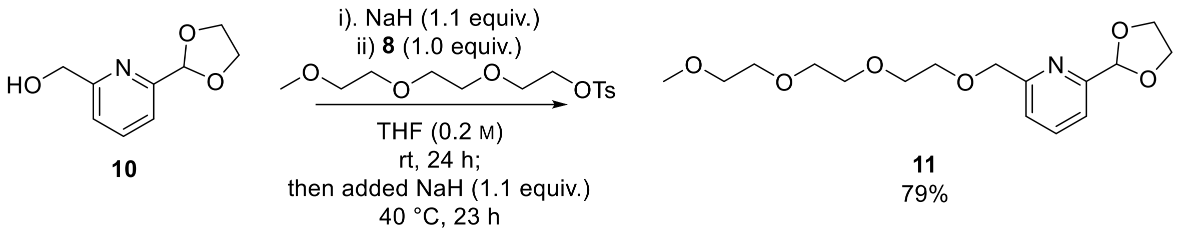

Sodium hydride (60%, 12 mg, 330 µmol, 1.1 equiv.) was added slowly to a solution of **10** (54 mg, 300 µmol, 1.0 equiv.) in anhydrous THF (1.4 mL, 0.2 M), and after 10 min a solution of compound **8** (94 mg, 300 µmol, 1.0 equiv.) in anhydrous THF (0.5 mL) was added. The reaction mixture was then stirred at room temperature for 24 h under nitrogen. At this point TLC indicated that the reaction was incomplete, so another portion of sodium hydride (60%, 12 mg, 330 µmol, 1.1 equiv.) was added, and the reaction mixture was stirred at 40 °C for 23 h. After cooling to room temperature, the reaction was quenched through careful dropwise addition of water (5 mL), and the aqueous extracted with ethyl acetate (3 × 20 mL). The combined organics were dried over $MgSO_4$, filtered, and concentrated under reduced pressure. The resulting yellow oil was purified by flash column chromatography (75-100% EtOAc:Petrol, then 0-2.5% MeOH:EtOAc, R_f 0.14 in 100% EtOAc). Pure fractions were concentrated under reduced pressure to afford the title compound (77 mg, 235 µmol, 79%) as a yellow oil. **^{1}H NMR** (300 MHz, $CDCl_3$) δ_H: 3.35 (3H, s, Me), 3.49-3.57 (2H, m, -C$\underline{H}_2$OMe), 3.59-3.76 (10H, m, -C$\underline{H}_2$C$\underline{H}_2$OC$\underline{H}_2$C$\underline{H}_2$OC$\underline{H}_2$$CH_2$OMe), 3.99-4.21 (4H, m, -C$\underline{H}_2$OCHAr), 4.71 (2H, s, -C$\underline{H}_2$Ar), 5.81 (1H, s, -C$\underline{H}$Ar), 7.42 (1H, dd, J = 7.7, 1.1 Hz, Ar$\underline{H}$3), 7.49 (1H, dd, J = 7.7, 1.1 Hz, Ar$\underline{H}$5), 7.74 (1H, dd, J_1 = J_2 = 7.7 Hz, Ar$\underline{H}$4); **^{13}C NMR** (75 MHz, $CDCl_3$) δ_C: 59.10 (Me), 65.63 (-$\underline{C}H_2$OCHAr), 70.38 (-$\underline{C}H_2$OCH_2Ar), 70.62 (-$\underline{C}H_2$-), 70.63 (-$\underline{C}H_2$-), 70.73 (-$\underline{C}H_2$-), 70.77 (-$\underline{C}H_2$-), 72.03 (-$\underline{C}H_2$OMe), 73.83 (-$\underline{C}H_2$Ar), 103.52 (-$\underline{C}$HAr), 119.18 (Ar$\underline{C}$3), 121.65 (Ar$\underline{C}$5), 137.68 (Ar$\underline{C}$4), 156.19 (Ar$\underline{C}$2), 158.58 (Ar$\underline{C}$6); **v_{max}** (ATR, cm^{-1}) 2875, 1596, 1580, 1459, 1350, 1280, 1247, 1200, 1099, 1027, 988, 966, 945, 885, 851, 804, 751, 725, 651, 548; **HRMS** (ESI^+) $C_{16}H_{26}NO_6$ $[M+H]^+$ found 328.1756, calculated 328.1755.

6-(2,5,8,11-Tetraoxadodecyl)picolinaldehyde (1)

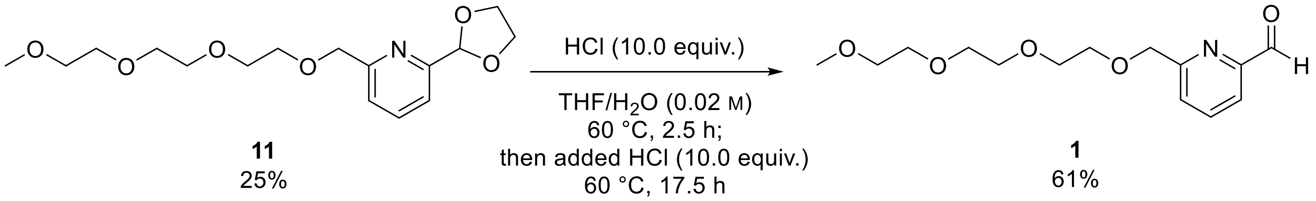

HCl (2 M in water, 1 mL, 2.0 mmol, 10.0 equiv.) was added to a solution of compound **11** (65 mg, 200 µmol, 1.0 equiv.) in THF (12 mL, 0.02 M), and the reaction mixture was stirred at 60 °C for 2.5 h under nitrogen. At this time TLC indicated that the reaction was incomplete, so another portion of HCl (2 M in water, 1 mL, 2.0 mmol, 10.0 equiv.) was added and the reaction mixture was stirred at 60 °C for a further 17.5 h. After cooling to room temperature, the solvent was removed under reduced pressure and the residue obtained was purified by flash column chromatography (10% MeOH:CH_2Cl_2, R_f 0.24). ^{1}H NMR revealed the fractions collected were impure, so the resulting yellow residue was purified by flash column chromatography (75% EtOAc:Petrol).

Pure fractions were concentrated under reduced pressure to afford the title compound (34 mg, 0.12 mmol, 61%) as a yellow oil. **^{1}H NMR** (400 MHz, DMSO-d_6) δ_H: 3.22 (3H, s, Me), 3.38-3.44 (2H, m, -$C\underline{H}_2OMe$), 3.48-3.56 (6H, m, -$C\underline{H}_2C\underline{H}_2OC\underline{H}_2CH_2OMe$), 3.58-3.64 (2H, m, -$C\underline{H}_2CH_2OCH_2Ar$), 3.64-3.70 (2H, m, -$C\underline{H}_2OCH_2Ar$), 4.70 (2H, s, -$C\underline{H}_2Ar$), 7.75 (1H, d, J = 7.7 Hz, Ar$\underline{H}$5), 7.85 (1H, d, J = 7.7 Hz, Ar$\underline{H}$3), 8.06 (1H, dd, J_1 = J_2 = 7.7 Hz, Ar$\underline{H}$4), 9.96 (1H, s, $C\underline{H}O$); **^{13}C NMR** (101 MHz, DMSO-d_6) δ_C: 58.06 (Me), 69.62 (-$\underline{C}H_2CH_2$-), 69.74 (-$\underline{C}H_2CH_2OCH_2Ar$), 69.81 (-$\underline{C}H_2CH_2$-), 69.89 (-$\underline{C}H_2CH_2$- and -$\underline{C}H_2OCH_2Ar$), 71.29 (-$\underline{C}H_2OMe$), 72.68 (-$\underline{C}H_2Ar$), 120.50 (Ar$\underline{C}$3), 125.82 (Ar$\underline{C}$5), 138.40 (Ar$\underline{C}$4), 151.67 (Ar$\underline{C}$2), 159.42 (Ar$\underline{C}$6), 193.55 ($\underline{C}HO$); **$\nu_{max}$** (ATR, cm^{-1}) 2923, 2871, 1713, 1593, 1457, 1351, 1250, 1210, 1102, 992, 943, 851, 799, 782, 661, 619; **HRMS** (ESI$^+$) $C_{14}H_{22}NO_5$ [M+H]$^+$ found 284.1497, calculated 284.1492.

(b) *2-PCA* ***2***

tert-Butyl piperazine-1-carboxylate (12)

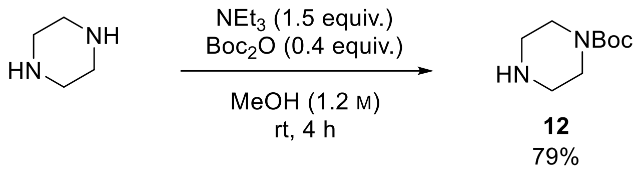

A solution of di-*tert*-butyl dicarbonate (1.14 g, 5.2 mmol, 0.4 equiv.) in methanol (5 mL) was added dropwise to a solution of triethylamine (2.8 mL, 20.1 mmol, 1.5 equiv.) and piperazine (1.16 g, 13.4 mmol, 1.0 equiv.) in methanol (12 mL, 1.2 M) over 5 min. The reaction mixture was stirred at room temperature for 4 h and then concentrated under reduced pressure. Ethyl acetate (20 mL) was added and resulting white precipitate was removed by vacuum filtration. The filtrate was washed with water (3 × 5 mL), dried over $MgSO_4$, filtered, and concentrated under reduced pressure to afford the title compound (0.79 g, 4.3 mmol, 79%) as a white solid with spectroscopic data in accordance with the literature.[5] **^{1}H NMR** (300 MHz, $CDCl_3$) δ_H: 1.46 (9H, s, Boc), 2.80 (4H, t, J = 5.3 Hz, -$C\underline{H}_2NBoc$), 3.38 (4H, t, J = 5.3 Hz, -$C\underline{H}_2NH$); **mp** 46-49 °C {Lit.[5] 46 °C}.

2-Bromo-6-(dimethoxymethyl)pyridine (13)

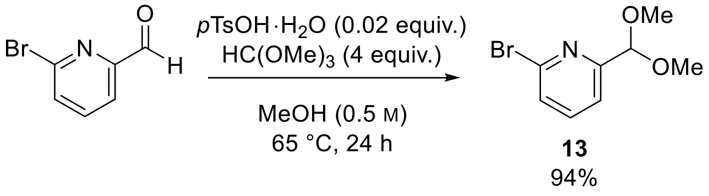

Trimethyl orthoformate (1.2 mL, 10.9 mmol, 4.0 equiv.) was added to a solution of *p*-toluenesulfonic acid monohydrate (10 mg, 0.1 mmol, 0.02 equiv.) and 6-bromo-2-pyridinecarbaldehyde (0.51 g, 2.7 mmol, 1.0 equiv.) in methanol (5 mL, 0.5 M), and the reaction mixture was heated under reflux for 24 h. After cooling to room temperature, the mixture was concentrated under reduced pressure. The residue obtained was partitioned between CH_2Cl_2 (25 mL) and saturated aqueous $NaHCO_3$ solution (20 mL). The layers were

separated, and the aqueous layer was extracted with CH_2Cl_2 (5 × 25 mL). The combined organics were dried over $MgSO_4$, filtered, and concentrated under reduced pressure to afford the title compound (0.59 g, 2.5 mmol, 94%) as a brown oil with spectroscopic data in accordance with the literature.[6] **^{1}H NMR** (300 MHz, $CDCl_3$) δ_H: 3.41 (6H, s, -OMe), 5.30 (1H, s, CH(OMe)$_2$), 7.45 (1H, dd, J = 7.6, 1.2 Hz, ArH5), 7.52 (1H, dd, J = 7.6, 1.2 Hz, ArH3), 7.59 (1H, dd, J_1 = J_2 = 7.6 Hz, ArH4).

***tert*-Butyl 4-(6-(dimethoxymethyl)pyridine-2-yl)piperazine-1-carboxylate (14)**

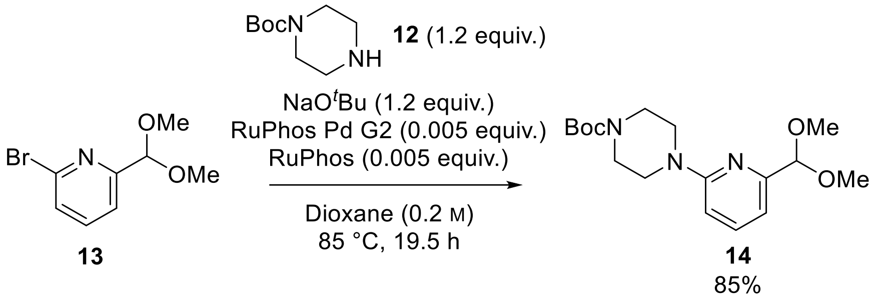

Anhydrous 1,4-dioxane (4 mL, 0.2 M) was added to a mixture of acetal **13** (0.40 g, 1.7 mmol, 1.0 equiv.), piperazine **12** (0.39 g, 2.1 mmol, 1.2 equiv.) and sodium *tert*-butoxide (0.20 g, 2.1 mmol, 1.2 equiv.) under nitrogen. A solution of RuPhos (4 mg, 0.01 mmol, 0.005 equiv.) and RuPhos Pd G2 (7 mg, 0.01 mmol, 0.005 equiv.) in anhydrous 1,4-dioxane (4 mL) was then added and the reaction mixture was stirred at 85 °C for 19.5 h. After cooling to room temperature, the mixture was partitioned between ethyl acetate (30 mL) and water (10 mL). The aqueous layer was extracted with ethyl acetate (3 × 30 mL) and the combined organics were dried over $MgSO_4$, filtered, and concentrated under reduced pressure. The orange residue obtained was purified by flash column chromatography (5% NEt_3, 10% EtOAc:Petrol, R_f 0.31). Pure fractions were concentrated under reduced pressure to afford the title compound (0.50 g, 1.5 mmol, 85%) as a colourless oil with spectroscopic data in accordance with the literature.[7] **^{1}H NMR** (300 MHz, $CDCl_3$) δ_H: 1.48 (9H, s, Boc), 3.40 (6H, s, OMe), 3.53 (8H, app s, -CH_2CH_2NBoc), 5.17 (1H, s, -CH(OMe)$_2$), 6.59 (1H, d, J = 8.5, ArH3), 6.88 (1H, d, J = 7.3 Hz, ArH5), 7.52 (1H, dd, J = 8.5, 7.3 Hz, ArH4).

6-(Piperazin-1-yl)picolinaldehyde ditosylate salt (15)

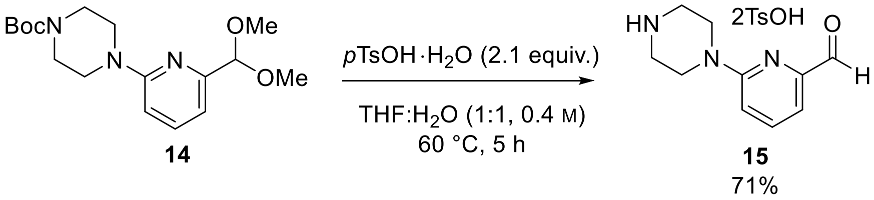

Acetal **14** (55 mg, 163 µmol, 1.0 equiv.) and *p*-toluenesulfonic acid monohydrate (59 mg, 342 µmol, 2.1 equiv.) were dissolved in THF:H_2O (1:1, 0.9 mL, 0.2 M), and the resulting solution was stirred at 60 °C for 5 h. After cooling to room temperature, methanol (2 mL) was added, followed by diethyl ether (30 mL) causing precipitation. The orange residue was collected by filtration, and then dissolved in methanol (10 mL).

Subsequent removal of the solvent under reduced pressure gave the title compound (62 mg, 115 µmol, 71%) as an orange residue with spectroscopic data in accordance with the literature.[7] **^{1}H NMR** (400 MHz, DMSO-d_6) $\delta_{H:}$ 2.28 (6H, s, Me), 3.22 (4H, t, *J* = 5.3 Hz, -CH_2NR), 3.81 (4H, t, *J* = 5.3 Hz, -CH_2NR), 7.13 (4H, d, *J* = 7.7 Hz, TsArH3), 7.25 (1H, d, *J* = 8.6 Hz, ArH3), 7.30 (1H, d, *J* = 7.2 Hz, ArH5), 7.50 (4H, d, *J* = 7.7 Hz, TsArH2), 7.83 (1H, dd, *J* = 8.6, 7.2 Hz, ArH4), 9.82 (1H, s, -CHO).

1-(6-(Dimethoxymethyl)pyridin-2-yl)-4-(2-(2-(2-methoxyethoxy)ethoxy)ethyl)piperazine (16)

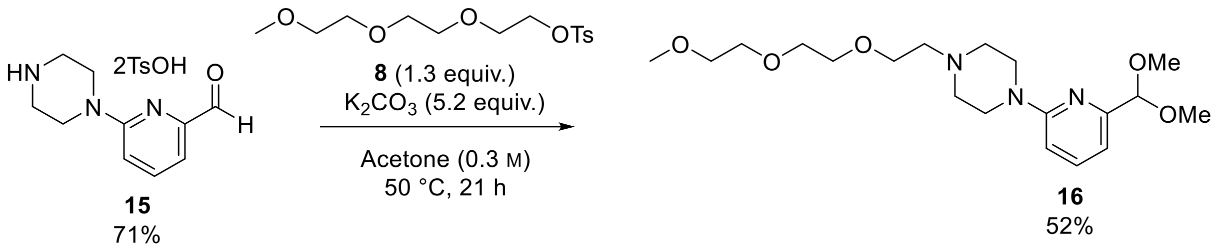

15
71%

16
52%

A solution of compound **15** (79 mg, 248 µmol, 1.4 equiv.) in acetone (0.34 mL) was added to a mixture of K_2CO_3 (133 mg, 887 µmol, 5.3 equiv.) and compound **8** (98 mg, 183 µmol, 1.0 equiv.) in acetone (0.34 mL), and the reaction mixture was stirred at 50 °C for 21 h. The solvent evaporated over this time period and, after cooling to room temperature, the resulting residue was dissolved in CH_2Cl_2 (30 mL). The organics were washed sequentially with water (10 mL) and brine (10 mL), dried over $MgSO_4$, filtered, and concentrated under reduced pressure. The brown residue obtained was purified by flash column chromatography (10% MeOH:CH_2Cl_2, R_f 0.30). Pure fractions were concentrated under reduced pressure to afford the title compound (32 mg, 84 µmol, 52%) as an orange oil, as a 3:97 mixture of aldehyde:dimethyl-acetal. *Spectroscopic data is provided for the major dimethyl-acetal product:* **^{1}H NMR** (300 MHz, $CDCl_3$) $\delta_{H:}$ 2.54-2.71 (6H, m, -$CH_2NCH_2CH_2$NAr), 3.37 (3H, s, -CH_2OMe), 3.38 (6H, s, ArCH(OMe)$_2$), 3.51-3.60 (6H, m, -CH_2NAr & -CH_2OMe), 3.61-3.69 (8H, m, -$CH_2OCH_2CH_2OCH_2CH_2$OMe), 5.15 (1H, s, ArCHOMe), 6.56 (1H, d, *J* = 8.5 Hz, ArH3), 6.83 (1H, d, *J* = 7.3 Hz, ArH5), 7.48 (1H, dd, *J* = 8.5, 7.3 Hz, ArH4); **^{13}C NMR** (75 MHz, $CDCl_3$) $\delta_{C:}$ 45.12 (-CH_2NAr), 53.50 (-CH_2CH_2NAr), 53.87 (ArCHOMe), 57.98 (-$CH_2NCH_2CH_2$NAr), 59.15 (-CH_2OMe), 69.01 (-$CH_2CH_2NCH_2CH_2$NAr), 70.51 (-CH_2-), 70.65 (-CH_2-), 70.74 (-CH_2-), 72.07 (-CH_2OMe), 104.84 (ArCHOMe), 106.62 (ArC3), 110.29 (ArC5), 137.95 (ArC4), 155.47 (ArC6), 159.06 (ArC2); **ν_{max}** (ATR, cm^{-1}) 2827, 1798, 1762, 1593, 1451, 1398, 1334, 1252, 1109, 1052, 1004, 985, 962, 851, 795, 733, 612, 570, 512; **HRMS** (ESI^+) $C_{19}H_{34}N_3O_5$ $[M+H]^+$ found 384.2501, calculated 384.2493.

6-(4-(2-(2-(2-Methoxyethoxy)ethoxy)ethyl)piperazin-1-yl)picolinaldehyde dihydrochloride salt (2)

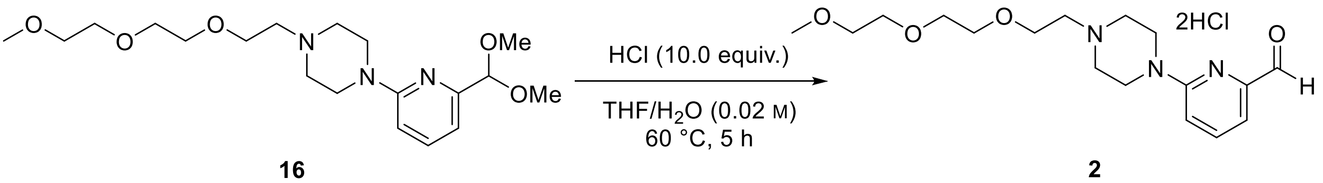

16

2
51%

HCl (2 M in water, 422 µL, 840 µmol, 10.0 equiv.) was added to a solution of compound **16** (32 mg, 84 µmol, 1.0 equiv.) in THF (4.2 mL, 0.02 M) and the reaction mixture was stirred at 60 °C for 5 h. After cooling to room temperature, the mixture was concentrated under reduced pressure and the residue was purified by flash column chromatography (10% MeOH:CH_2Cl_2, R_f 0.30). Pure fractions were concentrated under reduced pressure to afford the title compound (14 mg, 0.04 mmol, 51%) as a yellow oil. **^{1}H NMR** (500 MHz, DMSO-*d*$_6$) $\delta_{H:}$ 3.08-3.19 (2H, m, -C$\underline{H}_2$-), 3.24 (3H, s, Me), 3.29-3.36 (2H, m, -C$\underline{H}_2$-), 3.37-3.65 (12H, m, -NC$\underline{H}_2$C$\underline{H}_2$NAr & -C$\underline{H}_2$-), 3.84-3.90 (2H, m, -C$\underline{H}_2$-), 4.43-4.49 (2H, m, -C$\underline{H}_2$-), 7.28 (1H, *J* = 8.2 Hz, Ar$\underline{H}$3), 7.30 (1H, d, *J* = 7.2 Hz, Ar$\underline{H}$5), 7.84 (1H, dd, *J* = 8.2, 7.2 Hz, Ar$\underline{H}$4), 9.82 (1H, s, C$\underline{H}$O). The compound was used without further analysis or purification and stored at -20 °C due to sensitivity to oxidation.

(c) *2-PCA* ***3***

***Tert*-butyl 4-(2-(2-(2-methoxyethoxy)ethoxy)ethyl)piperazine-1-carboxylate (17)**

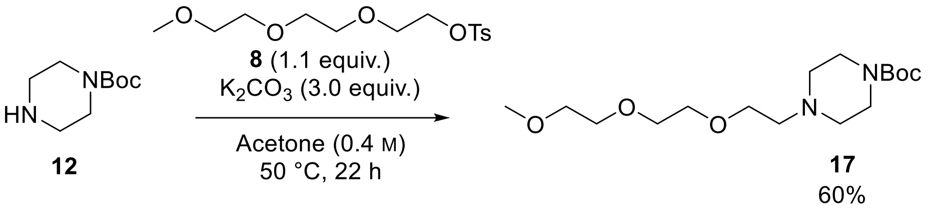

Potassium carbonate (1.28 g, 9.3 mmol, 3.0 equiv.) was added to a solution of piperazine **12** (0.58 g, 3.1 mmol, 1.0 equiv.) in acetone (6.2 mL, 0.4 M) and the reaction mixture was stirred at room temperature for 10 min. A solution of compound **8** (1.08 g, 3.4 mmol, 1.1 equiv.) in acetone (1 mL) was then added and the reaction mixture was heated to 50 °C for 22 h. After cooling to room temperature, the reaction was concentrated under reduced pressure, and the residue dissolved in ethyl acetate (20 mL). The organics were washed with water (2 × 10 mL) and brine (10 mL), dried over Na_2SO_4, filtered, and concentrated under reduced pressure. The residue was purified by flash column chromatography (5% MeOH:CH_2Cl_2, R_f 0.35 in 10% MeOH:CH_2Cl_2). Pure fractions were concentrated under reduced pressure to afford the title compound (0.61 g, 1.8 mmol, 60%) as a yellow oil. **^{1}H NMR** (400 MHz, $CDCl_3$) $\delta_{H:}$ 1.44 (9H, s, Boc), 2.43 (4H, t, *J* = 5.1 Hz, -C$\underline{H}_2$CH$_2$NBoc), 2.58 (2H, t, *J* = 5.8 Hz, -C$\underline{H}_2$NCH$_2$CH$_2$NBoc), 3.37 (3H, s, OMe), 3.42 (4H, t, *J* = 5.1 Hz, -C$\underline{H}_2$NBoc), 3.51-3.55 (2H, m, -OC$\underline{H}_2$-), 3.58-3.65 (8H, m, -OC$\underline{H}_2$-); **^{13}C NMR** (101 MHz, $CDCl_3$) $\delta_{C:}$ 28.54 (C$\underline{Me}_3$), 43.20 (-$\underline{C}$H$_2$NBoc), 53.47 (-$\underline{C}$H$_2$CH$_2$NBoc), 57.93 (-$\underline{C}$H$_2$NCH$_2$CH$_2$NBoc), 59.17 (OMe), 68.92 (-CH$_2$-), 70.47 (-CH$_2$-), 70.65 (-CH$_2$-), 70.74 (-CH$_2$-), 72.05 (-CH$_2$-), 79.69 ($\underline{C}$Me$_3$), 154.87 -N$\underline{C}$O$_2$CMe$_3$); **ν_{max}** (ATR, cm^{-1}) 3574, 2975, 2868, 2816, 1693, 1456, 1418, 1365, 1291, 1244, 1169, 1108, 1024, 1004, 939, 866, 769, 576, 531, 461; **HRMS** (ESI$^+$) $C_{16}H_{33}N_2O_5$ $[M+H]^+$ found 333.2389, calculated 333.2384.

1-(2-(2-(2-Methoxyethoxy)ethoxy)ethyl)piperazine (18)

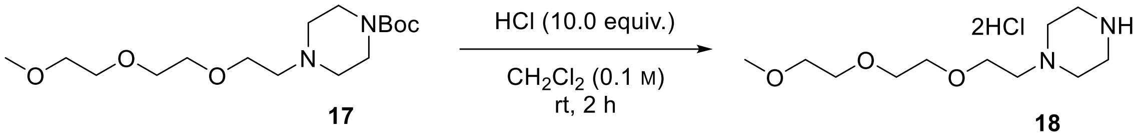

HCl (4 M in 1,4-dioxane, 3.8 mL, 15.2 mmol, 10.0 equiv.) was added to a solution of compound **17** (0.50 g, 1.5 mmol, 1.0 equiv.) in CH_2Cl_2 (13 mL, 0.1 M) and the reaction mixture was stirred at room temperature for 2 h. The reaction mixture was then concentrated under reduced pressure and azeotroped with CH_2Cl_2 (3 × 25 mL). The brown oil obtained (0.62 g) was used immediately in the subsequent reaction without characterisation.

6-(Formylpyridin-2-yl)methyl methanesulfonate (19)

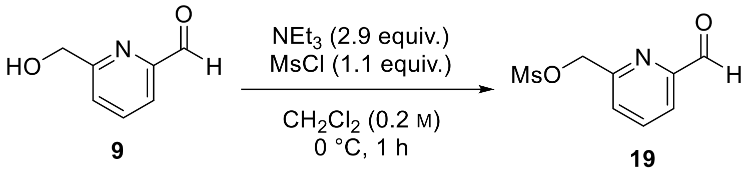

Methanesulfonyl chloride (0.06 mL, 803 µmol, 1.1 equiv.) was added dropwise to a solution of triethylamine (0.30 mL, 2.2 mmol, 2.9 equiv.) and compound **9** (0.10 g, 730 µmol, 1.0 equiv.) in anhydrous CH_2Cl_2 (4 mL, 0.2 M) under nitrogen at 0 °C, and stirred at 0 °C for 1 h. The reaction mixture was then quenched with saturated aqueous $NaHCO_3$ solution (10 mL) and the aqueous extracted with CH_2Cl_2 (3 × 30 mL). The combined organics were dried over Na_2SO_4 and concentrated under reduced pressure. The brown oil obtained (0.14 g) was used immediately in the subsequent reaction without characterisation.

6-((4-(2-(2-(2-Methoxyethoxy)ethoxy)ethyl)piperazin-1-yl)methyl)picolinaldehyde (3)

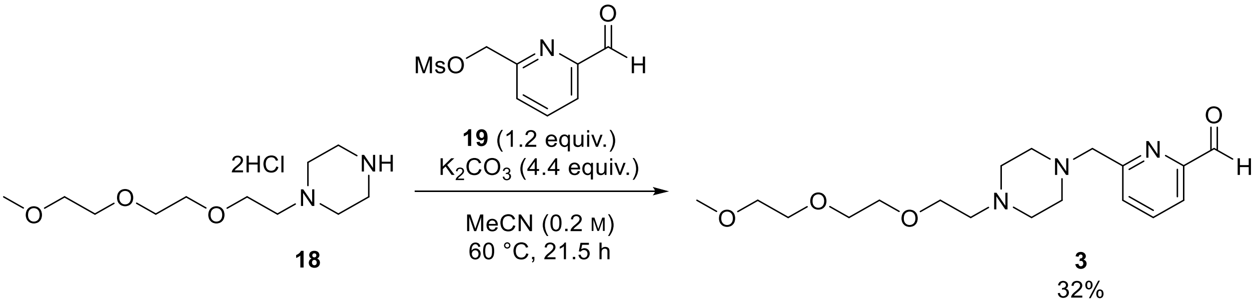

A solution of compound **19** (0.27 g, 900 µmol, 1.2 equiv.) in acetonitrile (4 mL, 0.2 M) was added to compound **18** (0.16 g, 750 µmol, 1.0 equiv.), and K_2CO_3 (0.45 g, 3.3 mmol, 4.4 equiv.) was then added to the reaction mixture. The reaction was stirred at 60 °C for 21.5 h and, after cooling to room temperature, the solvent was removed under reduced pressure. CH_2Cl_2 (30 mL) and saturated aqueous $NaHCO_3$ solution (25 mL) were added to the orange residue obtained. The aqueous layer was separated and extracted with CH_2Cl_2 (3 × 25 mL), and the combined organics were then dried over Na_2SO_4, filtered, and concentrated under reduced

pressure. The orange residue obtained was purified by flash column chromatography (10% MeOH:CH_2Cl_2, R_f 0.16), and pure fractions were concentrated under reduced pressure to afford the title compound (84 mg, 239 mmol, 32%) as a yellow oil. **^{1}H NMR** (400 MHz, $CDCl_3$) $\delta_{H:}$ 2.51-2.64 (10H, m, -NCH$_2$CH$_2$N-, -OCH$_2$-), 3.35 (3H, s, OMe), 3.50-3.54 (2H, m, -OCH$_2$-), 3.58-3.65 (8H, m, -OCH$_2$-), 3.75 (2H, s, -NCH$_2$Ar), 7.63-7.69 (1H, m, ArH5), 7.79-7.84 (2H, m, ArH3,4), 10.05 (1H, s, CHO); **^{13}C NMR** (101 MHz, $CDCl_3$) $\delta_{C:}$ 53.25 (-CH$_2$NCH$_2$Ar), 53.62 (-CH$_2$CH$_2$NCH$_2$Ar), 57.81 (-OCH$_2$-), 59.14 (OMe), 64.12 (-CH$_2$Ar), 68.97 (-OCH$_2$-), 70.44 (-OCH$_2$-), 70.61 (-OCH$_2$-), 70.70 (-OCH$_2$-), 72.02 (-CH$_2$OMe), 120.28 (ArC3), 127.54 (ArC5), 137.43 (ArC4), 152.43 (ArC2), 159.85 (ArC6), 193.81 (CHO); **ν_{max}** (ATR, cm^{-1}) 2874, 2812, 1712, 1591, 1456, 1352, 1303, 1255, 1200, 1105, 1013, 991, 936, 909, 833, 783, 734, 664, 619; **HRMS** (ESI$^+$) $C_{18}H_{30}N_3O_4$ [M+H]$^+$ found 352.2228, calculated 352.2231.

(d) TA4C ***4***

1-Azido-2-(2-(2-methoxyethoxy)ethoxy)ethane (20)

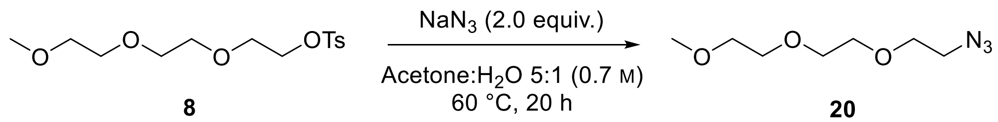

8 → **20** 91%

Sodium azide (0.54 g, 8.3 mmol, 2.0 equiv.) was added to a solution of compound **8** (1.33 g, 4.2 mmol, 1.0 equiv.) in acetone:water (5:1, 6 mL, 0.7 M), and the reaction mixture was stirred at 60 °C for 20 h. After cooling to room temperature, the acetone was removed under reduced pressure. The remaining solution was diluted with water (20 mL) and extracted with diethyl ether (4 × 20 mL). The combined organics were dried over $MgSO_4$, filtered, and concentrated under reduced pressure to afford the title compound as a pink oil (0.72 g, 3.8 mmol, 91%) with spectroscopic data in accordance with the literature.[8] **^{1}H NMR** (300 MHz, $CDCl_3$) $\delta_{H:}$ 3.31-3.40 (5H, m, OMe & -CH$_2$N$_3$), 3.49-3.55 (2H, m, -CH$_2$CH$_2$N$_3$), 3.59-3.69 (8H, m, -OCH$_2$-).

1-(2-(2-(2-Methoxyethoxy)ethoxy)ethyl)-1*H*-1,2,3-triazole-4-carbaldehyde (4)

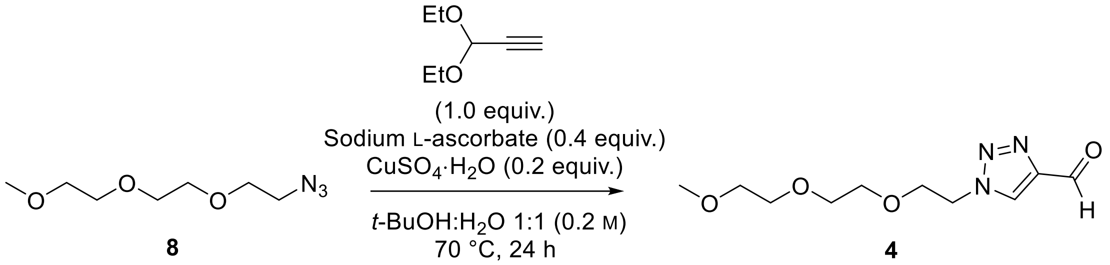

8 91% → **4** 61%

Azide **8** (0.10 g, 530 µmol, 1.0 equiv.) was dissolved in a mixture of *t*-BuOH:water (1:1, 2.7 mL, 0.2 M), and the solution was degassed by N_2 bubbling for 25 min. $CuSO_4 \cdot 5H_2O$ (33 mg, 105 µmol, 0.2 equiv.), sodium L-ascorbate (43 mg, 210 µmol, 0.4 equiv.) and 3,3-diethoxyprop-1-yne (75 µL, 530 µmol, 1.0 equiv.) were then

added under nitrogen and the reaction mixture was stirred at 70 °C for 24 h. After cooling to room temperature, the reaction was diluted with EtOAc (30 mL) and the organics washed with brine (10 mL). The aqueous layer was extracted with EtOAc (3 × 25 mL), and the combined organics were dried over $MgSO_4$, filtered, and concentrated under reduced pressure. The residue obtained was purified by flash column chromatography (EtOAc, R_f 0.27), and pure fractions were concentrated under reduced pressure to afford the title compound (79 mg, 325 µmol, 61%) as a colourless oil. **^{1}H NMR** (300 MHz, $CDCl_3$) $\delta_{H:}$ 3.35 (3H, s, OMe), 3.48-3.69 (8H, m, MeOC$\underline{H}_2$C$\underline{H}_2$OC$\underline{H}_2$C$\underline{H}_2$-), 3.88 (2H, t, J = 4.9 Hz, ArCH$_2$C$\underline{H}_2$-), 4.60 (2H, t, J = 4.9 Hz, ArC$\underline{H}_2$-), 8.37 (1H, s, Ar$\underline{H}$5), 10.12 (1H, s, C$\underline{H}$O); **^{13}C NMR** (75 MHz, $CDCl_3$) $\delta_{C:}$ 50.53 (-$\underline{C}$H$_2$Ar), 58.96 (OMe), 68.83 (-$\underline{C}$H$_2$CH$_2$Ar), 70.42 (-$\underline{C}$H$_2$), 70.50 (-$\underline{C}$H$_2$), 70.53 (-$\underline{C}$H$_2$), 71.84 (-$\underline{C}$H$_2$), 126.89 (Ar$\underline{C}$5), 147.75 (Ar$\underline{C}$4), 185.00 ($\underline{C}$HO); **ν_{max}** (ATR, cm^{-1}) 3128, 2872, 1695, 1531, 1467, 1352, 1285, 1245, 1183, 1101, 1041, 930, 848, 780, 707, 665, 642, 523, 474; **HRMS** (ESI$^+$) $C_{10}H_{17}N_3NaO_4$ [M+Na]$^+$ found 266.1113, calculated 266.1111.

(e) *2-EBA* ***5***

4-hydroxy-5-methoxy-2-((trimethylsilyl)ethynyl)benzaldehyde (21)

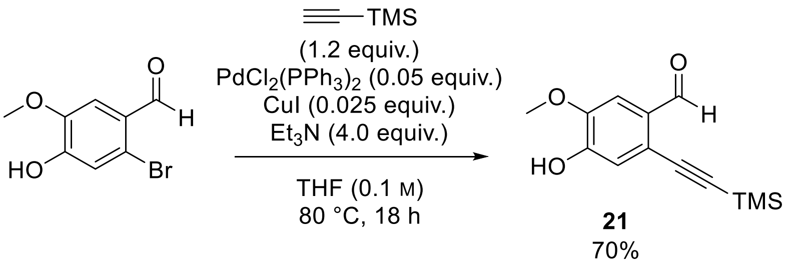

A solution of triethylamine (2.8 mL, 20.1 mmol, 4.0 equiv.) and 2-bromo-4-hydroxy-5-methoxybenzaldehyde (1.16 g, 5.0 mmol, 1.0 equiv.) in anhydrous THF (50 mL, 0.1 M) was added to a mixture of bis(triphenylphosphine)palladium (II) dichloride (175 mg, 0.2 mmol, 0.05 equiv.) and copper (I) iodide (28 mg, 0.1 mmol, 0.025 equiv.) under nitrogen. Ethynyltrimethylsilane (0.83 mL, 6.0 mmol, 1.2 equiv.) was then added and the reaction mixture was stirred at 80 °C for 18 h. After cooling to room temperature, the mixture was filtered through Celite and the filtrate concentrated under reduced pressure. The residue obtained was purified by flash column chromatography (30% EtOAc:Petrol, R_f 0.46), and pure fractions were concentrated under reduced pressure to afford the title compound as a cream-coloured solid (0.88 g, 3.5 mmol, 70%). **^{1}H NMR** (400 MHz, $CDCl_3$) $\delta_{H:}$ 0.26 (9H, s, TMS), 3.95 (3H, s, OMe), 6.27 (1H, s, ArO$\underline{H}$), 7.07 (1H, s, Ar$\underline{H}$3), 7.38 (1H, s, Ar$\underline{H}$6), 10.36 (1H, s, ArC$\underline{H}$O); **^{13}C NMR** (101 MHz, $CDCl_3$) $\delta_{C:}$ -0.07 (TMS), 56.33 (OMe), 99.91 (Me$_3$SiC$\underline{C}$Ar), 100.90 (Me$_3$Si$\underline{C}$CAr), 108.04 (Ar$\underline{C}$6), 118.68 (Ar$\underline{C}$3), 122.23 (Ar$\underline{C}$2), 130.37 (Ar$\underline{C}$1), 147.50 (Ar$\underline{C}$5), 150.91 (Ar$\underline{C}$4), 190.88 (Ar$\underline{C}$HO); **ν_{max}** (ATR, cm^{-1}) 3144, 2959, 2153, 1662, 1603, 1560, 1506, 1470, 1434, 1396, 1354, 1293, 1252, 1212, 1171, 1121, 1014, 901, 877, 842, 757, 734, 718, 591, 487; **mp** 129-132 °C; **HRMS** (ESI$^+$) $C_{13}H_{16}NaO_3Si$ [M+Na]$^+$ found 271.0769, calculated 271.0761.

2-Ethynyl-5-methoxy-4-(2-(2-(2-methoxyethoxy)ethoxy)ethoxy)benzaldehyde (5)

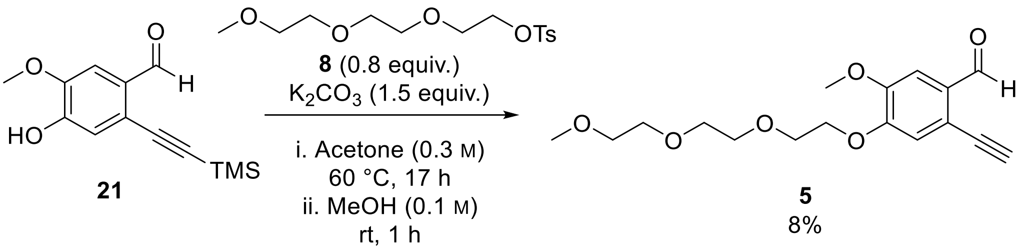

A solution of compound **8** (0.44 g, 1.4 mmol, 0.8 equiv.) in acetone (1 mL) was added to a mixture of K_2CO_3 (0.34 g, 2.5 mmol, 1.5 equiv.) and compound **21** (0.40 g, 1.6 mmol, 1.0 equiv.) in acetone (4 mL, 0.3 м), and the reaction mixture was stirred at 60 °C for 17 h. After cooling to room temperature, the reaction mixture was concentrated under reduced pressure and the residue obtained was dissolved in methanol (9.7 mL, 0.1 м) and stirred at room temperature for 1 h. The reaction mixture was then concentrated under reduced pressure, and saturated aqueous $NaHCO_3$ solution (20 mL) was added. The aqueous was extracted with CH_2Cl_2 (3 × 60 mL), and the combined organics were washed with saturated aqueous $NaHCO_3$ solution (50 mL), dried over $MgSO_4$, filtered, and concentrated under reduced pressure. The resulting residue was purified by flash column chromatography (66% EtOAc:Petrol, R_f 0.21), and pure fractions were concentrated under reduced pressure to afford the title compound as an orange oil (37 mg, 0.11 mmol, 8%). **^{1}H NMR** (300 MHz, CD_3OD) δ_H: 3.34 (3H, s, -CH$_2$O<u>Me</u>), 3.50-3.54 (2H, m, -OC<u>H</u>$_2$), 3.61-3.63 (2H, m, -OC<u>H</u>$_2$), 3.63-3.67 (2H, m, -OC<u>H</u>$_2$), 3.68-3.74 (2H, m, -OC<u>H</u>$_2$), 3.84-3.91 (2H, m, -OC<u>H</u>$_2$), 3.89 (3H, s, ArO<u>Me</u>), 3.91 (1H, s, ArCC<u>H</u>), 4.21-4.28 (2H, m, ArOC<u>H</u>$_2$), 7.15 (1H, s, Ar<u>H</u>3), 7.37 (1H, s, Ar<u>H</u>6), 10.32 (1H, s, C<u>H</u>O); **^{13}C NMR** (75 MHz, CD_3OD) δ_C: 56.46 (ArO<u>Me</u>), 59.07 (-CH$_2$O<u>Me</u>), 70.05 (-O<u>C</u>H$_2$), 70.48 (-O<u>C</u>H$_2$), 71.38 (-O<u>C</u>H$_2$), 71.58 (-O<u>C</u>H$_2$), 71.85 (-O<u>C</u>H$_2$), 72.96 (-O<u>C</u>H$_2$), 79.91 (ArC<u>C</u>H), 84.80 (Ar<u>C</u>CH), 109.45 (Ar<u>C</u>6), 117.66 (Ar<u>C</u>3), 121.61 (Ar<u>C</u>2), 131.99 (Ar<u>C</u>1), 151.80 (Ar<u>C</u>5), 154.75 (Ar<u>C</u>4), 191.34 (Ar<u>C</u>HO); **v_{max}** (ATR, cm^{-1}) 3251, 2874, 1682, 1590, 1566, 1507, 1457, 1397, 1347, 1276, 1219, 1096, 1054, 1013, 948, 874, 856, 759, 684, 645, 588, 555, 475; **HRMS** (ESI$^+$) $C_{17}H_{23}O_6$ [M+H]$^+$ found 323.1493, calculated 323.1489.

(f) *Ox* ***6***

Methyl (*S*)-2-thioxooxazolidine-4-carboxylate (22)

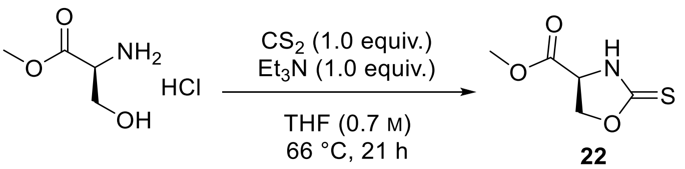

Triethylamine (0.9 mL, 6.5 mmol, 1.0 equiv.) was added to a solution of L-serine methyl ester hydrochloride (1.02 g, 6.5 mmol, 1.0 equiv.) in THF (9.2 mL, 0.7 м) at 0 °C. Carbon disulfide (0.39 mL, 6.5 mmol, 1.0 equiv.) was then added and the reaction mixture was warmed to 66 °C and stirred for 21 h [WARNING: Stench]. After cooling to room temperature, the reaction mixture was diluted with CH_2Cl_2 (50 mL) and the organics washed with saturated aqueous $NaHCO_3$ solution (2 × 20 mL), dried over $MgSO_4$, filtered, and concentrated under reduced pressure. The resulting residue was purified by flash column chromatography (50%

EtOAc:Petrol, R_f 0.50 in 66% EtOAc:Petrol), and pure fractions were concentrated under reduced pressure to afford a yellow oil (0.61 g), which was used immediately in the next step without characterisation.

Methyl (*S*)-2-(methylthio)-4,5-dihydrooxazole-4-carboxylate (23)

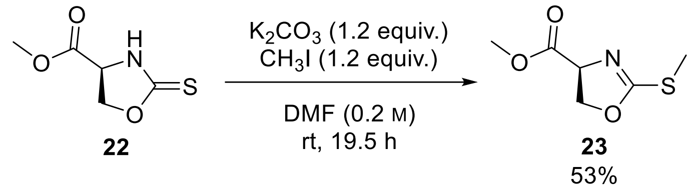

Methyl iodide (283 µL, 4.5 mmol, 1.2 equiv.) was added dropwise to a mixture of compound **22** (0.61 g, 3.8 mmol, 1.0 equiv.) and K_2CO_3 (0.63 g, 4.6 mmol, 1.2 equiv.) in DMF (20 mL, 0.2 M), and the reaction mixture was stirred at room temperature for 19.5 h. Brine (30 mL) was then added, and the aqueous was extracted with EtOAc (3 × 50 mL). The combined organics were washed with brine (3 × 50 mL), dried over $MgSO_4$, filtered, and concentrated under reduced pressure. The residue was purified by flash column chromatography (33% EtOAc:Petrol, R_f 0.48 in 66% EtOAc:Petrol), and pure fractions were concentrated under reduced pressure to afford the title compound (0.35 g, 2.0 mmol, 53%) as a yellow oil, with spectroscopic data in accordance with the literature.[9] **^{1}H NMR** (300 MHz, $CDCl_3$) δ_H 2.49 (3H, s, SMe), 3.79 (3H, s, OMe), 4.47-4.66 (2H, m, -NCHC$\underline{H}_2$), 4.71-4.83 (1H, m, -NC$\underline{H}$).

(*R*)-(2-(methylthio)-4,5-dihydrooxazol-4-yl)methanol (24)

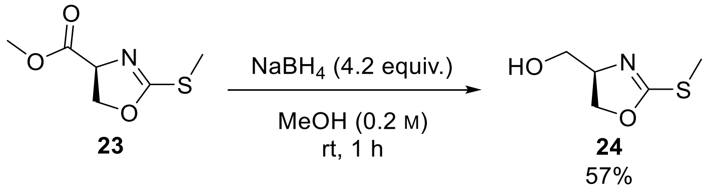

Sodium borohydride (0.18 g, 4.7 mmol, 4.2 equiv.) was added in small portions over 20 min to a solution of compound **23** (0.20 g, 1.1 mmol, 1.0 equiv.) in anhydrous methanol (5.5 mL, 0.2 M) at 0 °C under nitrogen. The reaction mixture was warmed to room temperature and stirred for 1 h, then quenched through dropwise addition of water (15 mL). The aqueous was extracted with ethyl acetate (3 × 45 mL) and the combined organics were dried over $MgSO_4$, filtered, and concentrated under reduced pressure to afford the target compound (94 mg, 0.6 mmol, 57%) as a pale yellow oil with spectroscopic data in accordance with the literature.[9] **^{1}H NMR** (300 MHz, $CDCl_3$) δ_H 2.47 (3H, s, SMe), 3.50-3.65 (1H, m, -C$\underline{H}_2$OH), 3.75-3.94 (1H, m, -C$\underline{H}_2$OH), 4.19-4.37 (2H, m, -NCHC$\underline{H}_2$OC-), 4.37-4.52 (1H, m, -NC$\underline{H}$).

(*R*)-4-(2,5,8,11-tetraoxadodecyl)-2-(methylthio)-4,5-dihydrooxazole (6)

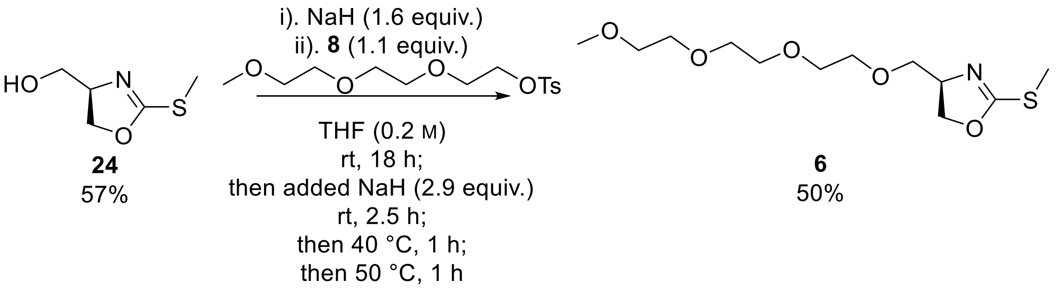

Sodium hydride (60% dispersion in mineral oil, 38 mg, 1.0 mmol, 1.6 equiv.) was added in small portions over 10 min to a solution of compound **24** (86 mg, 585 µmol, 1.0 equiv.) in anhydrous THF (3 mL, 0.2 ᴍ) under nitrogen. After 5 min, a solution of compound **8** (205 mg, 644 µmol, 1.1 equiv.) in THF (0.5 mL) was added and the reaction mixture was stirred at room temperature for 18 h. At this point TLC analysis demonstrated the reaction was incomplete, and so a further portion of sodium hydride (69 mg, 1.7 mmol, 2.9 equiv.) was added and the reaction mixture was stirred at room temperature for a further 2.5 h, then at 40 °C for 1 h, and 50 °C for 1 h. The reaction mixture was allowed to cool to room temperature, then quenched through dropwise addition of water (5 mL) and extracted with ethyl acetate (6 × 20 mL). The combined organics were dried over $MgSO_4$, filtered, and concentrated under reduced pressure. The orange oil obtained was purified by flash column chromatography (33-100% EtOAc:Petrol, R_f 0.09 in 33% EtOAc:Petrol), and pure fractions were concentrated under reduced pressure to afford the title compound (85 mg, 0.29 mmol, 50%) as a yellow oil. **^{1}H NMR** (300 MHz, $CDCl_3$) δ_H 2.43 (3H, s, SMe), 3.35 (3H, s, OMe), 3.34-3.44 (1H, m, -C$\underline{H}_2$CHCH_2OCSMe-), 3.49-3.55 (2H, m, -C$\underline{H}_2$-), 3.57-3.65 (10H, m, -C$\underline{H}_2$-), 3.65-3.72 (1H, m, -C$\underline{H}_2$CHN-), 4.19-4.35 (2H, m, -C$\underline{H}_2$OCSMe), 4.35-4.45 (1H, m, -NC$\underline{H}$); **^{13}C NMR** (75 MHz, $CDCl_3$) δ_C 14.51 (SMe), 59.09 (OMe), 66.23 (-N$\underline{C}$H), 70.54 (-$\underline{C}H_2$-), 70.60 (-$\underline{C}H_2$-), 70.70 (2 × -$\underline{C}H_2$-), 70.97 (-$\underline{C}H_2$-), 72.01 (-$\underline{C}H_2$OMe), 72.67 (-$\underline{C}H_2$OCSMe), 73.22 (-$\underline{C}H_2$CHN-), 167.62 (-$\underline{C}$SMe); **ν_{max}** (ATR, cm^{-1}) 3433, 2872, 1759, 1670, 1604, 1455, 1349, 1329, 1287, 1250, 1199, 1100, 954, 927, 881, 851, 720, 648, 529; **HRMS** (ESI$^+$) $C_{12}H_{24}NO_5S$ $[M+H]^+$ found 294.1367, calculated 294.1370.

(g) *BA* ***7***

4-(2-(2-(2-Methoxyethoxy)ethoxy)ethoxy)benzaldehyde (7)

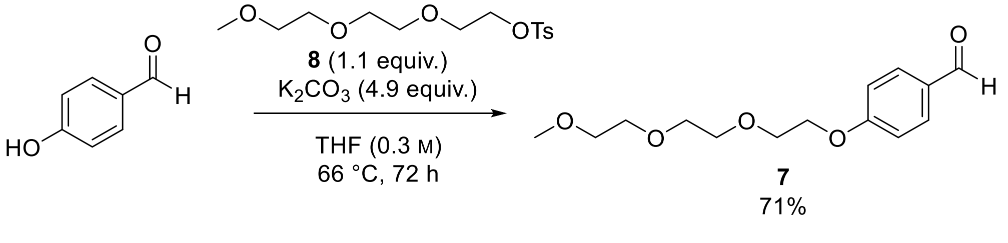

A solution of compound **8** (280 mg, 880 µmol, 1.1 equiv.) in THF (0.5 mL) was added to a mixture of K_2CO_3 (0.29 g, 2.1 mmol, 2.5 equiv.) and 4-hydroxybenzaldehyde (100 mg, 820 µmol, 1.0 equiv.) in THF (2.4 mL, 0.3 ᴍ), and the reaction stirred at 66 °C for 24 h. At this point TLC analysis demonstrated the reaction was incomplete, and so a further portion of K_2CO_3 (0.28 g, 2.0 mmol, 2.4 equiv.) was added and the reaction

mixture was stirred at 66 °C for 48 h. The reaction mixture was allowed to cool to room temperature, then water (20 mL) was added and the aqueous was extracted with CH_2Cl_2 (3 × 20 mL). The combined organics were dried over $MgSO_4$, filtered, and concentrated under reduced pressure. The brown oil obtained was purified by flash column chromatography (33-50% EtOAc:Petrol, R_f 0.18 in 33% EtOAc:Petrol), and pure fractions were concentrated under reduced pressure to afford the title compound (0.16 g, 0.6 mmol, 71%) as a pale-yellow oil with spectroscopic data in accordance with the literature.[10] **^{1}H NMR** (300 MHz, $CDCl_3$) δ_H 3.37 (3H, s, OMe), 3.50-3.57 (2H, m, -C$\underline{H}_2$-), 3.60-3.70 (4H, m, -C$\underline{H}_2$-), 3.71-3.78 (2H, m, -C$\underline{H}_2$-), 3.83-3.93 (2H, t, *J* = 4.9 Hz, -ArOCH$_2$C$\underline{H}_2$-), 4.21 (2H, t, *J* = 4.9 Hz, ArOC$\underline{H}_2$), 7.02 (2H, d, *J* = 8.7 Hz, Ar$\underline{H}$3), 7.81 (2H, d, *J* = 8.7 Hz, Ar$\underline{H}$2), 9.87 (1H, s, C$\underline{H}$O).

2. Protein expression

Mutagenesis: Plasmid CjX183D_pCW codes for the expression of the c-type cytochrome CjX183-D and ampicillin resistance within a pCW-LIC vector. Site-directed mutagenesis was undertaken to install a lysine at residue at position 51 with a QuickChange Site-Directed Mutagenesis Kit (Agilent Technologies), following the manufacturer's instructions and the following primers:

Oligo Name	Sequence (5' → 3')	Length (bp)	GC%	Tm (Â °C)
R51Kforward	TGCACGGTCCTGGGTATCCTTGCTGTGGCGATAGAGATC	39	56.4	69.2
R51Kreverse	GATCTCTATCGCCACAGCAAGGATACCCAGGACCGTGCA	39	56.4	69.2

The mutagenesis mixtures were transformed into XL1-Blue Supercompetent cells and the cells plated onto LB agar containing ampicillin (100 µg/mL), and incubated overnight at 37 °C, as detailed in the manufacturer's instructions. Colonies were selected and plasmids harvested using a QIAprep Spin Miniprep Kit. Sequencing of the CjX183-D R51K_pCW construct was conducted using LightRun Tubes (Eurofins Genomics), using the following primers:

Oligo Name	Sequence (5' → 3')	Length (bp)	GC%	Tm (Â °C)
CjX183D_pCW_SEQ_FOR	GGAAACAGGATCAGCTTAC	19	47.4	50.4
CjX183D_pCW_SEQ_REV	CCTTTCGTCTTCAAGCAGAT	20	45.0	52.7

Co-transformation: Plasmid CjX183D_pCW or CjX183D-R51K_pCW (1 µL, 50 ng μL^{-1}), and the PEC86 plasmid (1 µL, 50 ng μL^{-1}) required for cytochrome maturation were added to BL21 DE3 chemically competent cells (50 µL) in a sterile 15 mL centrifuge tube on ice, and the cells left on ice for 30 min. The cells were then heat shocked at 42 °C for 45 seconds, before being placed back on ice for 2 min. Room temperature SOC media (200 µL) was then added and the cells incubated at 37 °C, 200 rpm, for 1 h. After this time, cells were plated onto LB agar containing ampicillin (100 µg/mL) and chloramphenicol (35 µg/mL), and incubated overnight at 37 °C. Individual colonies were then selected (successful co-transformation can result in colonies that appear to be a slightly red/orange colour) and transferred into 5 mL 2×YT media containing ampicillin (100 µg/mL) and chloramphenicol (35 µg/mL). The inoculated media was incubated at 37 °C, 180 rpm overnight. 2 mL of the overnight culture was then taken, the cells were pelleted by centrifugation (2 min, 1000

g), and 1.5 mL of the supernatant was removed. The cells were resuspended in the remaining 500 µL, and 50% glycerol (750 µL) was added, pipetting the solution several times to ensure mixing. The resulting solution was flash frozen using liquid N_2 and the resultant glycerol stock stored at -80 °C.

Protein expression: The glycerol stocks described above were plated onto ampicillin- and chloramphenicol-containing LB agar and grown at 37 °C overnight. A single colony was then selected and grown in 5 mL of 2×YT, containing the appropriate antibiotics, at 37 °C and 200 rpm for ~ 8 hrs. This culture was then transferred into 100 mL 2×YT, containing the appropriate antibiotics, and incubated overnight at 37 °C and 200 rpm. After this time, as a preliminary test to confirm that cells contained both the desired plasmids, a portion of the cells were pelleted via centrifugation, and were found to be red/pink in colour.

10 mL of the overnight culture was then used to inoculate 1000 mL of 2×YT, containing the appropriate antibiotics, in a non-baffled conical flask, and the flask was incubated at 37 °C and 180 rpm until $OD_{600} \approx 0.6$. The culture was then cooled to 16 °C and shaken at 150 rpm before addition of a solution of isopropyl ß-D-1-thiogalactopyranoside (IPTG, 1 M, 1 mL; Final concentration 1 mM). Cultures were then incubated at 16 °C and 150 rpm overnight.

Cells were harvested via centrifugation (4 °C, 20 min, 6000 g) and the supernatant removed. The cell pellet was resuspended in 50 mL of lysis buffer (20 mM Tris, 150 mM NaCl, 10% glycerol, pH 8.0), and a protease inhibitor tablet (Thermo Scientific) was added, along with a solution of benzonase (2.5 U µL^{-1}, 20 µL). The solution was sonicated in bursts of 30 seconds (with 30 second cooling times between bursts) on ice until centrifugation of samples of the cell solution showed the presence of nuclear debris (a black dot). The lysed cell solution was then subjected to centrifugation (4 °C, 30 min, 30750 g). The supernatant was isolated, and imidazole added to a final concentration of 30 mM.

The supernatant was then loaded on a HisTrap HP column (GE Healthcare) equilibrated with equilibration buffer (pH 8.0, 20 mM Tris, 200 mM NaCl, 30 mM imidazole) (**Figure S1**). After washing the loaded protein with equilibration buffer (pH 8.0, 20 mM Tris, 200 mM NaCl, 30 mM imidazole), the CjX183-D variant was eluted with a linear gradient of elution buffer (pH 8.0, 20 mM Tris, 200 mM NaCl, 300 mM imidazole) over 10 column volumes. The presence of CjX183-D holo-protein on the HisTrap HP column was clearly visible (**Figure S1c**). The CjX183-D variant-containing fractions were identified by 15% SDS-PAGE gel analysis (**Figure S top**, **Figure S top**) and dialysed overnight into storage buffer (pH 8.0, 20 mM Tris, 200 mM NaCl). The CjX183-D variant solution was then concentrated to 2 mL using 3 kDa molecular weight cut-off centrifugal protein concentrators and further purified using a HiLoad Superdex® S75 column (GE Healthcare) equilibrated with storage buffer (pH 8.0, 20 mM Tris, 200 mM NaCl) (**Figure S1d**). A 2000 µL injection loop was used and the column was run at a flow rate of 1 mL min^{-1}, collecting fractions after $\frac{1}{3}$ of a column volume had been passed. Fractions containing the purified CjX183-D variant were identified by 15% SDS-PAGE gel analysis (**Figure S bottom**, **Figure S2 bottom**, these fractions were also red, **Figure S1e**), concentrated using 3 kDa molecular weight cut-off centrifugal protein concentrators to the desired concentration, frozen using liquid N_2, aliquoted, and stored at -80 °C.

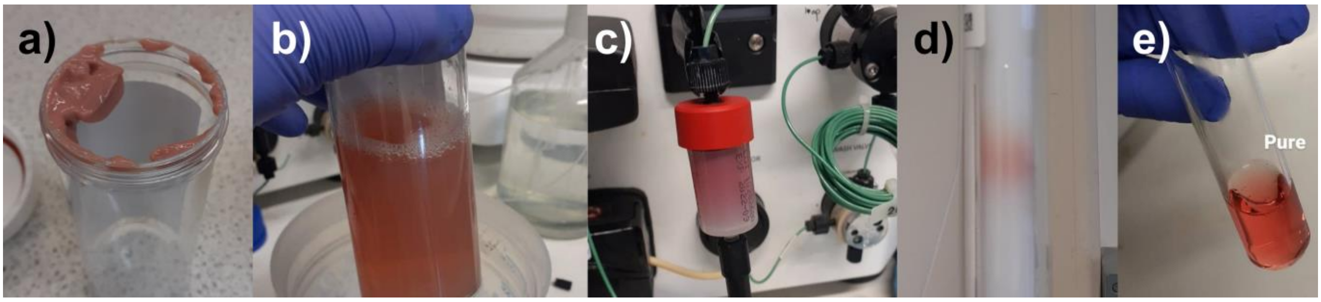

Figure S1. The appearance of pellets/solutions encountered throughout the preparation of CjX183-D variants. (***a***) The cell pellet of CjX183-D holo-protein containing cells; (***b***) The CjX183-D holo-protein containing supernatant of the cell lysate; (***c***) CjX183-D holo-protein being retained on a HisTrap HP column; (***d***) CjX183-D holo-protein travelling through a HiLoad Superdex® S75 column; (***e***) Purified CjX183-D holo protein.

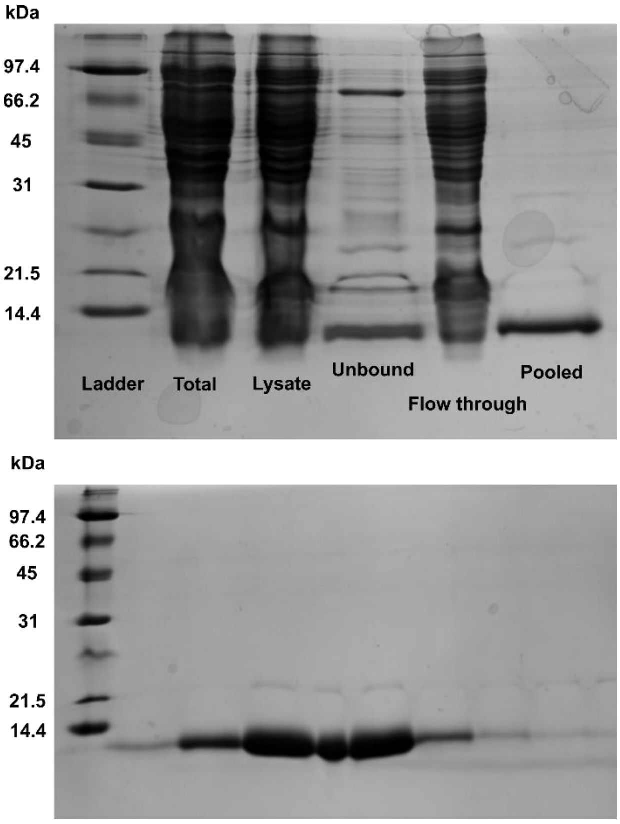

Figure S2. 15% SDS-PAGE gels illustrating the presence of WT CjX183-D. Top) 15% SDS-PAGE gel showing the purification of WT CjX183-D using Ni affinity chromatography. Bottom) 15% SDS-PAGE gel showing further purification of WT CjX183-D via size exclusion chromatography. CjX183-D has a theoretical molecular weight of 11.2 kDa, including the covalently attached c-type heme unit.

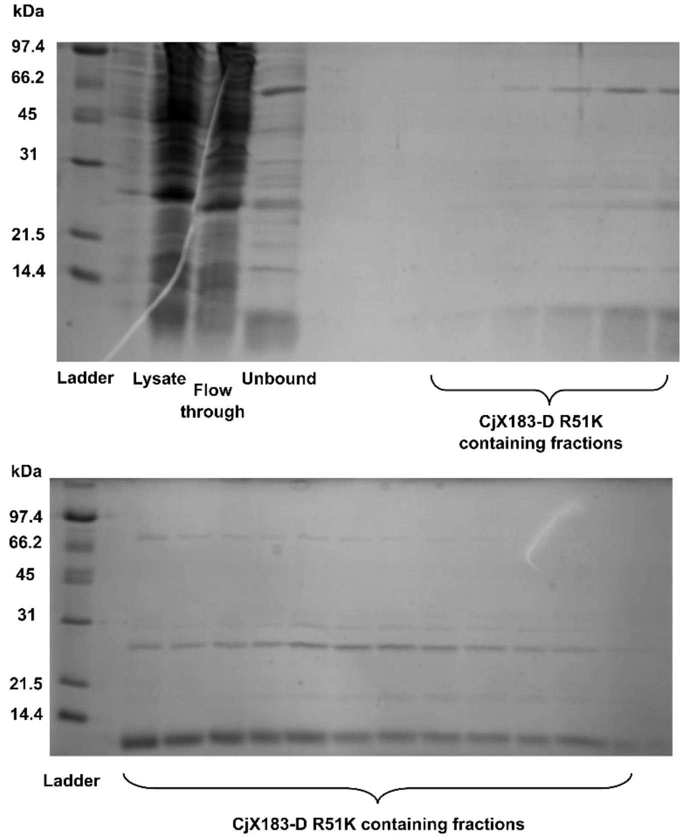

Figure S3. 15% SDS-PAGE gel showing the purification of CjX183-D R51K using Ni-affinity chromatography. CjX183-D R51K has a theoretical molecular weight of 11.2 kDa, including the covalently attached c-type heme unit.

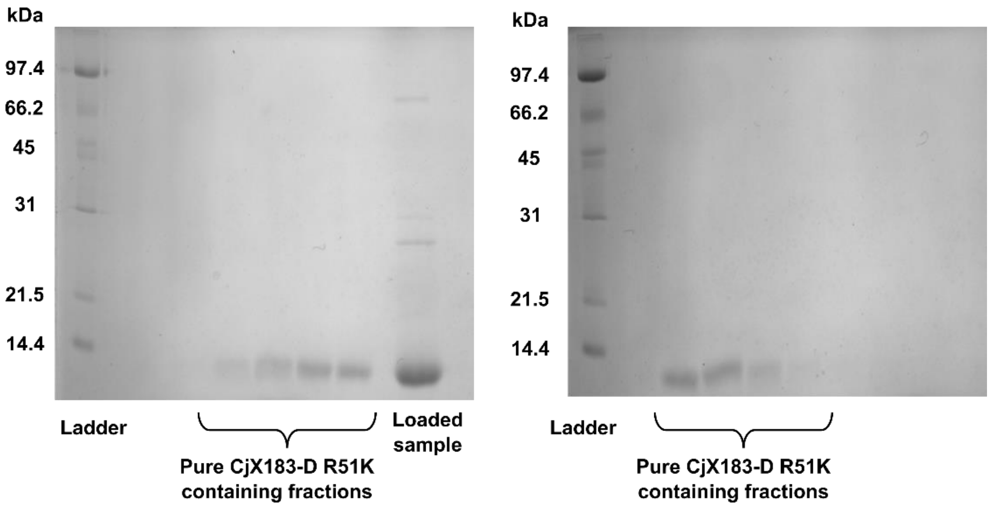

Figure S2. 15% SDS-PAGE gel showing the purification of CjX183-D R51K via size-exclusion chromatography. CjX183-D R51K has a theoretical molecular weight of 11.2 kDa, including the covalently attached c-type heme unit.

Protein Sequences:

CjX183-D

GYLVGDATRG ANLWNTQTCV ACHGVDGERN ASGTPALTPL NPNRDLYRHS RDTQDRALRD FISMWMPQGN EGSCTGQCAA DIEAFIRTWH HHHHH

Theoretical M_w (+heme): 11226 Da

CjX183-D R51K

GYLVGDATRG ANLWNTQTCV ACHGVDGERN ASGTPALTPL NPNRDLYRHS KDTQDRALRD FISMWMPQGN EGSCTGQCAA DIEAFIRTWH HHHHH

Theoretical M_w (+heme): 11198 Da

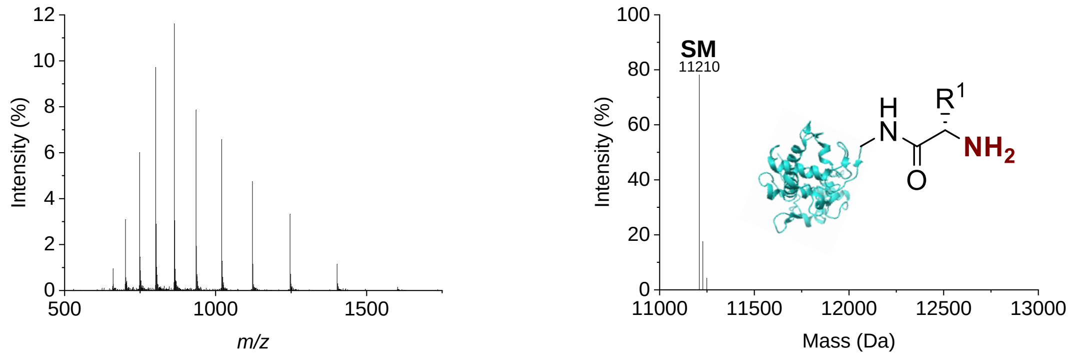

MS (ESI$^+$) [**SM**+H]$^+$ found 11210, calculated 11198.

Nb. In all experiments performed on CjX183-D, both wild type and mutants, the observed mass was ~ 12 Da higher than the calculated mass. Reasons for this difference from the theoretical mass are currently under investigation.

3. Protein modification

3.1 Validation of literature conditions and general procedures

2-PCAs 1-3

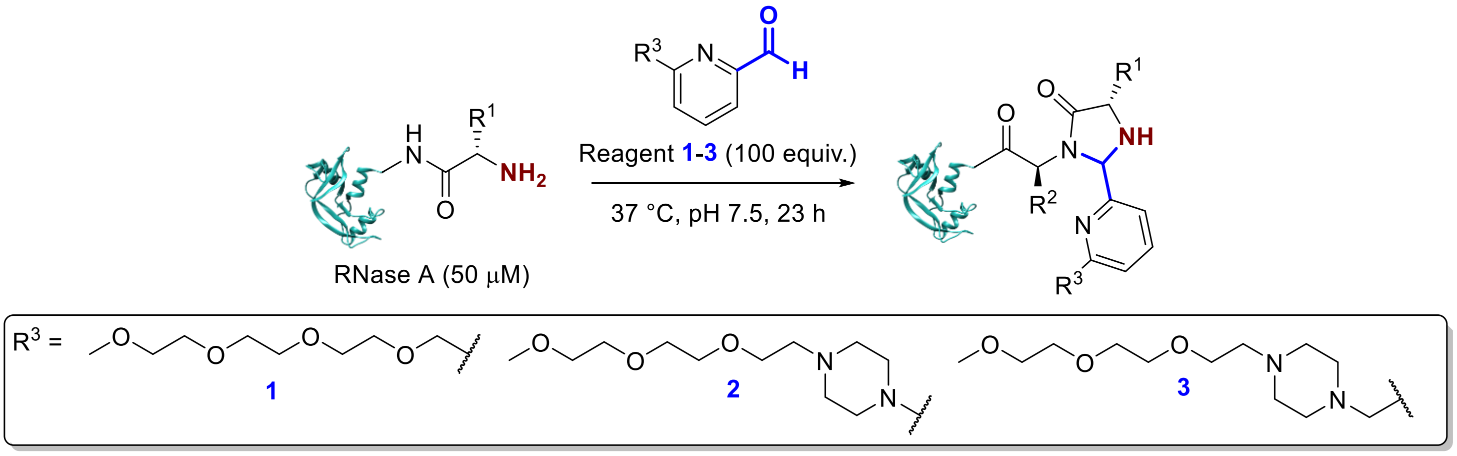

Procedure A: *Modification with 2-PCAs* ***1-3*** *under conditions reported by MacDonald et al.*[3] A stock solution of 2-PCA **1**, **2,** or **3** (50 µL, 10 mM, 500 nmol, 100 equiv., in 50 mM pH 7.5 sodium phosphate buffer) was added to a solution of RNase A (50 µL, 100 µM, 5 nmol, 1 equiv., in 50 mM pH 7.5 sodium phosphate buffer), and the mixture incubated at 37 °C for 23 h with agitation (1000 rpm). Conversion was determined by LC-MS analysis without purification.

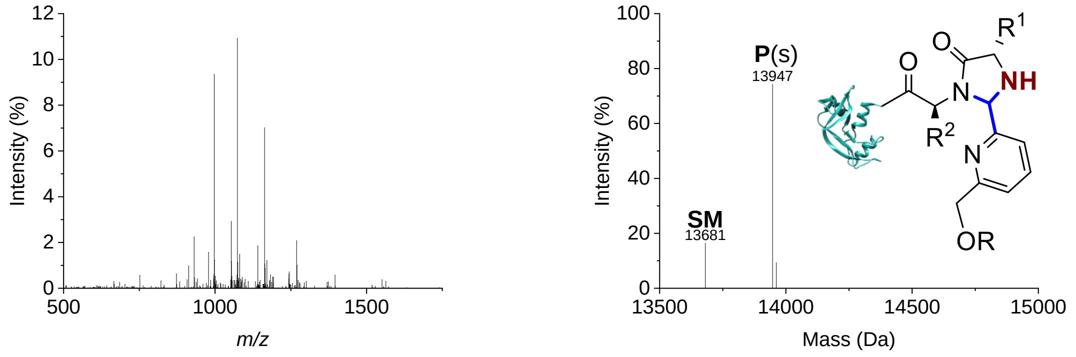

2-PCA **1***

84%

MS (ESI+) [**SM**+H]+ found 13681, calculated 13681;[1] [**P**(s)+H]+ found 13947, calculated 13946; [**P**(s)+H_2O+H]+ found 13962, calculated 13964.

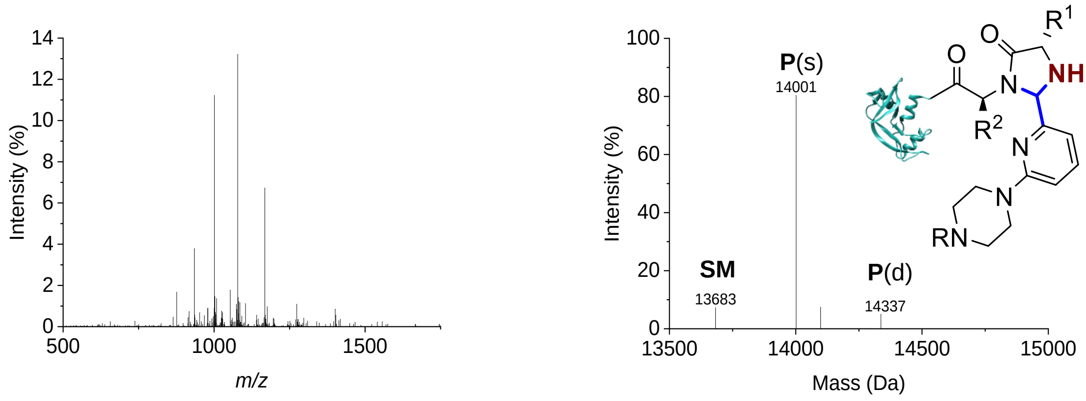

2-PCA **2***

93%

88(s):5(d)

MS (ESI+) [**SM**+H]+ found 13683, calculated 13681;[1] [**P**(s)+H]+ found 14001, calculated 14001; [**P**(s)+H_3PO_4+H]+ found 14099, calculated 14098; [**P**(d)+H_2O+H]+ found 14337, calculated 14338.

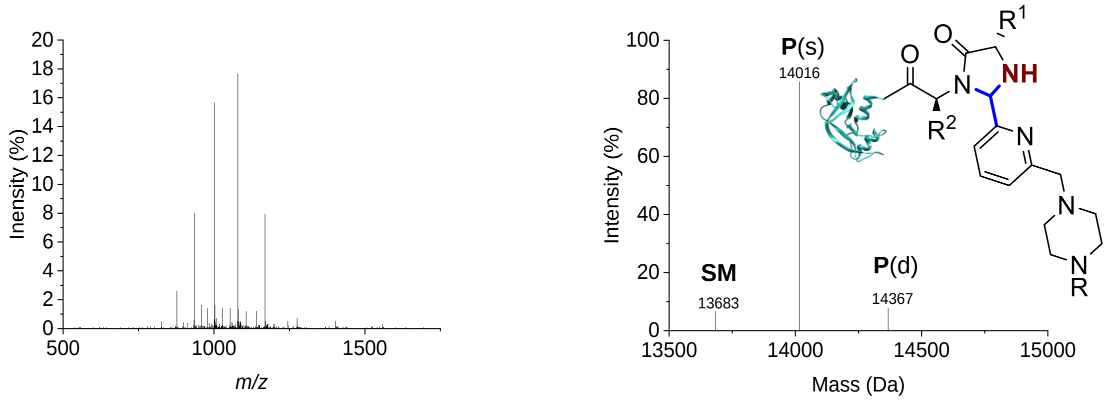

2-PCA **3***

94%

86(s):8(d)

MS (ESI+) [**SM**+H]+ found 13683, calculated 13681;[1] [**P**(s)+H]+ found 14016, calculated 14015; [**P**(d)+H_2O+H]+ found 14367, calculated 14366.

**Following LC-MS of each protein sample, water was injected into the LC column to remove residual protein; for samples marked (*), conversions were determined from MS traces of residual protein due to co-elution with excess small molecule.*

TA4C 4

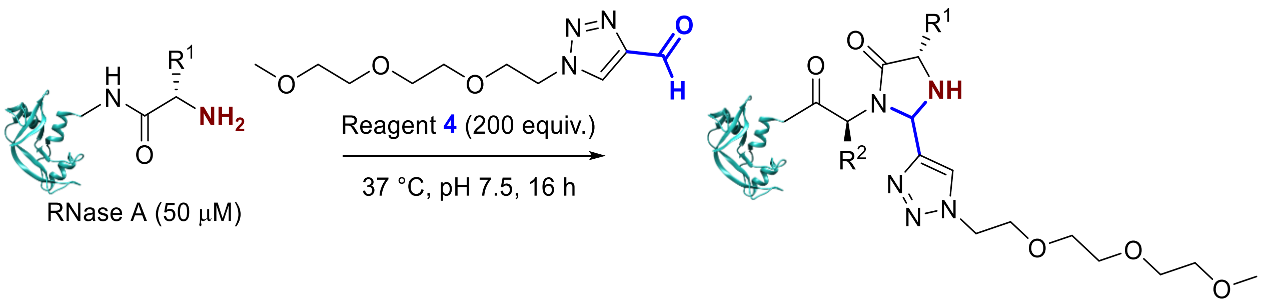

Procedure B: *Modification with TA4C **4** under conditions reported by Onoda et al.*[11] A stock solution of TA4C **4** (2.5 µL, 200 mM, 500 nmol, 166 equiv., in DMSO) was added to a solution of RNase A (47.5 µL, 52 µM, 3 nmol, 1 equiv., in 10 mM pH 7.5 potassium phosphate buffer), and the mixture incubated at 37 °C for 16 h with agitation (1000 rpm). Conversion was determined by LC-MS analysis without purification.

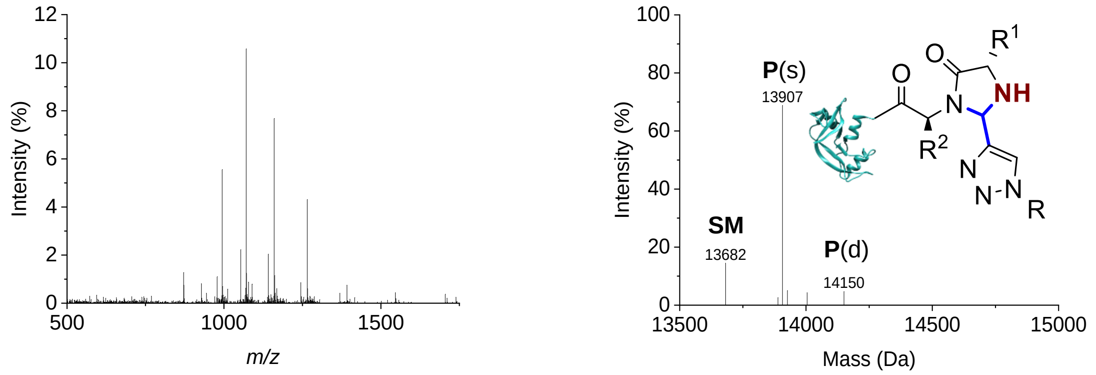

TA4C **4**

86%

81(s):5(d)

MS (ESI$^+$) [**SM**+H]$^+$ found 13682, calculated 13681; [**P**(s)–H_2O+H]$^+$ found 13889, calculated 13888; [**P**(s)+H]$^+$ found 13907, calculated 13906; [**P**(s)+H_2O+H]$^+$ found 13926, calculated 13924; [**P**(s)+H_3PO_4+H]$^+$ found 14004, calculated 14004; [**P**(d)+H_2O+H]$^+$ found 14150, calculated 14150.

2-EBA 5

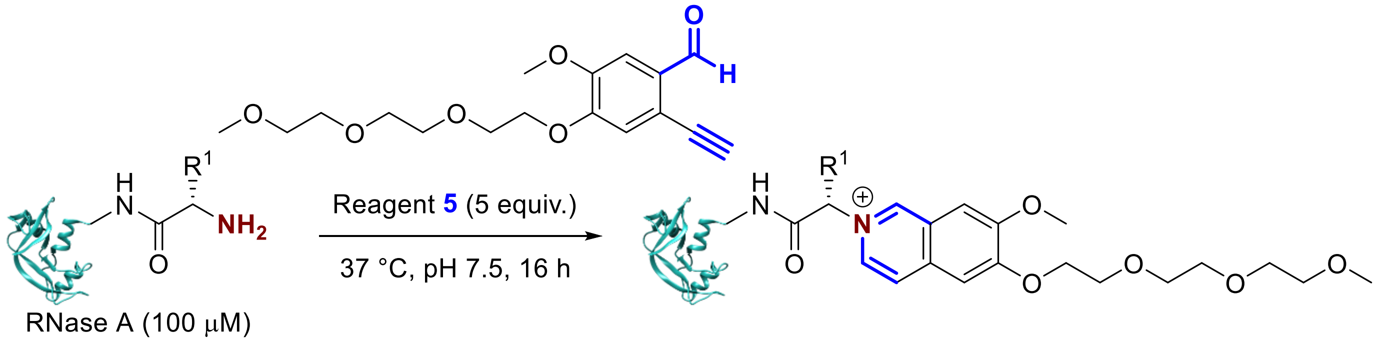

Procedure C: *Modification with 2-EBA **5** under conditions reported by Deng et al.*[12] A stock solution of 2-EBA **5** (15 µL, 5 mM, 75 nmol, 5 equiv., in DMSO) was added to a solution of RNase A (135 µL, 111 µM, 15 nmol, 1 equiv., in 50 mM pH 7.5 phosphate buffered saline), and the mixture incubated at 37 °C for 16 h with agitation (1000 rpm). Conversion was determined by LC-MS analysis without purification.

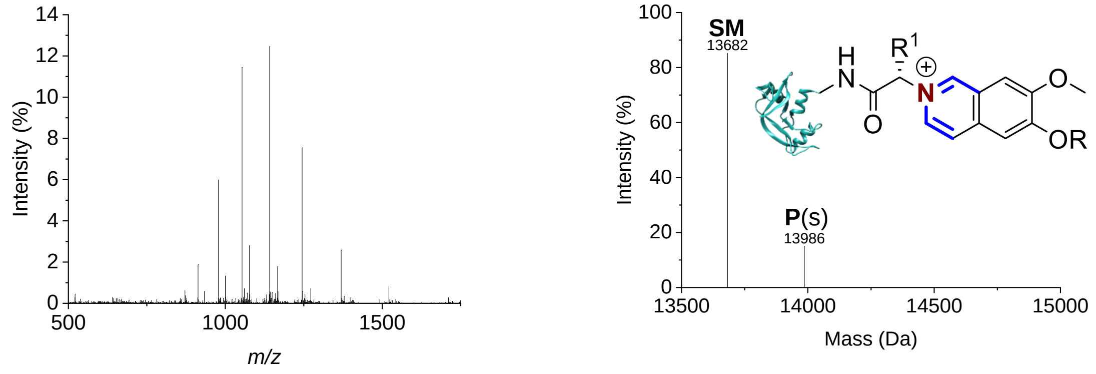

2-EBA **5**

15%

MS (ESI$^+$) [**SM**+H]$^+$ found 13682, calculated 13681; [**P**(s)+H]$^+$ found 13986, calculated 13985.

Ox 6

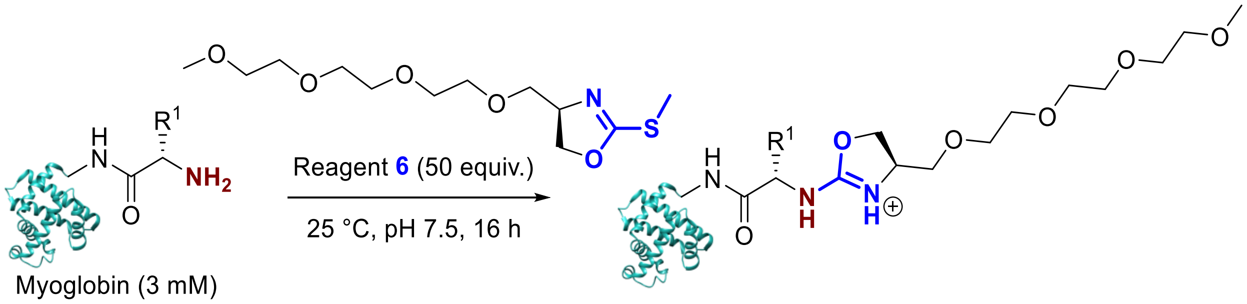

Procedure D: *Modification with Ox **6** under conditions reported by Raj et al.*[9] A stock solution of Ox **6** (15 µL, 300 mM, 4.5 µmol, 50 equiv., in 10 mM pH 7.5 sodium phosphate buffer) was added to a solution of myoglobin (15 µL, 6 mM, 90 nmol, 1 equiv., in 10 mM pH 7.5 sodium phosphate buffer), and the mixture incubated at 25 °C for 16 h with agitation (1000 rpm). Samples were diluted ×60 with water prior to analysis of conversion by LC-MS analysis without purification.

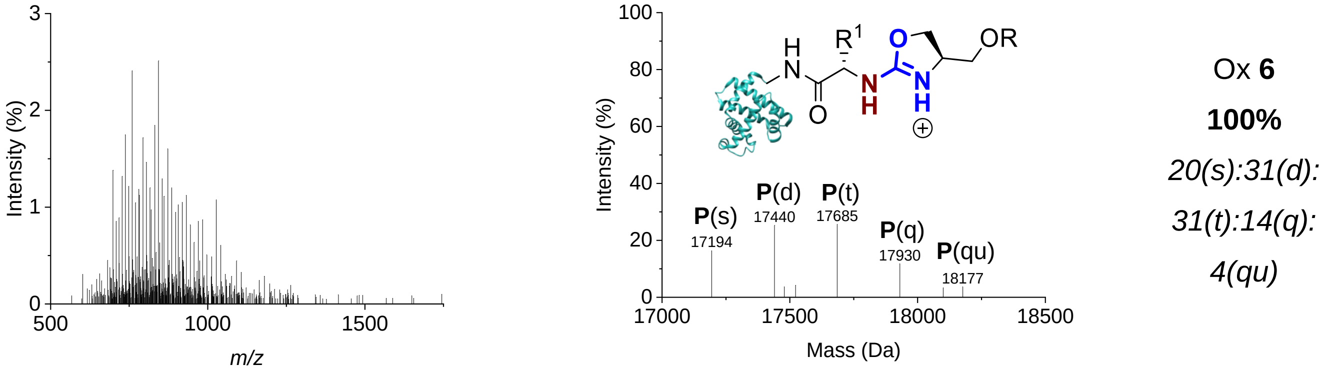

MS (ESI$^+$) [**P**(s)+H]$^+$ found 17194, calculated 17197; [**P**(d)+H]$^+$ found 17440, calculated 17442; [**P**(t)+H]$^+$ found 17685, calculated 17687; [**P**(q)+H]$^+$ found 17930, calculated 17932; [**P**(qu)+H]$^+$ found 18177, calculated 18177.

BA 7

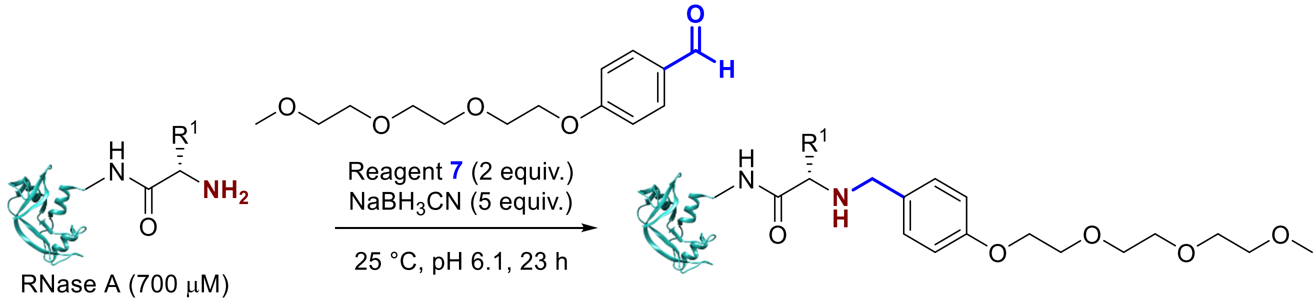

Procedure E: *Modification with BA **7** under conditions reported by Chen et al.*[13] Stock solutions of BA **7** (22.5 µL, 3 mM, 70 µmol, 2 equiv., in 25 mM pH 6.1 citrate buffer) and sodium cyanoborohydride (2.5 µL, 70 mM, 175 µmol, 5 equiv., in water) were sequentially added to a solution of RNase A (25 µL, 1.4 mM, 35 nmol, 1 equiv., in in 25 mM pH 6.1 citrate buffer), and the mixture incubated at 25 °C for 23 h with agitation (1000 rpm). Samples were diluted ×10 with 25 mM pH 6.1 citrate buffer prior to analysis of conversion by LC-MS analysis without purification.

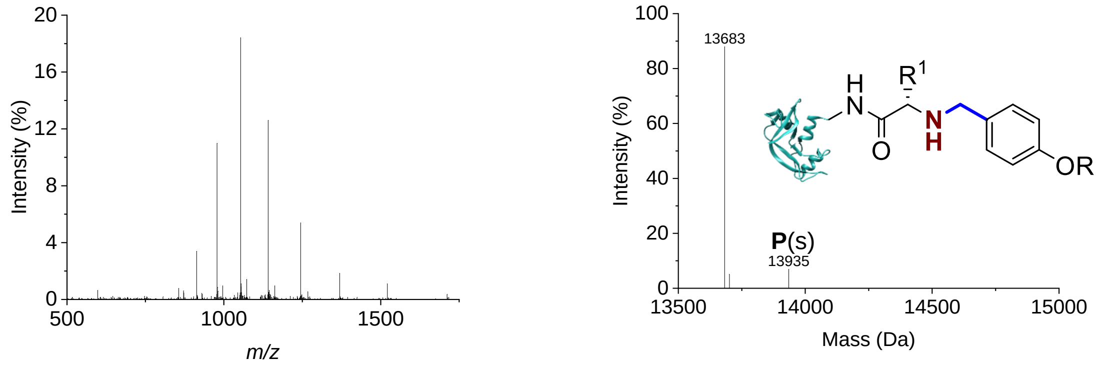

BA **7**

7%

MS (ESI$^+$) [**SM**+H]$^+$ found 13683, calculated 13681; [**SM**+H_2O+H]$^+$ found 13701, calculated 13699; [**P**(s)+H]$^+$ found 13935, calculated 13933.

Compound	Protein	Buffer	Temp. / °C	Time / h	Conversion (%) *Observed*	Conversion (%) *Previously reported*
2-PCA **1** (100 equiv.)	RNase A (50 µM)	Na phosphate (50 mM, pH 7.5)	37	23	**84**	**90**[3]
2-PCA **2** (100 equiv.)	RNase A (50 µM)	Na phosphate (50 mM, pH 7.5)	37	23	**93** *88(s):5(d)*	**90**[3]
2-PCA **3** (100 equiv.)	RNase A (50 µM)	Na phosphate (50 mM, pH 7.5)	37	23	**94** *86(s):8(d)*	**90**[3]
TA4C **4** (200 equiv.)	RNase A (50 µM)	K phosphate (10 mM, pH 7.5)	37	16	**86** *81(s):5(d)*	**80**[11]
2-EBA **5** (5 equiv.)	RNase A (100 µM)	PBS (50 mM, pH 7.5)	37	16	**15**	**Up to 44**[12]
Ox **6** (50 equiv.)	Myoglobin (3 mM)	Na phosphate (10 mM, pH 7.5)	25	16	**100** *20(s):31(d):31(t):14(q):4(qu)*	**70**[9]
BA **7** (2 equiv.)	RNase A (1.4 mM)	Citric acid (25 mM, pH 6.1)	25	23	**7**	**70 (approx.)**[13]

Table S1. Validation of literature protein modifications. Modification of an example protein with compounds **1-7** under respective reported conditions.

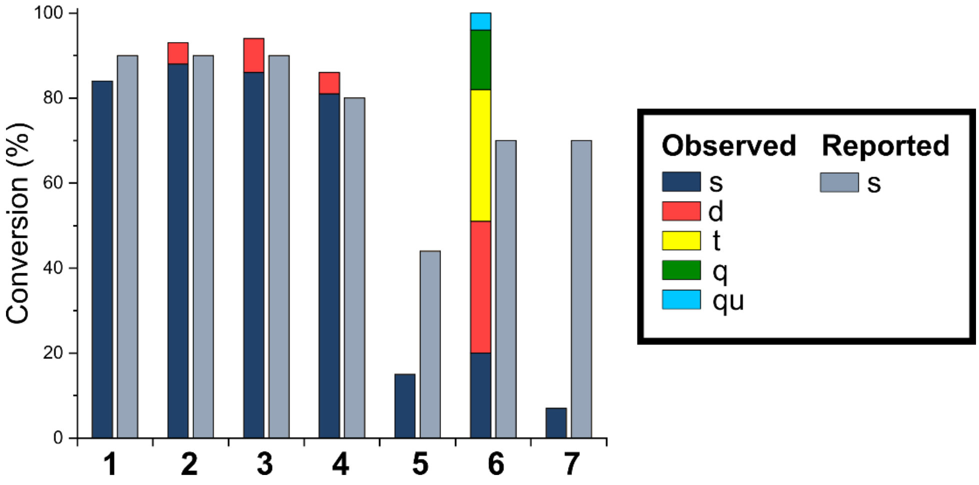

Figure S5. Validation of literature protein modifications. Modification of an example protein with compounds **1-7** under respective reported conditions and comparison to literature reports, as outlined in **Table S1**.

3.2 Screening of reagents under differing conditions

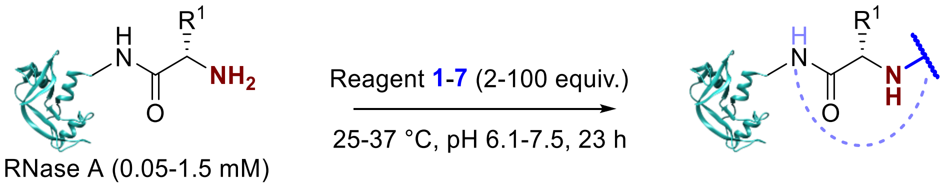

Each of the reagents **1-7** were used to modify RNase A under the conditions outlined in the **General Procedures A**, **C**, **D** and **E** for 23 h.

For the modifications with Procedure A: reagents **1-7** were applied under the conditions outlined in **General Procedure A**. For compound **7**, 5 equiv. of sodium cyanoborohydride in 5 µL of water was also added, and compound **7** was added as a more concentrated solution (45 µL, 11 mM) to keep the total volume constant.

For the modifications with Procedure C: reagents **1-7** were applied under the conditions outlined in **General Procedure C**, on a 50 µL scale. For compound **7**, 5 equiv. of sodium cyanoborohydride in 2.5 µL of water was also added, and compound **7** was added as a more concentrated solution (22.5 µL, 1.1 mM) to keep the total volume constant.

For the modifications with Procedure D: reagents **1-7** were applied under the conditions outlined in **General Procedure D**, on a 20 µL scale, with 1.5 mM final protein concentration and 50 equiv. compound **1-7**. Samples were diluted ×10 with 10 mM pH 7.5 sodium phosphate buffer prior to analysis of conversion by LC-MS analysis without purification. For compound **7**, 5 equiv. of sodium cyanoborohydride in 1 µL of water was also added, and compound **7** was added as a more concentrated solution (9 µL, 167 mM) to keep the total volume constant.

For the modifications with Procedure E: reagents **1-7** were applied under the conditions outlined in **General Procedure E**. For compounds **1-6**, 2.5 µL 25 mM pH 6.1 citrate buffer was added instead of the sodium cyanoborohydride solution.

Compound	**Conversion (%)**			
	General Procedure **A**	General Procedure **C**	General Procedure **D**	General Procedure **E**
2-PCA **1**	**84**	**10**	**29**	**0**
2-PCA **2**	**92**	**84**	**93**<br>*67(s):22(d):5(t)*	**23**
2-PCA **3**	**94**<br>*86(s):8(d)*	**42**	**100**<br>*77(s):23(d)*	**6**
TA4C **4**	**77**	**15**	**48**	**0**

2-EBA **5**	**67** *22(s):29(d):11(t):5(q)*	**6**	**20**	**0**
Ox **6**	**22**	**6**	**87** *54(s):33(d)*	**0**
BA **7**	**10**	**0**	**100** *15(s):33(d):32(t):19(q)*	**7**

Table S2. Screening of reagents under differing conditions. Modification of RNase A with compounds **1-7** under respective reported conditions outlined in the **General Procedures A**, **C**, **D** and **E**.

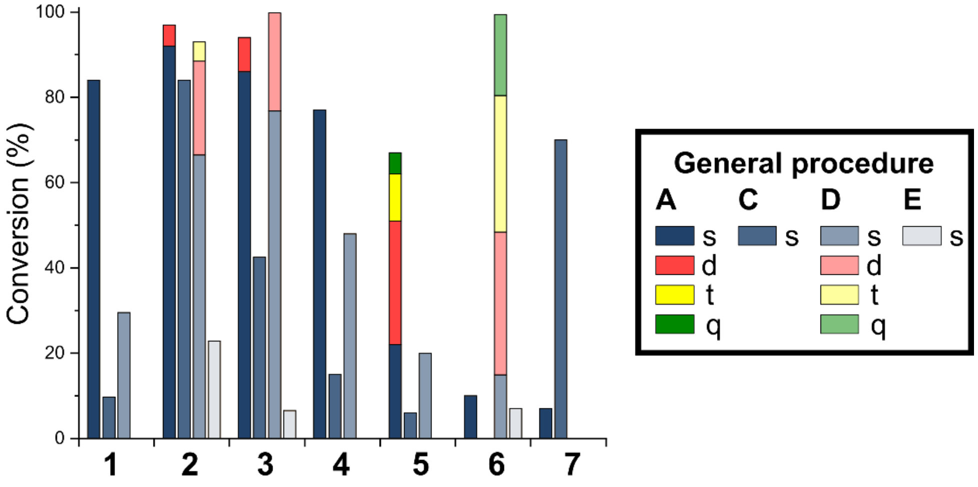

Figure S6. Screening of reagents under differing conditions. Modification of RNase A with compounds **1-7** under respective reported conditions outlined in **Table S2**.

3.3 Optimisation of 2-EBA concentration

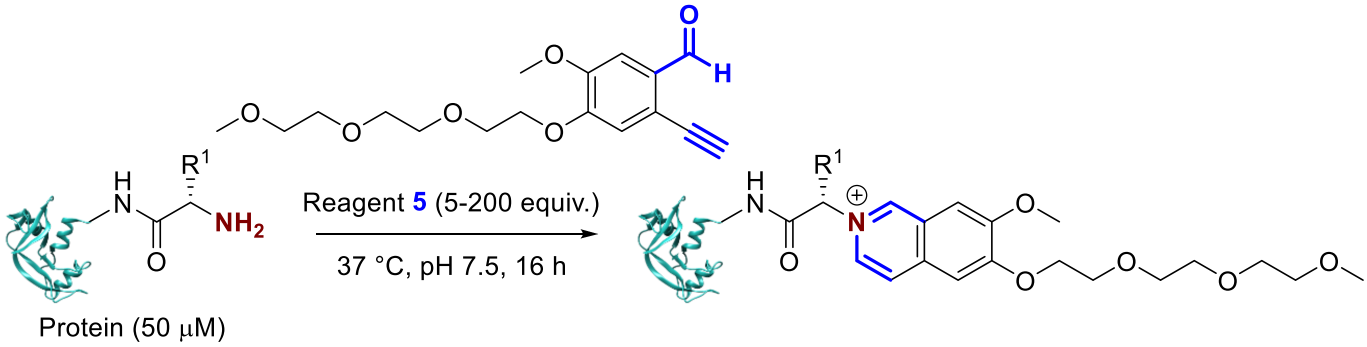

To minimise off-target reactivity with 2-EBA **5**, RNase A and myoglobin were modified according to **General Procedure A**, varying the equiv. of 2-EBA while maintaining all other conditions (5,10, 25,100, 200 equiv.).

Number of 2-EBA equiv.	Conversion (%)	
	RNase A	Myoglobin
5	**7**	**12**
10	**11**	**18**
25	**23** *19(s):4(d)*	**43** *28(s):13(d):2(t)*
100	**47**	**58**

	25(s):13(d):9(t)	36(s):13(d):9(t)
200	No protein detected	

Table S3. Optimisation of 2-EBA concentration. Modification of RNase A and myoglobin with 2-EBA **5** (5, 10, 25, 100 equiv.).

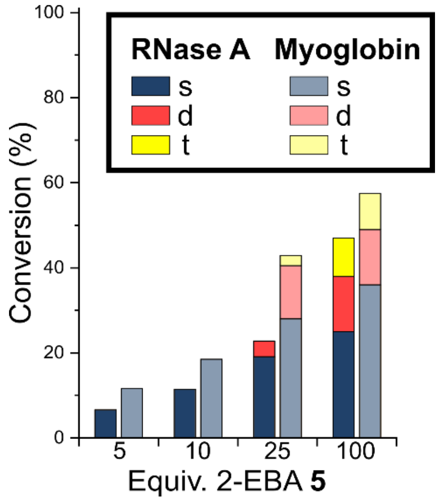

Figure S7. Optimisation of 2-EBA concentration. Modification of RNase A and myoglobin with 2-EBA **5** (5, 10, 25, 100 equiv.).

3.4 Protein panel modification

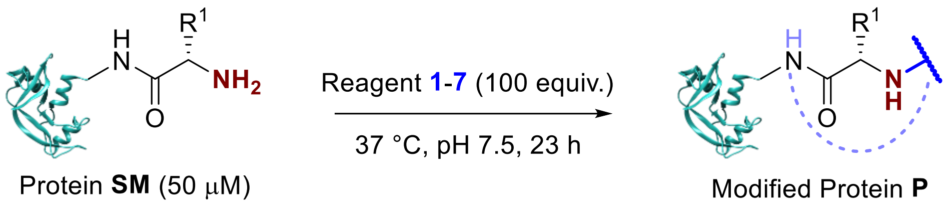

For the modification of RNase A and myoglobin: reagents **1-7** were applied under the conditions outlined in **General Procedure A**. For compound **7**, 5 equiv. of sodium cyanoborohydride in 5 µL of water was also added, and compound **7** was added as a more concentrated solution (45 µL, 11 mM) to keep the total volume constant.

For the modification of Clostripain LC: As described above, on a 19.5 µL scale.

For the modification of CjX183-D WT: As described above, on a 22.9 µL scale.

For the modification of CjX183-D R51K: As described above, on a 30.6 µL scale.

RNase A

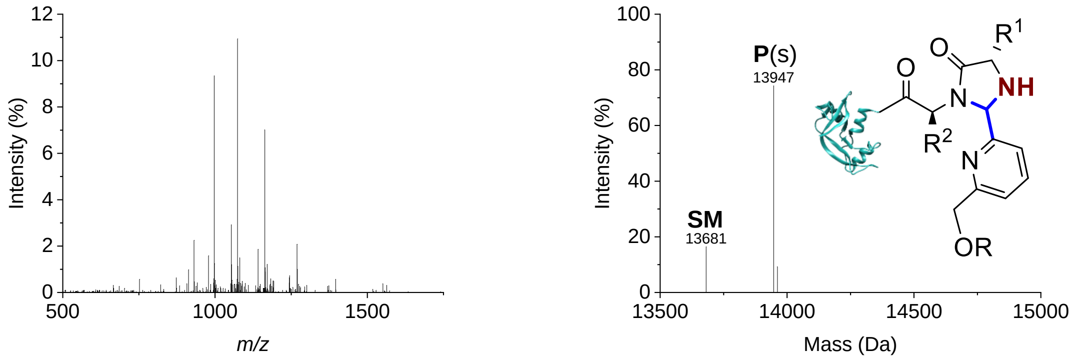

2-PCA **1***

84%

MS (ESI+) [**SM**+H]+ found 13681, calculated 13681;[1] [**P**(s)+H]+ found 13947, calculated 13946; [**P**(s)+H_2O+H]+ found 13962, calculated 13964.

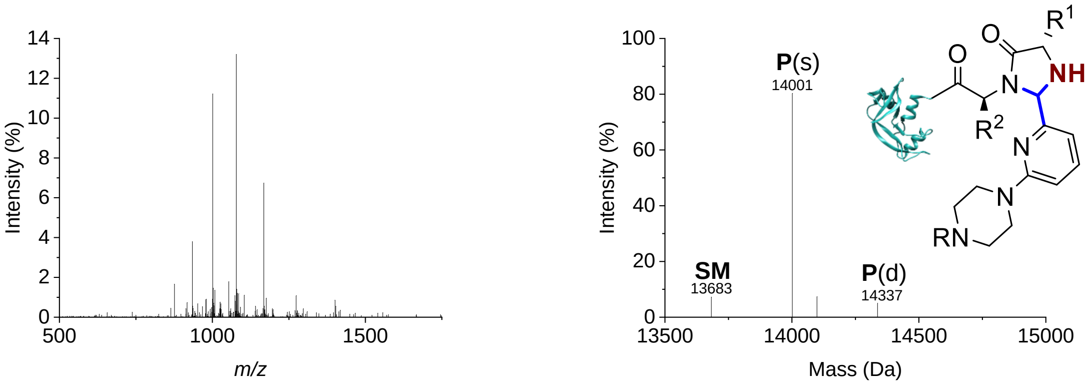

2-PCA **2***

93%

88(s):5(d)

MS (ESI+) [**SM**+H]+ found 13683, calculated 13681;[1] [**P**(s)+H]+ found 14001, calculated 14001; [**P**(s)+H_3PO_4+H]+ found 14099, calculated 14098; [**P**(d)+H_2O+H]+ found 14337, calculated 14338.

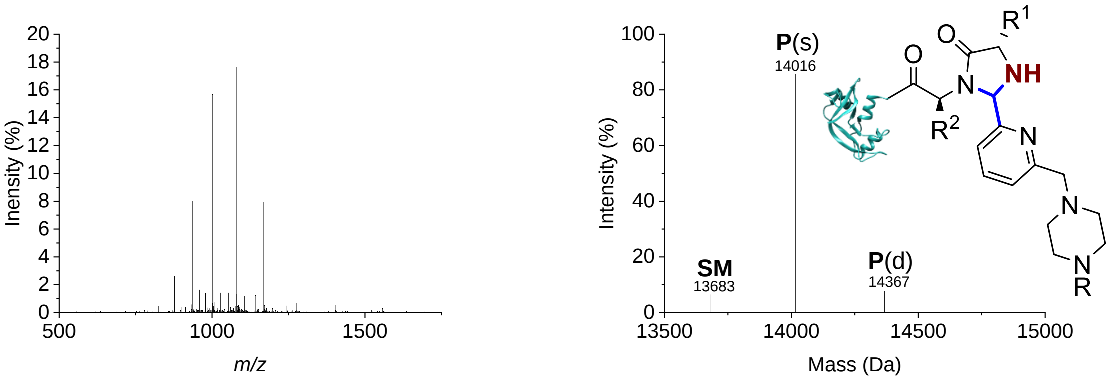

2-PCA **3***

94%

86(s):8(d)

MS (ESI+) [**SM**+H]+ found 13683, calculated 13681;[1] [**P**(s)+H]+ found 14016, calculated 14015; [**P**(d)+H_2O+H]+ found 14367, calculated 14366.

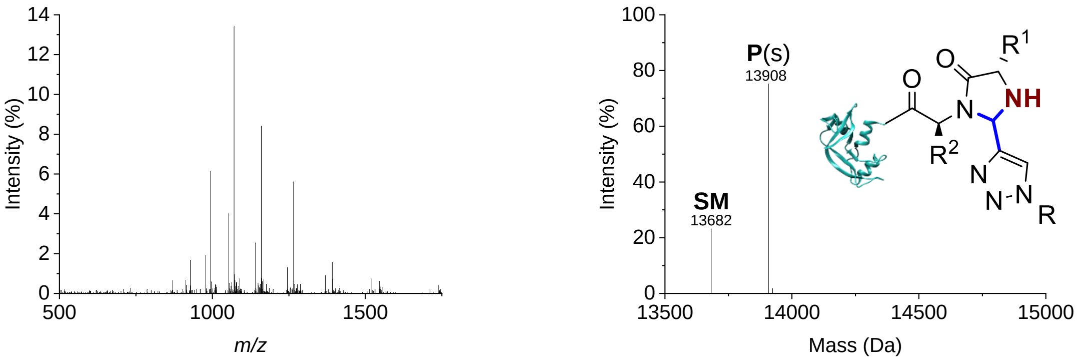

TA4C **4**

77%

MS (ESI+) [**SM**+H]+ found 13682, calculated 13681;[1] [**P**(s)+H]+ found 13908, calculated 13906; [**P**(s)+H_2O+H]+ found 13924, calculated 13924.

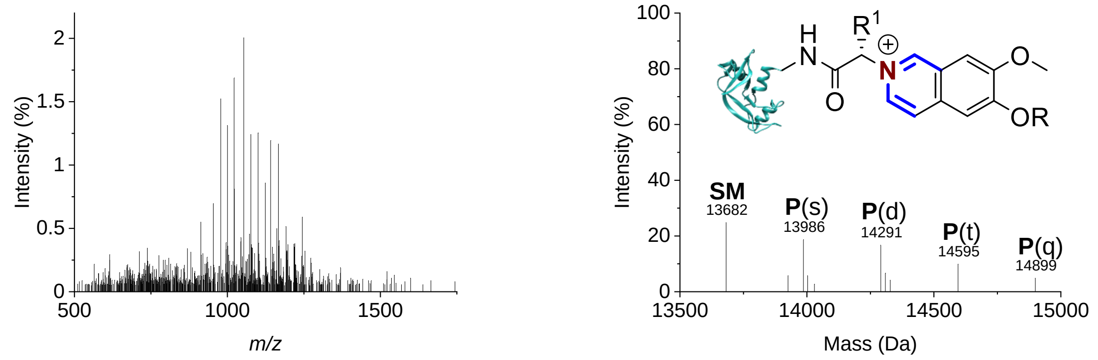

2-EBA **5**

74%

29(s):29(d):

11(t):5(q)

MS (ESI$^+$) [**SM**+H]$^+$ found 13682, calculated 13681;[1] [**P**(s)+H]$^+$ found 13986, calculated 13985; [**P**(s)+H_2O+H]$^+$ found 14003, calculated 14003; [**P**(s)+MeCN+H]$^+$ found 14030, calculated 14026; [**P**(d)+H]$^+$ found 14291, calculated 14290; [**P**(d)+H_2O+H]$^+$ found 14309, calculated 14308; [**P**(d)+K]$^+$ found 14328, calculated 14328; [**P**(t)+H]$^+$ found 14595, calculated 14594; [**P**(q)+H]$^+$ found 14899, calculated 14898.

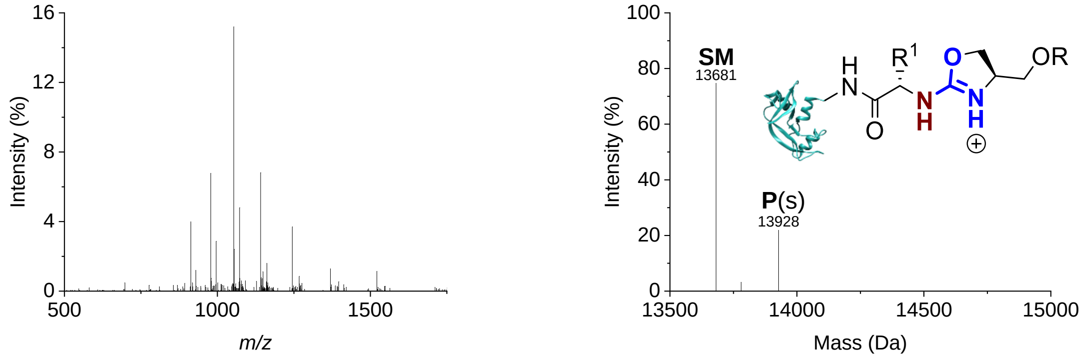

Ox **6**

22%

MS (ESI$^+$) [**SM**+H]$^+$ found 13681, calculated 13681;[1] [**SM**+H_3PO_4+H]$^+$ found 13780, calculated 13779; [**P**(s)+H]$^+$ found 13928, calculated 13926.

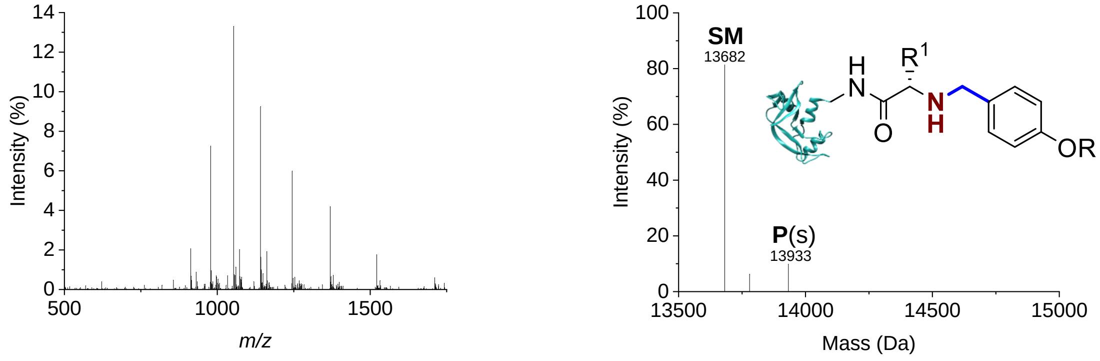

BA **7**

10%

MS (ESI$^+$) [**SM**+H]$^+$ found 13682, calculated 13681;[1] [**SM**+H_3PO_4+H]$^+$ found 13780, calculated 13779; [**P**(s)+H]$^+$ found 13933, calculated 13933.

Myoglobin

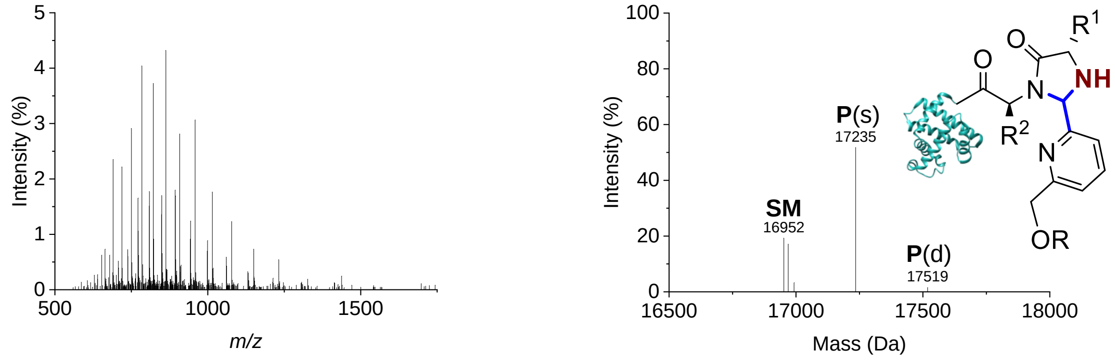

2-PCA **1**

57%

55(s):2(d)

MS (ESI$^+$) [**SM**+H]$^+$ found 16952, calculated 16951;[2] [**SM**+H_2O+H]$^+$ found 16969, calculated 16970; [**SM**+MeCN+H]$^+$ found 16992, calculated 16993; [**P**(s)+H_2O+H]$^+$ found 17235, calculated 17235; [**P**(d)+$2H_2O$+H]$^+$ found 17519, calculated 17518.

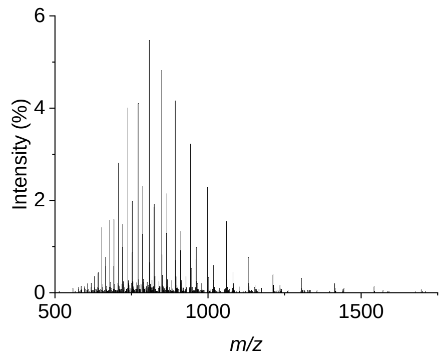

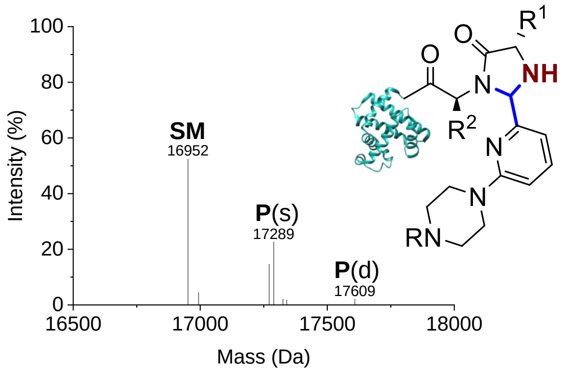

2-PCA **2**

41%

39(s):2(d)

MS (ESI$^+$) [**SM**+H]$^+$ found 16952, calculated 16951;[2] [**SM**+MeCN+H]$^+$ found 16994, calculated 16993; [**P**(s)+H]$^+$ found 17271, calculated 17271; [**P**(s)+H_2O+H]$^+$ found 17289, calculated 17289; [**P**(d)+H_2O+H]$^+$ found 17609, calculated 17608.

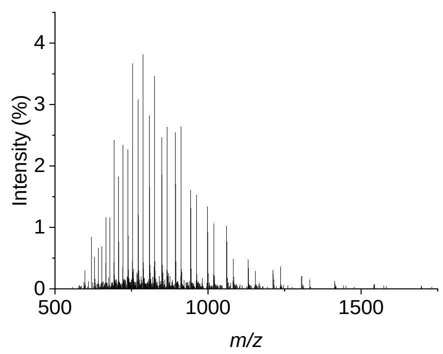

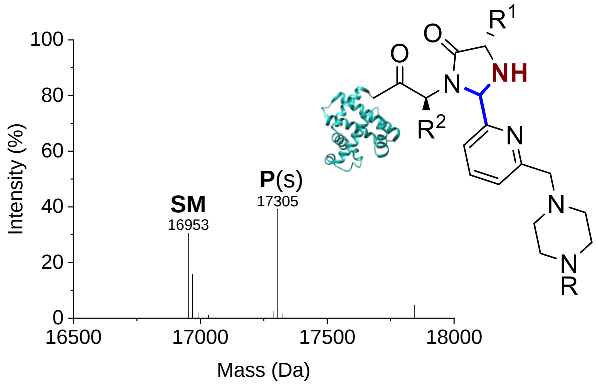

2-PCA **3**

47%

MS (ESI$^+$) [**SM**+H]$^+$ found 16953, calculated 16951;[2] [**SM**+H_2O+H]$^+$ found 16969, calculated 16970; [**SM**+MeCN+H]$^+$ found 16994, calculated 16993; [**SM**+DMSO+H]$^+$ found 17032, calculated 17030; [**P**(s)+H]$^+$ found 17287, calculated 17285; [**P**(s)+H_2O+H]$^+$ found 17305, calculated 17303; [**P**(s)+K]$^+$ found 17323, calculated 17324.

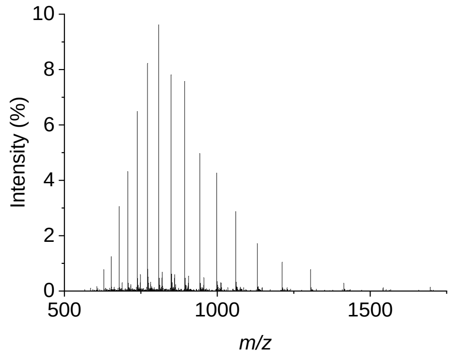

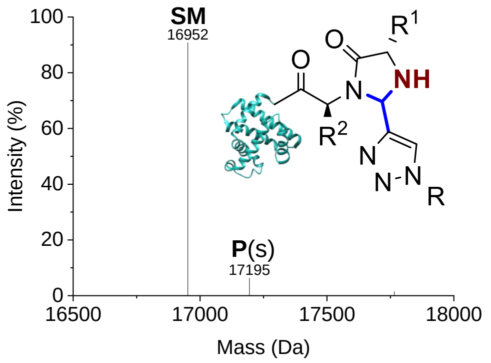

TA4C **4**

6%

MS (ESI$^+$) [**SM**+H]$^+$ found 16952, calculated 16951;[2] [**P**(s)+H_2O+H]$^+$ found 17195, calculated 17195.

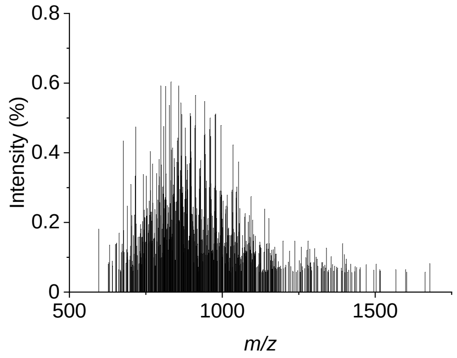

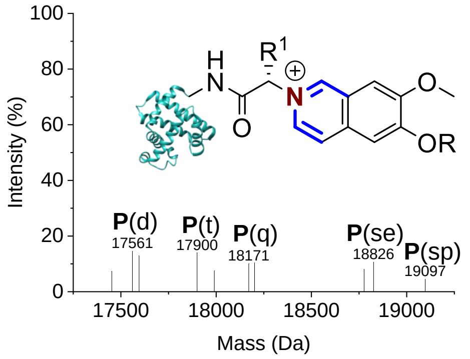

2-EBA **5**

100%

32(d):17(t):

24(q):22(se):

5(sp)

MS (ESI$^+$) [**P**(d)+H]$^+$ found 17561, calculated 17560; [**P**(d)+MeOH+H]$^+$ found 17596, calculated 17592; [**P**(t)+MeOH+H]$^+$ found 17900, calculated 17896; [**P**(q)+H]$^+$ found 18171, calculated 18168; [**P**(q)+MeOH+H]$^+$ found 18202, calculated 18200; [**P**(se)+H]$^+$ found 18776, calculated 18776; [**P**(se)+MeOH+H_2O+H]$^+$ found 18826, calculated 18826; [**P**(sp)+H_2O+H]$^+$ found 19097, calculated 19098.

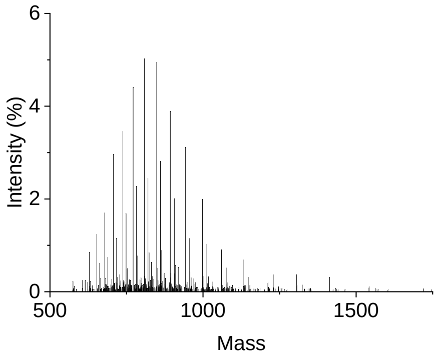

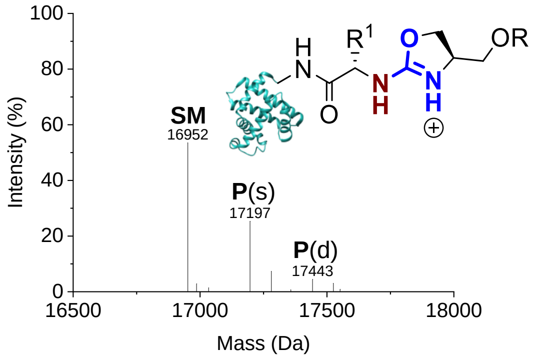

Ox **6**

42%

34(s):8(d)

MS (ESI$^+$) [**SM**+H]$^+$ found 16952, calculated 16951;[2] [**SM**+MeOH+H]$^+$ found 16986, calculated 16984; [**SM**+2MeCN+H]$^+$ found 17034, calculated 17034; [**P**(s)+H]$^+$ found 17197, calculated 17197; [**P**(s)+2MeCN+H]$^+$ found 17281, calculated 17279; [**P**(d)+H]$^+$ found 17443, calculated 17442; [**P**(d)+2MeCN+H]$^+$ found 17552, calculated 17524.

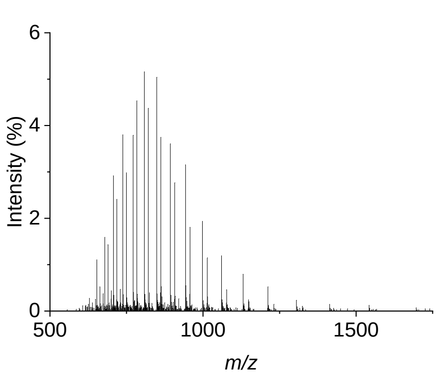

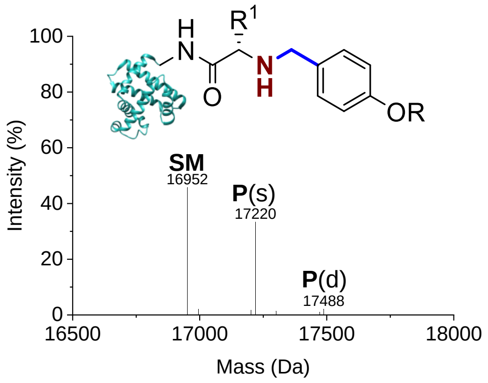

BA **7**

46%

42(s):4(d)

MS (ESI$^+$) [**SM**+H]$^+$ found 16952, calculated 16951;[2] [**SM**+MeCN+H]$^+$ found 16995, calculated 16993; [**P**(s)+H]$^+$ found 17202, calculated 17204; [**P**(s)+H_2O+H]$^+$ found 17220, calculated 17222; [**P**(s)+H_3PO_4+H]$^+$ found 17301, calculated 17302; [**P**(d)+H_2O+H]$^+$ found 17473, calculated 17474; [**P**(d)+MeOH+H]$^+$ found 17488, calculated 17488.

Clostripain LC

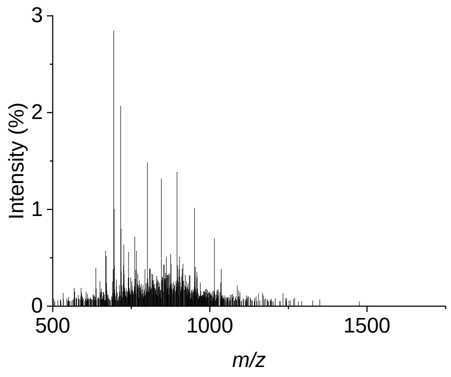

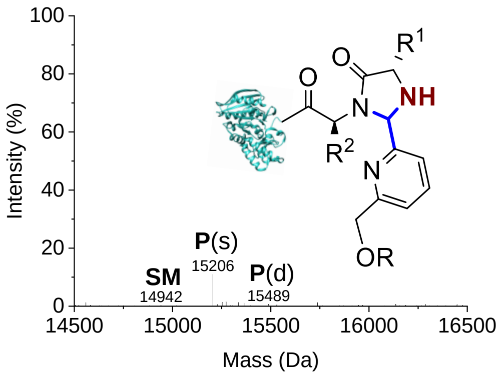

2-PCA **1**

97%

89(s):8(d)

MS (ESI$^+$) [**SM**+H]$^+$ found 14942, calculated 14941;[3] [**SM**+MeCN+H]$^+$ found 14982, calculated 14982; [**P**(s)+H]$^+$ found 15206, calculated 15206; [**P**(s)+Na]$^+$ found 15229, calculated 15228; [**P**(s)+MeCN+H]$^+$ found 15253, calculated 15247; [**P**(d)+H_2O+H]$^+$ found 15489, calculated 15489; [**P**(d)+H_2O+MeCN+H]$^+$ found 15531, calculated 15530.

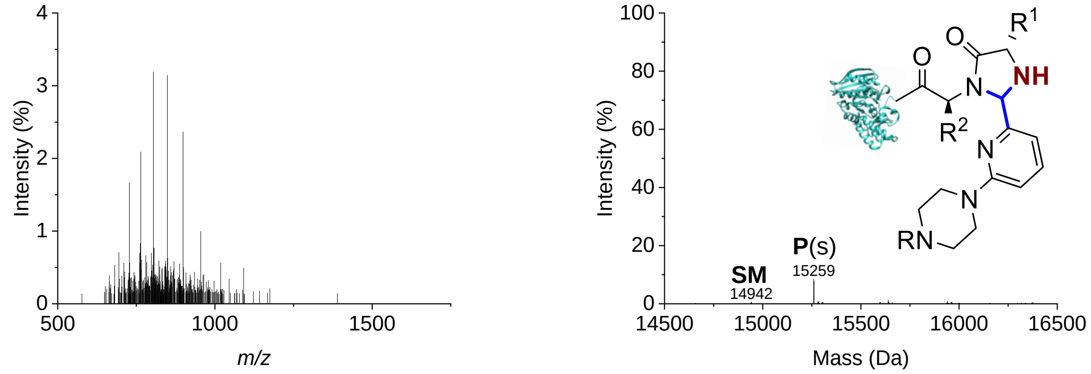

2-PCA **2**

94%

MS (ESI+) [**SM**+H]+ found 14942, calculated 14941;[3] [**P**(s)+H]+ found 15259, calculated 15260; [**P**(s)+Na]+ found 15282, calculated 15282; [**P**(s)+MeCN+H]+ found 15300, calculated 15301.

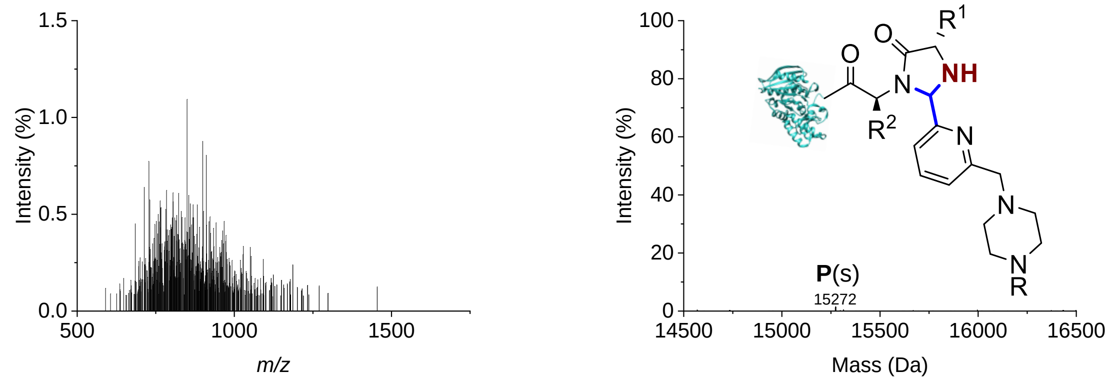

2-PCA **3**

100%

MS (ESI+) [**P**(s)+H]+ found 15272, calculated 15274.

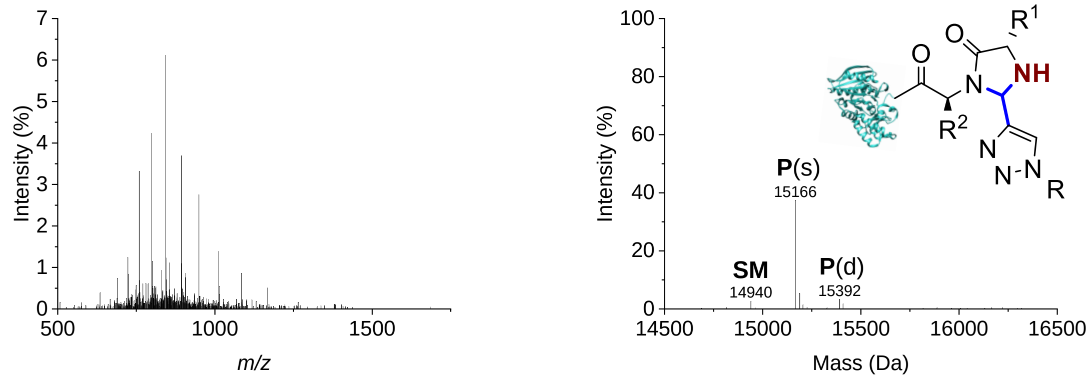

TA4C **4**

90%

84(s):6(d)

MS (ESI+) [**SM**+H]+ found 14940, calculated 14941;[3] [**SM**+Na]+ found 14962, calculated 14963; [**SM**+MeCN+H]+ found 14983, calculated 14982; [**P**(s)+H]+ found 15166, calculated 15166; [**P**(s)+Na]+ found 15188, calculated 15189; [**P**(s)+MeCN+H]+ found 15205, calculated 15207; [**P**(d)+H]+ found 15392, calculated 15391; [**P**(d)+H_2O+H]+ found 15409, calculated 15409; [**P**(d)+2H_2O+H]+ found 15428, calculated 15427.

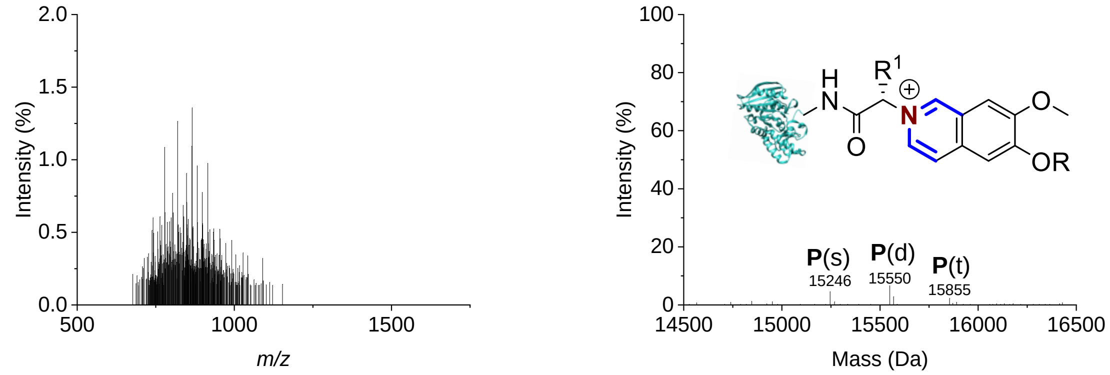

2-EBA **5***

100%

24(s):37(d):

38(t)

MS (ESI$^+$) [**P**(s)+H]$^+$ found 15246, calculated 15245; [**P**(s)+Na]$^+$ found 15268, calculated 15267; [**P**(d)+H]$^+$ found 15550, calculated 15549; [**P**(d)+H_2O+H]$^+$ found 15569, calculated 15567; [**P**(d)+K]$^+$ found 15587, calculated 15587; [**P**(t)+H]+ found 15855, calculated 15853; [**P**(t)+H_2O+H]$^+$ found 15871, calculated 15871; [**P**(t)+2H_2O+H]$^+$ found 15890, calculated 15889.

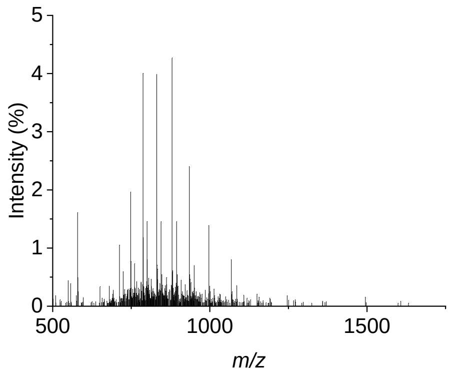

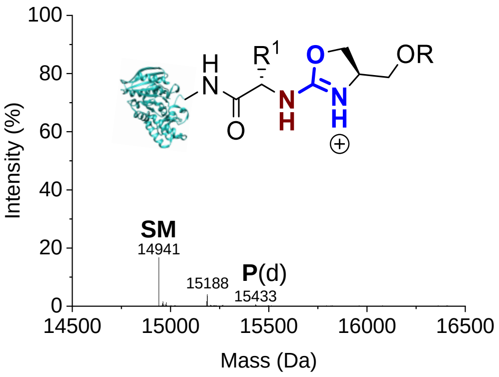

Ox **6**

37%

34(s):3(d)

MS (ESI$^+$) [**SM**+H]$^+$ found 14941, calculated 14941;[3] [**SM**+H_2O+H]$^+$ found 14959, calculated 14959; [**SM**+Na]$^+$ found 14962, calculated 14963; [**P**(s)+H]$^+$ found 15188, calculated 15187; [**P**(s)+H_2O+H]$^+$ found 15204, calculated 15205; [**P**(s)+Na]$^+$ found 15206, calculated 15209; [**P**(d)+H]$^+$ found 15433, calculated 15433.

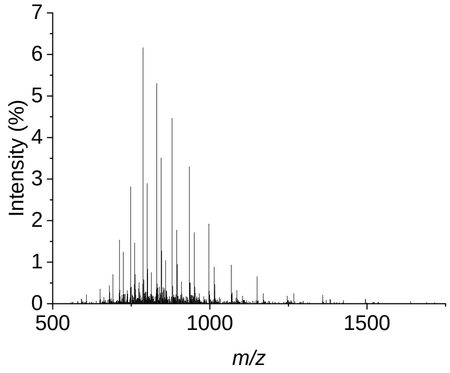

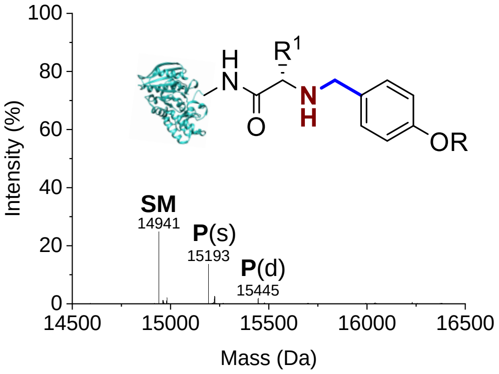

BA **7**

37%

29(s):7(d)

1(t)

MS (ESI$^+$) [**SM**+H]$^+$ found 14941, calculated 14941;[3] [**SM**+H_2O+H]$^+$ found 14960, calculated 14959; [**SM**+Na]$^+$ found 14962, calculated 14963; [**SM**+K]$^+$ found 14979, calculated 14979; [**SM**+MeCN+H]$^+$ found 14983, calculated 14982; [**P**(s)+H]$^+$ found 15193, calculated 15192; [**P**(s)+H_2O+H]$^+$ found 15211, calculated 15211; [**P**(s)+Na]$^+$ found 15215, calculated 15215; [**P**(s)+K]$^+$ found 15232, calculated 15230; [**P**(d)+H]$^+$ found 15445, calculated 15443; [**P**(t)+H]$^+$ found 15696, calculated 15694.

CjX183-D WT

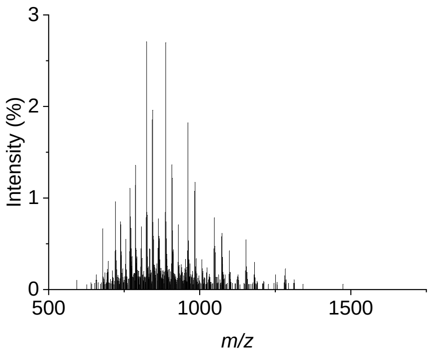

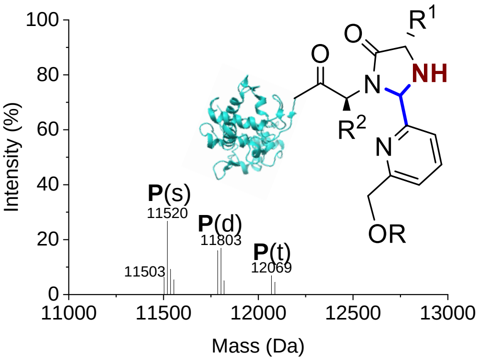

2-PCA **1***

100%

49(s):39(d):

12(t)

MS (ESI$^+$) [**P**(s)+H]$^+$ found 11503, calculated 11491; [**P**(s)+H_2O+H]$^+$ found 11520, calculated 11521; [**P**(s)+2H_2O+H]$^+$ found 11537, calculated 11539; [**P**(s)+3H_2O+H]$^+$ found 11555, calculated 11557; [**P**(d)+H_2O+H]$^+$ found 11786, calculated 11786; [**P**(d)+2H_2O+H]$^+$ found 11803, calculated 11804; [**P**(d)+3H_2O+H]$^+$ found 11820, calculated 11822; [**P**(t)+2H_2O+H]$^+$ found 12069, calculated 12069; [**P**(t)+3H_2O+H]$^+$ found 12088, calculated 12087.

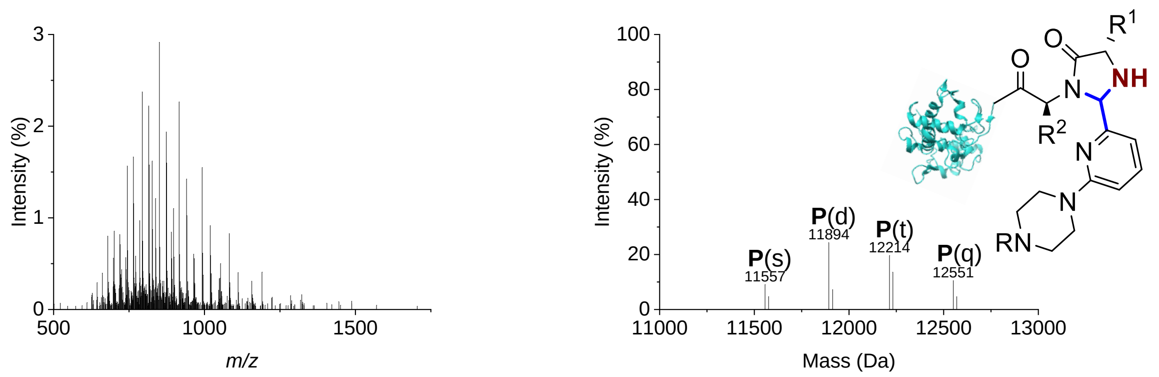

2-PCA **2**

100%

15(s):34(d):

35(t):16(q)

MS (ESI$^+$) [**P**(s)+H]$^+$ found 11557, calculated 11545; [**P**(s)+H_2O+H]$^+$ found 11576, calculated 11575; [**P**(d)+H_2O+H]$^+$ found 11894, calculated 11894; [**P**(d)+K]$^+$ found 11914, calculated 11914; [**P**(t)+H_2O+H]$^+$ found 12214, calculated 12213; [**P**(t)+2H_2O+H]$^+$ found 12232, calculated 12231; [**P**(q)+2H_2O+H]$^+$ found 12551, calculated 12550; [**P**(q)+3H_2O+H]$^+$ found 12569, calculated 12568.

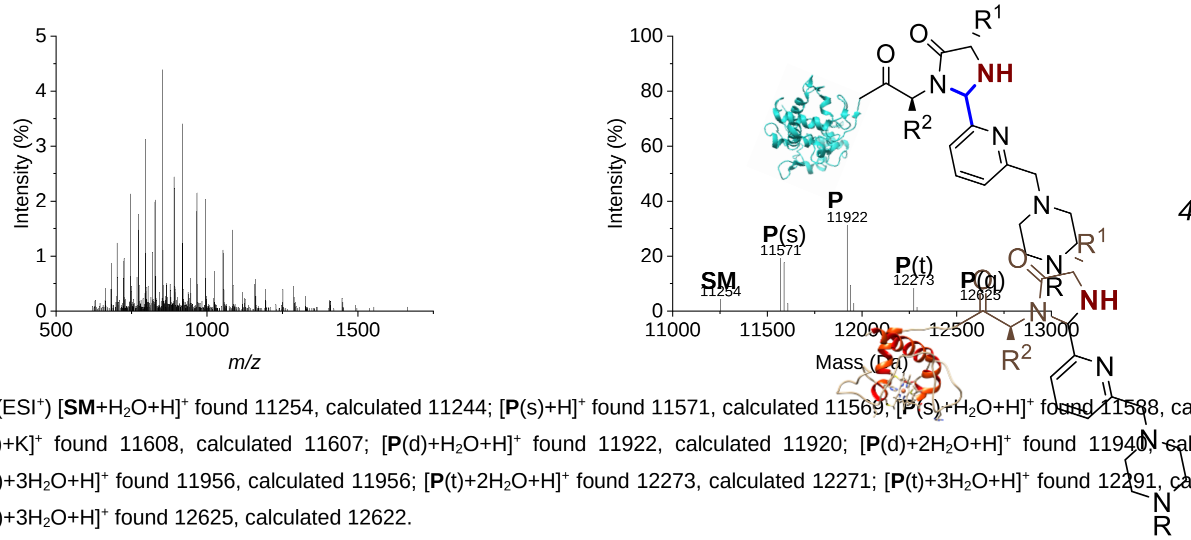

2-PCA **3**

96%

40(s):43(d):

10(t):3(q)

MS (ESI$^+$) [**SM**+H_2O+H]$^+$ found 11254, calculated 11244; [**P**(s)+H]$^+$ found 11571, calculated 11569; [**P**(s)+H_2O+H]$^+$ found 11588, calculated 11587; [**P**(s)+K]$^+$ found 11608, calculated 11607; [**P**(d)+H_2O+H]$^+$ found 11922, calculated 11920; [**P**(d)+2H_2O+H]$^+$ found 11940, calculated 11938; [**P**(d)+3H_2O+H]$^+$ found 11956, calculated 11956; [**P**(t)+2H_2O+H]$^+$ found 12273, calculated 12271; [**P**(t)+3H_2O+H]$^+$ found 12291, calculated 12289; [**P**(q)+3H_2O+H]$^+$ found 12625, calculated 12622.

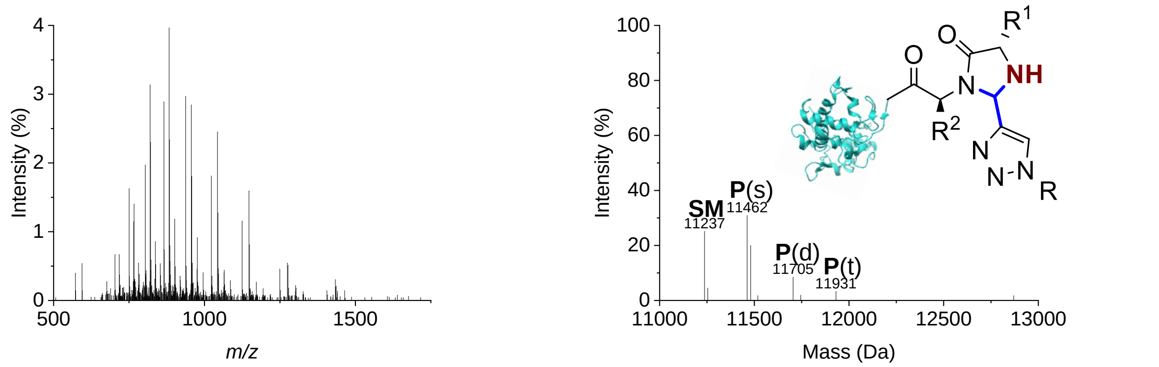

TA4C **4**

69%

55(s):11(d):

3(t)

MS (ESI$^+$) [**SM**+H]$^+$ found 11237, calculated 11226; [**SM**+H_2O+H]$^+$ found 11254, calculated 11255; [**P**(s)+H]$^+$ found 11462, calculated 11462; [**P**(s)+H_2O+H]$^+$ found 11481, calculated 11480; [**P**(s)+H_2O+K]$^+$ found 11519, calculated 11518; [**P**(d)+H_2O+H]$^+$ found 11705, calculated 11705; [**P**(d)+H_2O+K]$^+$ found 11745, calculated 11743; [**P**(t)+H_2O+H]$^+$ found 11931, calculated 11930.

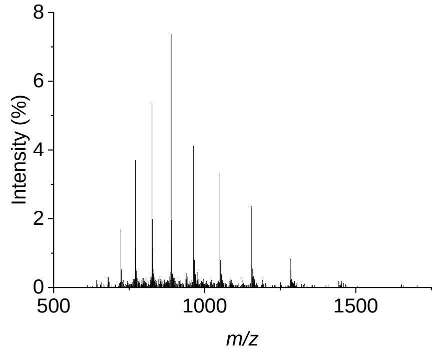

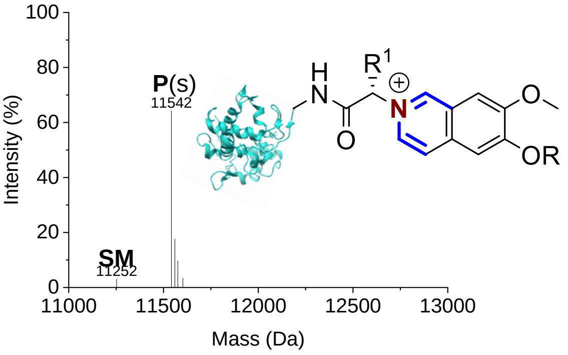

2-EBA **5**

97%

MS (ESI$^+$) [**SM**+H_2O+H]$^+$ found 11252, calculated 11244; [**P**(s)+H]$^+$ found 11542, calculated 11539; [**P**(s)+H_2O+H]$^+$ found 11560, calculated 11557; [**P**(s)+K]$^+$ found 11575, calculated 11577; [**P**(s)+MeCN+Na]$^+$ found 11603, calculated 11602.

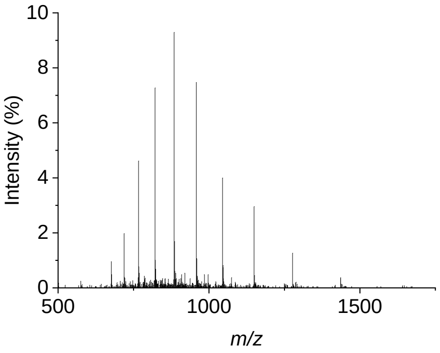

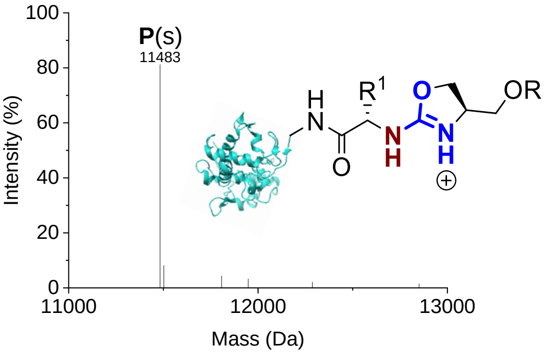

Ox **6**

100%

MS (ESI$^+$) [**P**(s)+H]$^+$ found 11483, calculated 11471; [**P**(s)+H_2O+H]$^+$ found 11503, calculated 11501.

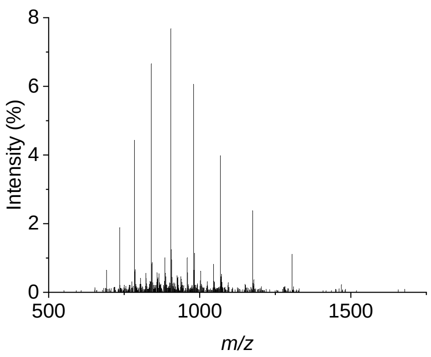

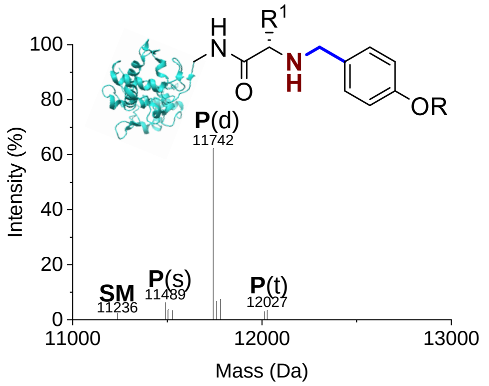

BA **7**

98%

13(s):78(d): 6(t)

MS (ESI$^+$) [**SM**+H]$^+$ found 11236, calculated 11226; [**P**(s)+H]$^+$ found 11489, calculated 11488; [**P**(s)+H_2O+H]$^+$ found 11504, calculated 11506; [**P**(s)+K]$^+$ found 11527, calculated 11526; [**P**(d)+H]$^+$ found 11742, calculated 11741; [**P**(d)+H_2O+H]$^+$ found 11761, calculated 11759; [**P**(d)+K]$^+$ found 11781, calculated 11778; [**P**(t)+H_2O+H]$^+$ found 12012, calculated 12011; [**P**(t)+K]$^+$ found 12027, calculated 12031.

CjX183-D R51K

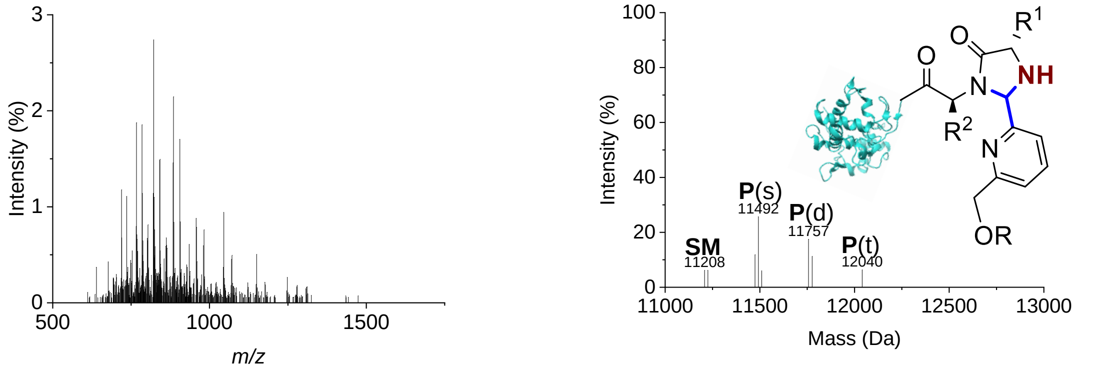

2-PCA **1***

86%

48(s):32(d):7(t)

MS (ESI$^+$) [**SM**+H]$^+$ found 11208, calculated 11198; [**SM**+H_2O+H]$^+$ found 11225, calculated 11226; [**P**(s)+H]$^+$ found 11474, calculated 11473; [**P**(s)+H_2O+H]$^+$ found 11492, calculated 11491; [**P**(s)+2H_2O+H]$^+$ found 11510, calculated 11509; [**P**(d)+H_2O+H]$^+$ found 11757, calculated 11756; [**P**(d)+2H_2O+H]$^+$ found 11776, calculated 11774; [**P**(t)+2H_2O+H]$^+$ found 12040, calculated 12039.

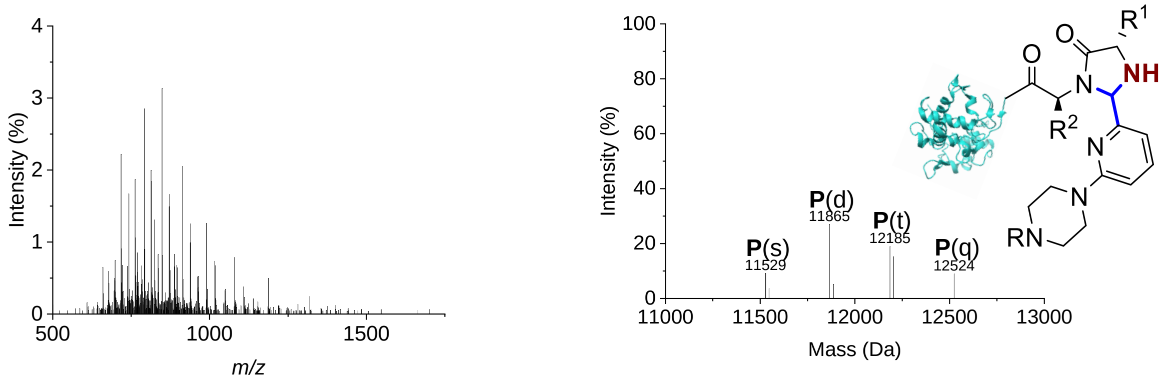

2-PCA **2**

100%

15(s):36(d):39(t):10(q)

MS (ESI$^+$) [**P**(s)+H]$^+$ found 11529, calculated 11517; [**P**(s)+H_2O+H]$^+$ found 11547, calculated 11547; [**P**(d)+H_2O+H]$^+$ found 11865, calculated 11866; [**P**(d)+2H_2O+H]$^+$ found 11887, calculated 11884; [**P**(t)+H_2O+H]$^+$ found 12185, calculated 12185; [**P**(t)+2H_2O+H]$^+$ found 12203, calculated 12203; [**P**(q)+2H_2O+H]$^+$ found 12524, calculated 12522.

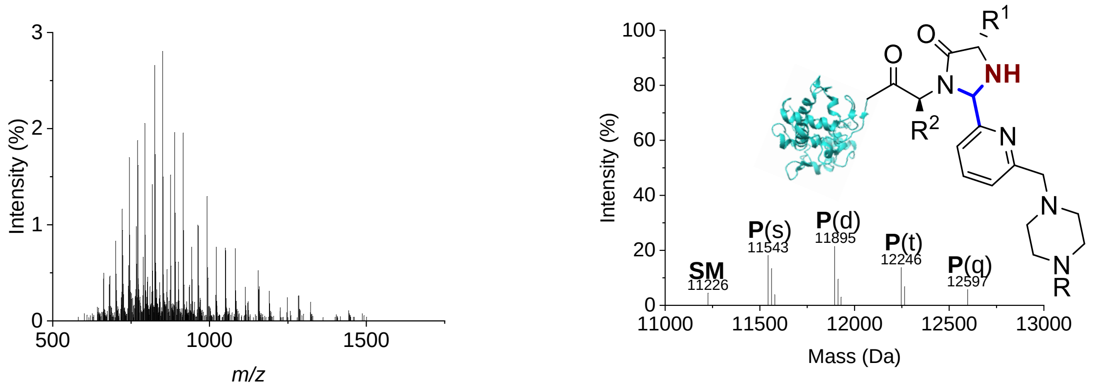

2-PCA **3**

95%

35(s):34(d):21(t):q(6)

MS (ESI$^+$) [**SM**+H_2O+H]$^+$ found 11226, calculated 11216; [**P**(s)+H]$^+$ found 11543, calculated 11541; [**P**(s)+H_2O+H]$^+$ found 11562, calculated 11559; [**P**(s)+2H_2O+H]$^+$ found 11579, calculated 11577; [**P**(d)+H_2O+H]$^+$ found 11895, calculated 11892; [**P**(d)+2H_2O+H]$^+$ found 11913, calculated 11910; [**P**(d)+3H_2O+H]$^+$ found 11929, calculated 11928; [**P**(t)+2H_2O+H]$^+$ found 12246, calculated 12244; [**P**(t)+3H_2O+H]$^+$ found 12264, calculated 12262; [**P**(q)+3H_2O+H]$^+$ found 12597, calculated 12595.

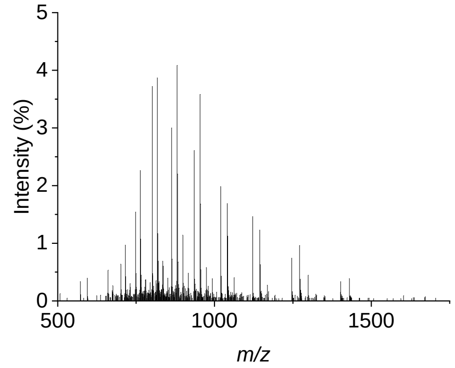

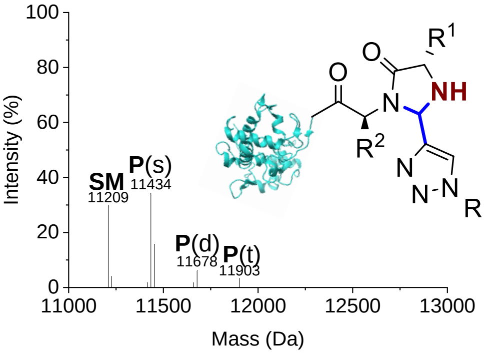

TA4C **4**

65%

54(s):8(d):3(t)

MS (ESI$^+$) [**SM**+H]$^+$ found 11209, calculated 11198; [**SM**+H_2O+H]$^+$ found 11226, calculated 11227; [**P**(s)–H_2O+H]$^+$ found 11417, calculated 11416; [**P**(s)+H]$^+$ found 11434, calculated 11434; [**P**(s)+H_2O+H]$^+$ found 11453, calculated 11452; [**P**(d)+H]$^+$ found 11658, calculated 11659; [**P**(d)+H_2O+H]$^+$ found 11678, calculated 11677; [**P**(t)+H_2O+H]$^+$ found 11903, calculated 11902.

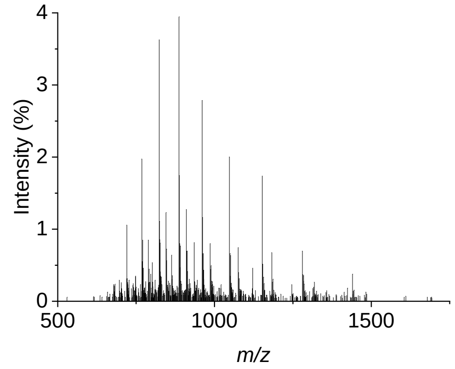

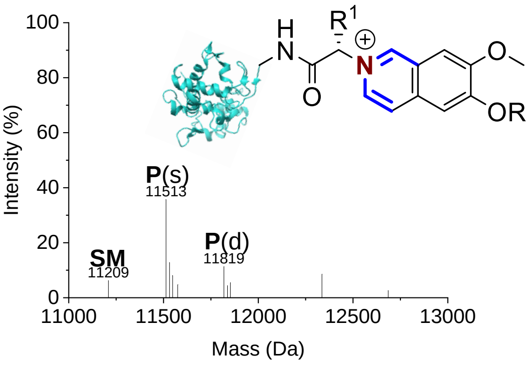

2-EBA **5**

92%

69(s):24(d)

MS (ESI$^+$) [**SM**+H]$^+$ found 11209, calculated 11198; [**P**(s)+H]$^+$ found 11513, calculated 11513; [**P**(s)+H_2O+H]$^+$ found 11532, calculated 11531; [**P**(s)+2H_2O+H]$^+$ found 11548, calculated 11549; [**P**(s)+MeCN+Na]$^+$ found 11574, calculated 11576; [**P**(d)+H]$^+$ found 11819, calculated 11818; [**P**(d)+H_2O+H]$^+$ found 11838, calculated 11836; [**P**(d)+2H_2O+H]$^+$ found 11853, calculated 11854.

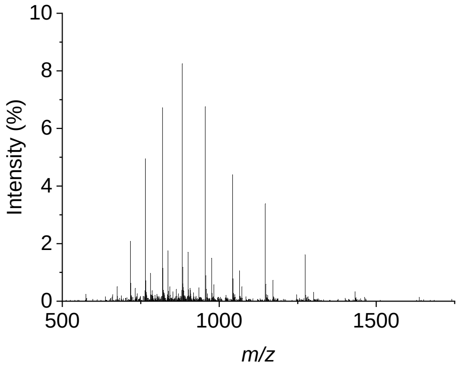

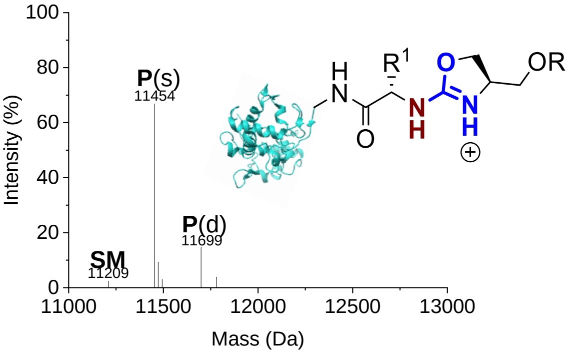

Ox **6**

98%

79(s):18(d)

MS (ESI$^+$) [**SM**+H]$^+$ found 11209, calculated 11198; [**P**(s)+H]$^+$ found 11454, calculated 11455; [**P**(s)+H_2O+H]$^+$ found 11473, calculated 11473; [**P**(s)+K]$^+$ found 11494, calculated 11492; [**P**(d)+H]$^+$ found 11699, calculated 11700; [**P**(d)+2MeCN+H]$^+$ found 11780, calculated 11782.

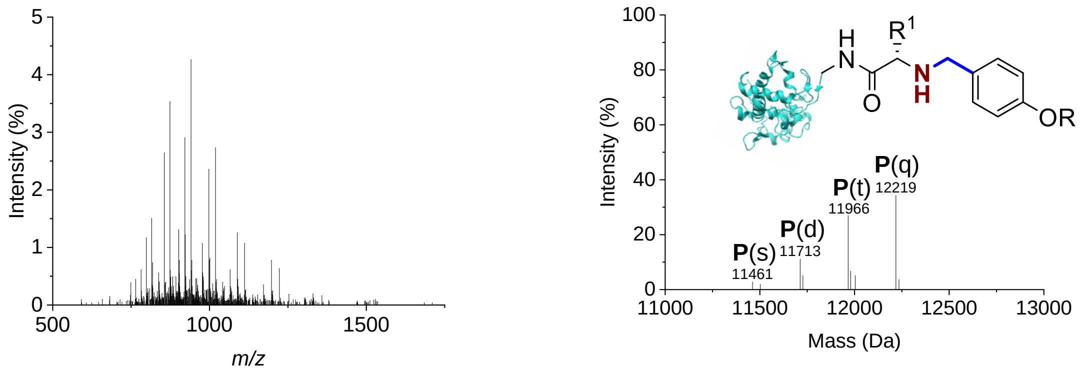

BA **7**

100%

5(s):16(d):

40(t):39(q)

MS (ESI$^+$) [**P**(s)+H]$^+$ found 11461, calculated 11450; [**P**(s)+MeCN+H]$^+$ found 11502, calculated 11502; [**P**(d)+H]$^+$ found 11713, calculated 11713; [**P**(d)+H_2O+H]$^+$ found 11728, calculated 11731; [**P**(t)+H]$^+$ found 11966, calculated 11965; [**P**(t)+H_2O+H]$^+$ found 11980, calculated 11983; [**P**(t)+K]$^+$ found 12004, calculated 12003; [**P**(q)+H]$^+$ found 12219, calculated 12217; [**P**(q)+H_2O+H]$^+$ found 12235, calculated 12235.

**Following LC-MS of each protein sample, water was injected into the LC column to remove residual protein; for samples marked (*), conversions were determined from MS traces of residual protein due to co-elution with excess small molecule.*

4. Conjugate stability

4.1 pH and temperature stability

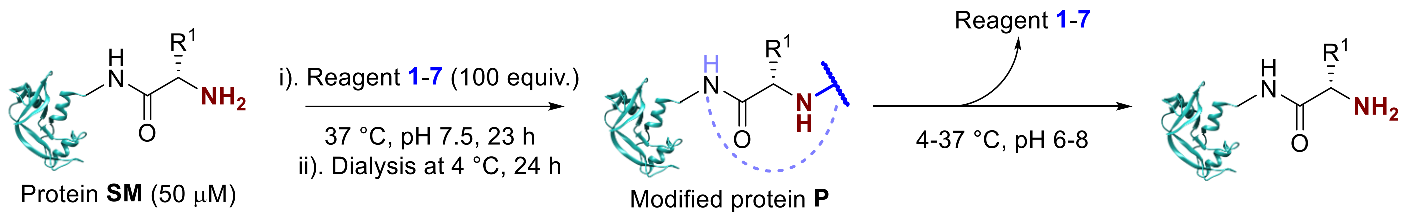

RNase A, myoglobin, and CjX183-D WT were modified with compounds **1-7** according to **General Procedure A**, on a 150 µL scale. For compound **7**, 5 equiv. of sodium cyanoborohydride in 7.5 µL of water was also added, and compound **7** was added as a more concentrated solution (67.5 µL, 11 mM) to keep the total volume constant. For modifications of RNase A and myoglobin, 25 equiv. 2-EBA **5** was used. For the modification of myoglobin, 67 equiv. 2-PCA **2** were used. Protein conjugates **P** were purified by dialysis to remove excess reagent (4 °C, 3.5 kDa MWCO; 1 × 100 mM PBS, pH 7.5, 4 hrs; 2 × water, 4 hr; 1 × water, 16 hr).

Following purification, aliquots of protein (20 µL) were diluted with water (80 µL), placed inside a 3.5 kDa MWCO dialysis membrane, and incubated in sodium phosphate buffer (40 mL, 50 mM) at either pH 6, 7, or 8, and at either 4°C, 22 °C, or 37 °C. Samples were taken from the dialysis membranes at t = 0, 48, and 168 h, and analysed via LC-MS.

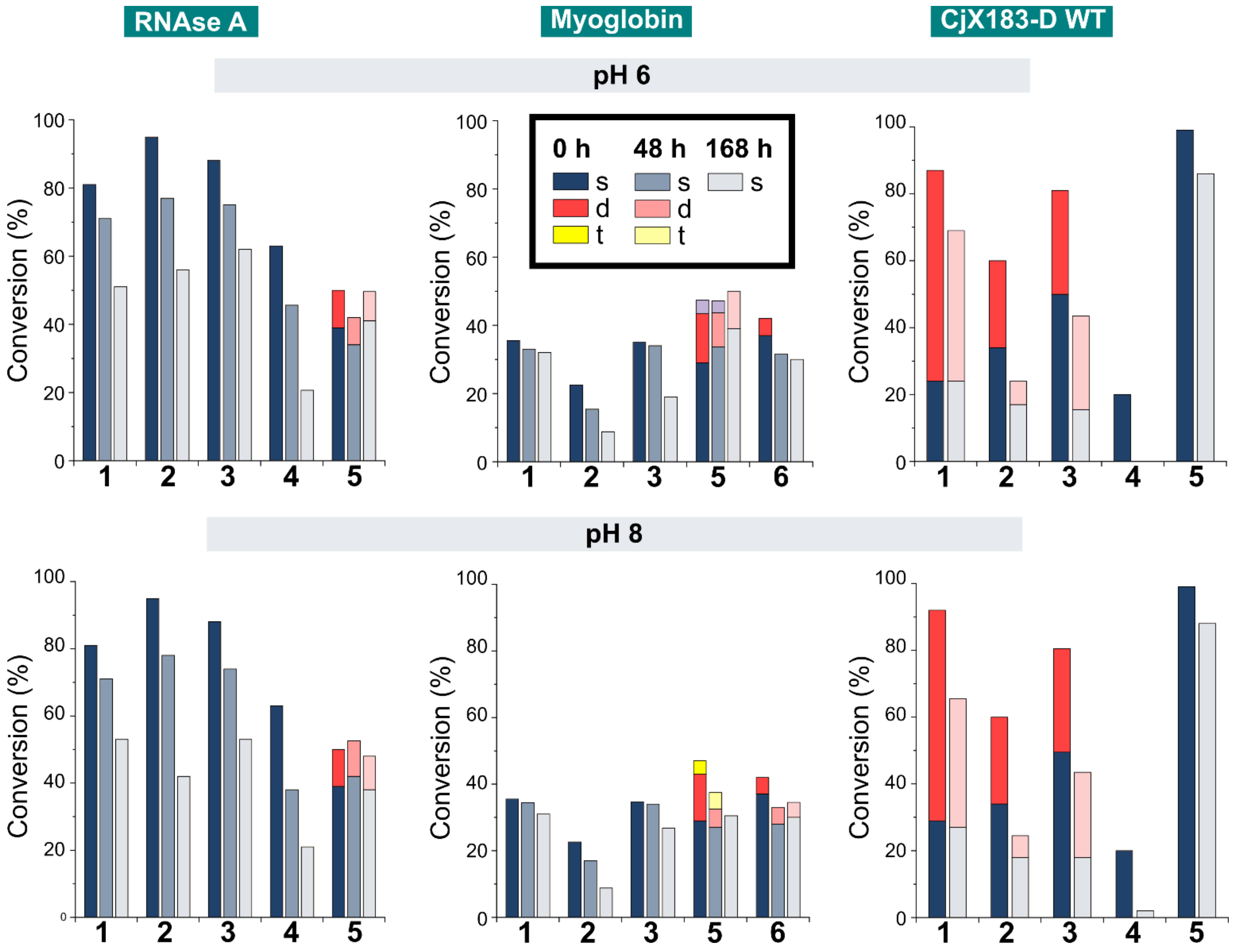

Figure S8. Stability of protein conjugates to pH over time. Modification of RNase A, myoglobin, and CjX183-D WT with compounds **1-7** under conditions outlined in **General Procedure A**, and conversions observed over time upon exposure to a range of conditions (pH 6-8, 22 °C).

Temp. /°C	pH	Protein	Compound	Conversion (%) 0 h	48 h	168 h
4	7	RNase A	2-PCA **1**	**81**	**79**	**79**
			2-PCA **2**	**84**	**90**	**82**
			2-PCA **3**	**88**	**88**	**82** *80(s):3(d)*
			TA4C **4**	**63**	**59**	**51**
			2-EBA **5**	**49** *39(s):11(d)*	**45** *34(s):11(d)*	**43** *40(s):3(d)*
			Ox **6**	**0**	-	-
			BA **7**	**4**	**0**	-
4	7	Myoglobin	2-PCA **1**	**35**	**35**	**29**
			2-PCA **2**	**23**	**15**	**13**
			2-PCA **3**	**35**	**31**	**29**
			TA4C **4**	**0**	-	-
			2-EBA **5**	**48** *29(s):14(d):4(t)*	**38** *33(s):6(d)*	**25**

			Ox **6**	**42**<br>*37(s):5(d)*	**30**<br>*26(s):4(d)*	**40**<br>*36(s):4(d)*
			BA **7**	**0**	-	-
			2-PCA **1**	**87**<br>*24(s):63(d)*	**74**<br>*31(s):43(d)*	**80**<br>*13(s):68(d)*
			2-PCA **2**	**60**<br>*34(s):26(d)*	**34**<br>*18(s):16(d)*	**31**<br>*14(s):17(d)*
4	7	CjX183-D WT	2-PCA **3**	**81**<br>*50(s):31(d)*	**67**<br>*37(s):30(d)*	**58**<br>*31(s):27(d)*
			TA4C **4**	**20**	**8**	**4**
			2-EBA **5**	**100**	**81**	**89**
			Ox **6**	**2**	-	-
			BA **7**	**0**	-	-
			2-PCA **1**	**81**	**61**	**56**
			2-PCA **2**	**84**	**81**	**58**
			2-PCA **3**	**88**	**77**	**61**
22	7	RNase A	TA4C **4**	**63**	**46**	**23**
			2-EBA **5**	**49**<br>*39(s):11(d)*	**49**<br>*40(s):10(d)*	**35**<br>*29(s):6(d)*
			Ox **6**	**0**	-	-
			BA **7**	**4**	**0**	-
			2-PCA **1**	**35**	**31**	**36**
			2-PCA **2**	**23**	**19**	**14**
			2-PCA **3**	**35**	**34**	**24**
			TA4C **4**	**0**	-	-
22	7	Myoglobin	2-EBA **5**	**48**<br>*29(s):14(d):4(t)*	**35**<br>*30(s):5(d)*	**30**<br>*16(s):14(d)*
			Ox **6**	**42**<br>*37(s):5(d)*	**39**<br>*30(s):9(d)*	**36**<br>*30(s):6(d)*
			BA **7**	**0**	-	-
			2-PCA **1**	**87**<br>*24(s):63(d)*	-	**70**<br>*19(s):51(d)*
			2-PCA **2**	**60**<br>*34(s):26(d)*	-	**23**<br>*16(s):7(d)*
22	7	CjX183-D WT	2-PCA **3**	**81**<br>*50(s):31(d)*	-	**62**<br>*13(s):25(d)*
			TA4C **4**	**20**	-	**5**
			2-EBA **5**	**100**	-	*87(s):3(d):2(t)*
			Ox **6**	**2**	-	-
			BA **7**	**0**	-	-
			2-PCA **1**	**81**	**26**	**9**
			2-PCA **2**	**84**	**16**	**0**
37	7	RNase A	2-PCA **3**	**88**	**40**	**13**<br>*0(s):13(d)*
			TA4C **4**	**63**	**9**	**0**
			2-EBA **5**	**49**<br>*39(s):11(d)*	**44**<br>*36(s):8(d)*	**22**

			Ox **6**	**0**	-	-
			BA **7**	**4**	**0**	-
37	7	Myoglobin	2-PCA **1**	**35**	**30**	**30**
			2-PCA **2**	**23**	**7**	**10**
			2-PCA **3**	**35**	**28**	**23**
			TA4C **4**	**0**	-	-
			2-EBA **5**	**48**<br>*29(s):14(d):4(t)*	**27**<br>*23(s):4(d)*	**15**
			Ox **6**	**42**<br>*37(s):5(d)*	**30**<br>*25(s):5(d)*	**26**
			BA **7**	**0**	-	-
37	7	CjX183-D WT	2-PCA **1**	**87**<br>*24(s):63(d)*	-	**46**
			2-PCA **2**	**60**<br>*34(s):26(d)*	-	**20**
			2-PCA **3**	**81**<br>*50(s):31(d)*	-	**41**<br>*29(s):11(d)*
			TA4C **4**	**20**	-	**4**
			2-EBA **5**	**100**	-	**85**
			Ox **6**	**2**	-	-
			BA **7**	**0**	-	-
22	6	RNase A	2-PCA **1**	**81**	**71**	**51**
			2-PCA **2**	**95**	**77**	**56**
			2-PCA **3**	**88**	**75**	**62**
			TA4C **4**	**63**	**46**	**21**
			2-EBA **5**	**49**<br>*39(s):11(d)*	**42**<br>*34(s):8(d)*	**50**<br>*41(s):9(d)*
			Ox **6**	**0**	-	-
			BA **7**	**4**	**0**	-
22	6	Myoglobin	2-PCA **1**	**35**	**33**	**32**
			2-PCA **2**	**23**	**15**	**9**
			2-PCA **3**	**35**	**34**	**19**
			TA4C **4**	**0**	-	-
			2-EBA **5**	**48**<br>*29(s):14(d):4(t)*	**47**<br>*34(s):10(d):4(t)*	**50**<br>*39(s):11(d)*
			Ox **6**	**42**<br>*37(s):5(d)*	**31**	**30**
			BA **7**	**0**	-	-
22	6	CjX183-D WT	2-PCA **1**	**87**<br>*24(s):63(d)*	-	**69**<br>*24(s):45(d)*
			2-PCA **2**	**60**<br>*34(s):26(d)*	-	**24**<br>*17(s):7(d)*
			2-PCA **3**	**81**<br>*50(s):31(d)*	-	**44**<br>*15(s):28(d)*
			TA4C **4**	**20**	-	**0**
			2-EBA **5**	**100**	-	**86**
			Ox **6**	**2**	-	-

			BA **7**	**0**	-	-
22	8	RNase A	2-PCA **1**	**81**	**71**	**53**
			2-PCA **2**	**95**	**78**	**42**
			2-PCA **3**	**88**	**74**	**53**
			TA4C **4**	**63**	**38**	**21**
			2-EBA **5**	**49**<br>*39(s):11(d)*	**53**<br>*42(s):11(d)*	**48**<br>*38(s):10(d)*
			Ox **6**	**0**	-	-
			BA **7**	**4**	**0**	-
22	8	Myoglobin	2-PCA **1**	**35**	**34**	**31**
			2-PCA **2**	**23**	**17**	**9**
			2-PCA **3**	**35**	**34**	**27**
			TA4C **4**	**0**	-	-
			2-EBA **5**	**48**<br>*29(s):14(d):4(t)*	**38**<br>*27(s):5(d):5(t)*	**30**
			Ox **6**	**42**<br>*37(s):5(d)*	**33**<br>*28(s):5(d)*	**35**<br>*30(s):5(d)*
			BA **7**	**0**	-	-
22	8	CjX183-D WT	2-PCA **1**	**87**<br>*24(s):63(d)*	-	**65**<br>*27(s):39(d)*
			2-PCA **2**	**60**<br>*34(s):26(d)*	-	**25**<br>*18(s):6(d)*
			2-PCA **3**	**81**<br>*50(s):31(d)*	-	**44**<br>*18(s):26(d)*
			TA4C **4**	**20**	-	**2**
			2-EBA **5**	**100**	-	**88**
			Ox **6**	**2**	-	-
			BA **7**	**0**	-	-

Table S4. Stability of protein conjugates to pH and temperature over time. Modification of RNase A, myoglobin, and CjX183-D WT with compounds **1-7** under conditions outlined in **General Procedure A**, and conversions observed over time upon exposure to a range of conditions (pH 6-8, 4-37 °C).

4.2 Stability to competitive dipeptide

Stability of protein conjugates upon addition of DiAla as a competitor

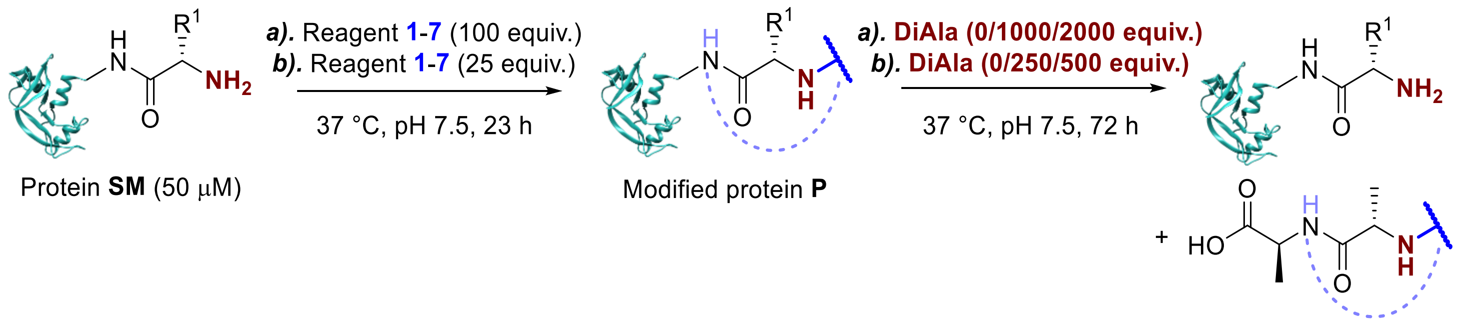

a). For the modification of RNase A with 100 equiv. reagent: RNase A was modified with compounds **1-7** according to **General Procedure A**, on a 200 µL scale. For compound **7**, 5 equiv. of sodium cyanoborohydride in 10 µL of water was also added, and compound **7** was added as a more concentrated solution (90 µL, 11 mM) to keep the total volume constant. The reaction was then split into 3 aliquots (65 µL). To these, were added a different concentration of L-alanyl- L-alanine (DiAla; 26 µL; 0, 125, or 250 mM; 0, 1000, or 2000 equiv.) in sodium phosphate buffer (50 mM pH 7.5). The reactions were incubated at 37 °C for 72 h with agitation (1000 rpm). Aliqouts (10 µL) were taken from each mixture at *t* = 24 and 72 h, diluted with water (30 µL), and analysed by LC-MS.

b). For the modification of RNase A with 25 equiv. reagent: Reactions were run as described above, but using 25 equiv. of reagents **1-7** and 0, 250 or 500 equiv. DiAla. LC-MS analysis was carried out at *t* = 72 h.

a). For the modification of CjX183-D WT with 100 equiv. reagent: CjX183-D WT was modified with compounds **1-7** according to **General Procedure A**, on a 150 µL scale. For compound **7**, 5 equiv. of sodium cyanoborohydride in 7.5 µL of water was also added, and compound **7** was added as a more concentrated solution (67.5 µL, 11 mM) to keep the total volume constant. The reaction was then split into 3 aliquots (40 µL). To these, were added a different concentration of L-alanyl- L-alanine (DiAla; 16 µL; 0, 125, or 250 mM; 0, 1000, or 2000 equiv.) in sodium phosphate buffer (50 mM pH 7.5). The reactions were incubated at 37 °C for 72 h with agitation (1000 rpm). Aliqouts (10 µL) were taken from each mixture at *t* = 24 and 72 h, diluted with water (30 µL), and analysed by LC-MS.

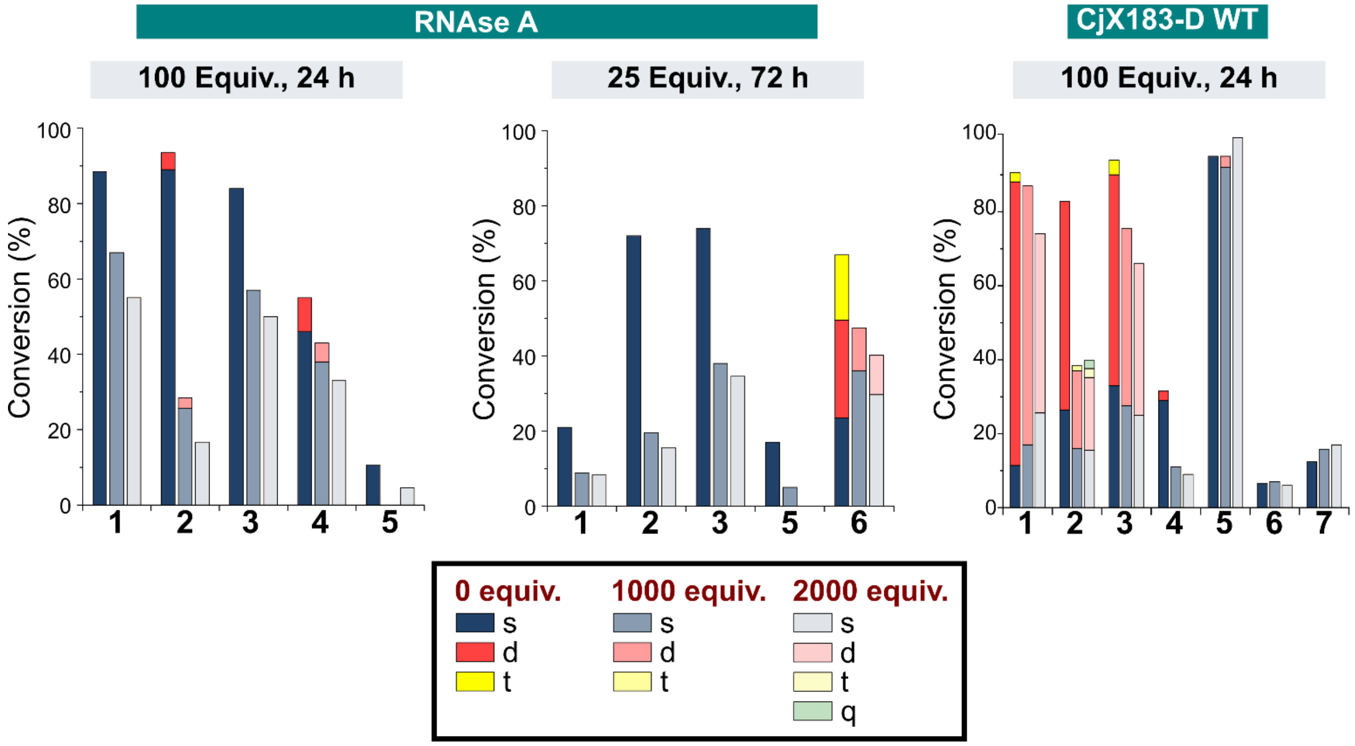

Figure S9. Stability of protein conjugates to competitor over time. Modification of RNase A and CjX183-D WT with compounds **1-7** (100 or 25 equiv.) under conditions outlined in **General Procedure A**, and conversions observed over time upon addition of DiAla as a competitor (0-2000 equiv.).

Protein	No. equiv. protein modifier	Compound	Time /h	**Conversion (%)**		
				0 equiv. DiAla	1000 equiv. DiAla	2000 equiv. DiAla
RNase A	100	2-PCA **1**	24	**88**	**78**	**73**
			72	**88**	**67**	**55**
		2-PCA **2**	24	**91**	**65**	**59**
			72	**94**<br>*89(s):5(d)*	**28**<br>*26(s):3(d)*	**17**
		2-PCA **3**	24	**81**	**77**	**69**
			72	**84**	**57**	**50**
		TA4C **4**	24	**67**	**40**	**35**
			72	**68**	**11**	**9**
		Ox **6**	24	**52**<br>*39(s):13(d)*	**41**<br>*36(s):5(d)*	**38**<br>*34(s):4(d)*
			72	**56**<br>*46(s):9(d)*	**44**<br>*39(s):5(d)*	**34**
		BA **7**	24	**8**	**5**	**5**
			72	**11**	**0**	**5**
RNase A	25	2-PCA **1**	72	**21**	**9**<br>*(250 equiv. DiAla)*	**8**<br>*(500 equiv. DiAla)*
		2-PCA **2**	72	**72**	**20**<br>*(250 equiv. DiAla)*	**15**<br>*(500 equiv. DiAla)*
		2-PCA **3**	72	**74**	**38**<br>*(250 equiv. DiAla)*	**35**<br>*(500 equiv. DiAla)*
		TA4C **4**	72	**17**	**5**<br>*(250 equiv. DiAla)*	**0**<br>*(500 equiv. DiAla)*
		2-EBA **5**	72	**67**<br>*24(s):26(d):17(t)*	**48**<br>*36(s):12(d)*<br>*(250 equiv. DiAla)*	**40**<br>*30(s):10(d)*<br>*(500 equiv. DiAla)*
		Ox **6**	72	**0**	**0**	**0**
		BA **7**	72	**0**	**0**	**0**
CjX183-D WT	100	2-PCA **1**	24	**91**<br>*11(s):77(d):3(t)*	**88**<br>*17(s):70(d)*	**74**<br>*26(s):49(d)*
			72	**94**<br>*4(s):87(d):3(t)*	**62**<br>*37(s):25(d)*	**65**<br>*41(s):24(d)*
		2-PCA **2**	24	**83**<br>*26(s):56(d)*	**39**<br>*16(s):21(d):1(t)*	**40**<br>*15(s):20(d):2(t):2(q)*
			72	**93**<br>*16(s):78(d)*	**23**<br>*16(s):5(d):2(t)*	**20**<br>*14(s):6(d)*
		2-PCA **3**	24	**93**<br>*33(s):57(d):4(t)*	**76**<br>*28(s):48(d)*	**66**<br>*25(s):41(d)*
			72	**95**<br>*14(s):75(d):6(t)*	**57**<br>*19(s):38(d)*	**48**<br>*19(s):29(d)*
		TA4C **4**	24	**32**<br>*29(s):3(d)*	**11**	**9**
			72	**30**<br>*28(s):2(d)*	**3**	**0**
		2-EBA **5**	24	**95**	**96**	**100**

			92(s):3(d)	
	72	**98**	**95**	**94**
Ox **6**	24	**7**	**7**	**6**
	72	**14**	**4**	**0**
BA **7**	24	**12**	**16**	**17**
	72	**18**	**10**	**15**

Table S5. Stability of protein conjugates to competitor over time. Modification of RNase A and CjX183-D WT with compounds **1-7** under conditions outlined in **General Procedure A**, and conversions observed over time upon addition of DiAla as a competitor (0-2000 equiv.).

5. Kinetic studies

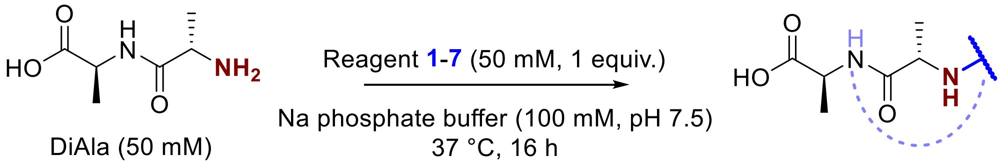

A solution of DiAla (150 µL, 100 mM, 15 µmol, 1 equiv.) was added to solutions of reagents **1-7** (150 µL, 100 mM, 15 µmol, 1 equiv.), both in deuterated sodium phosphate buffer (100 mM, pH 7.5). Due to discrepancies during the weighing out of small masses of **1-7**, exact concentrations were determined by ^{1}H NMR spectroscopy (2-PCA 1, 45 mM; 2-PCA **2,** 39 mM; 2-PCA **3,** 46 mM; TA4C **4,** 33 mM; 2-EBA **5,** 17 mM; Ox **6**, 36 mM, BA **7**, 50 mM) in deuterated Na phosphate buffer (100 mM, pH 7.5). The reactions were incubated at 37 °C for 16 h and conversion was followed by ^{1}H NMR spectroscopy at 30 min intervals.

Data were fit to a second order irreversible or reversible kinetic model in Copasi 4.34.251. k_1 and k_{-1} were estimated using the evolutionary programming method built into the software, with 200 generations and a population size of 20. Parameters were restricted within the confines of: k_1 10^{-11}-10^{5} M^{-1} s^{-1}; k_{-1} 10^{-11}-10^{5} M^{-1} s^{-1}.

2-PCAs 1-3

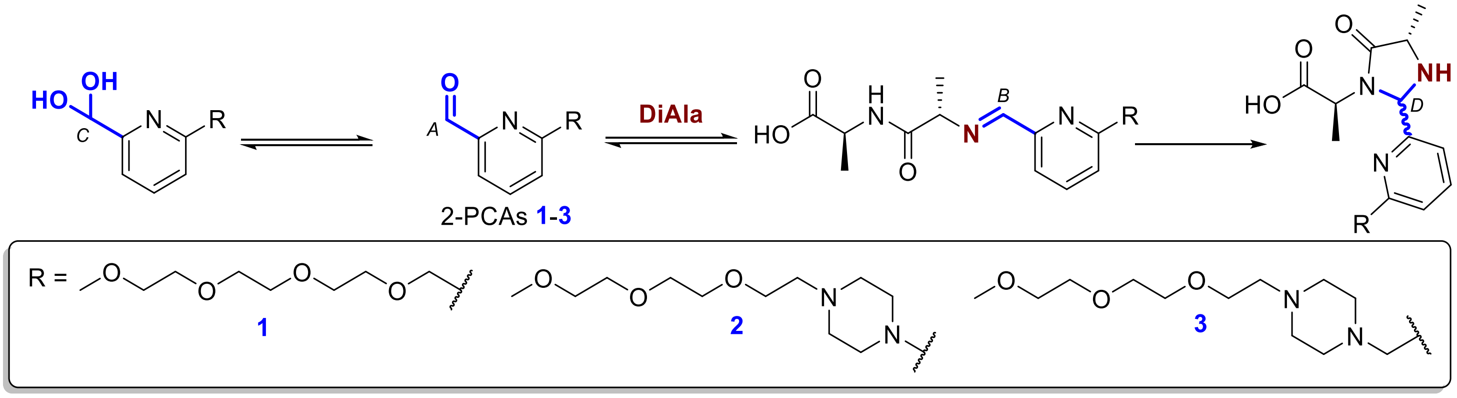

<u>2-PCA 1</u> (t = 16 h)

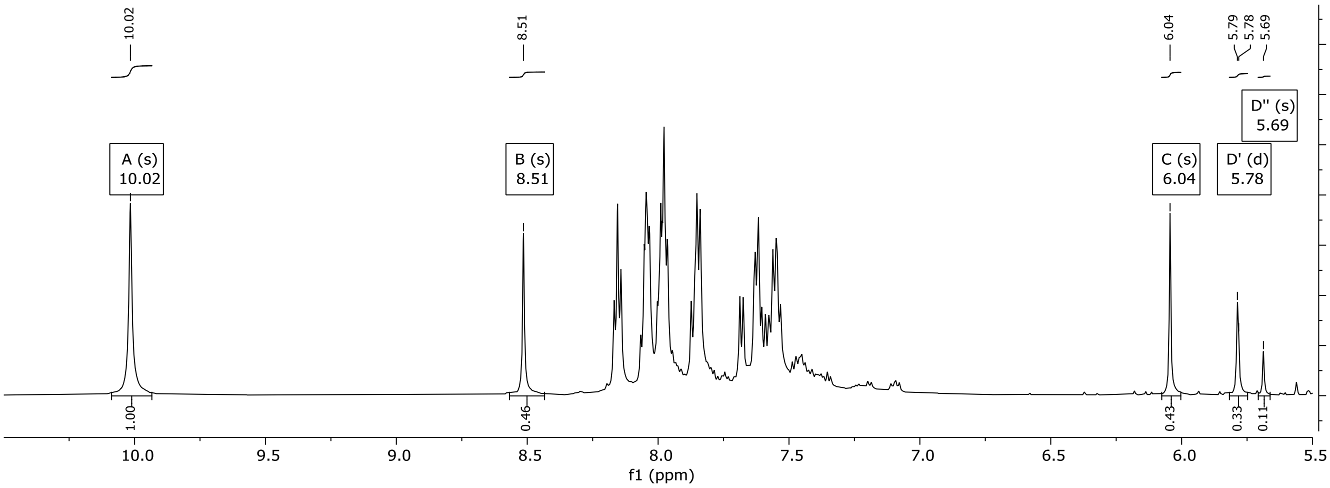

2-PCA 2 (t = 16 h)

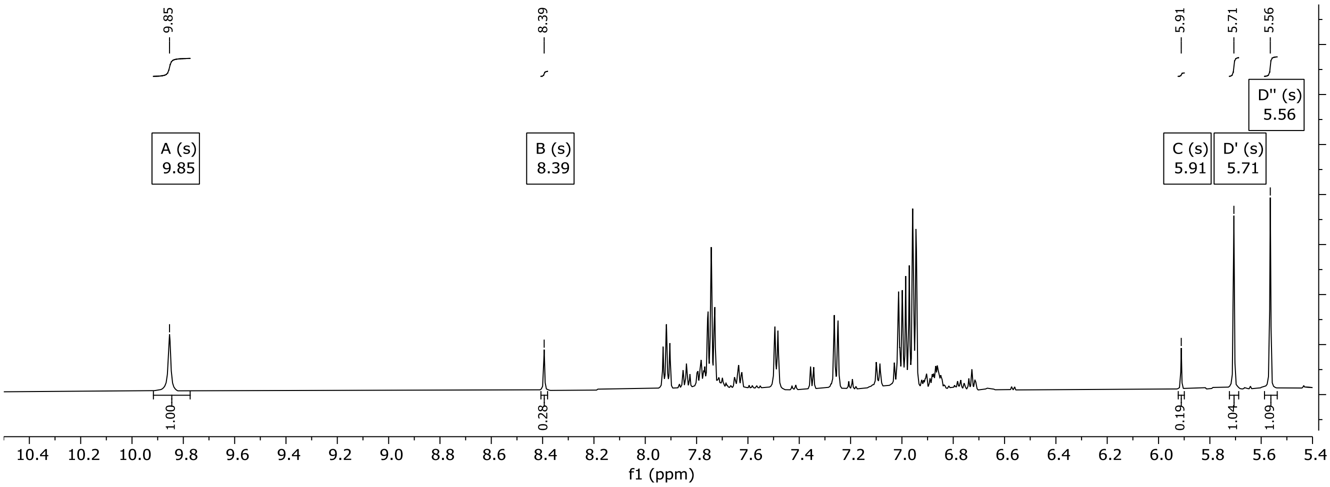

2-PCA 3 (t = 16 h)

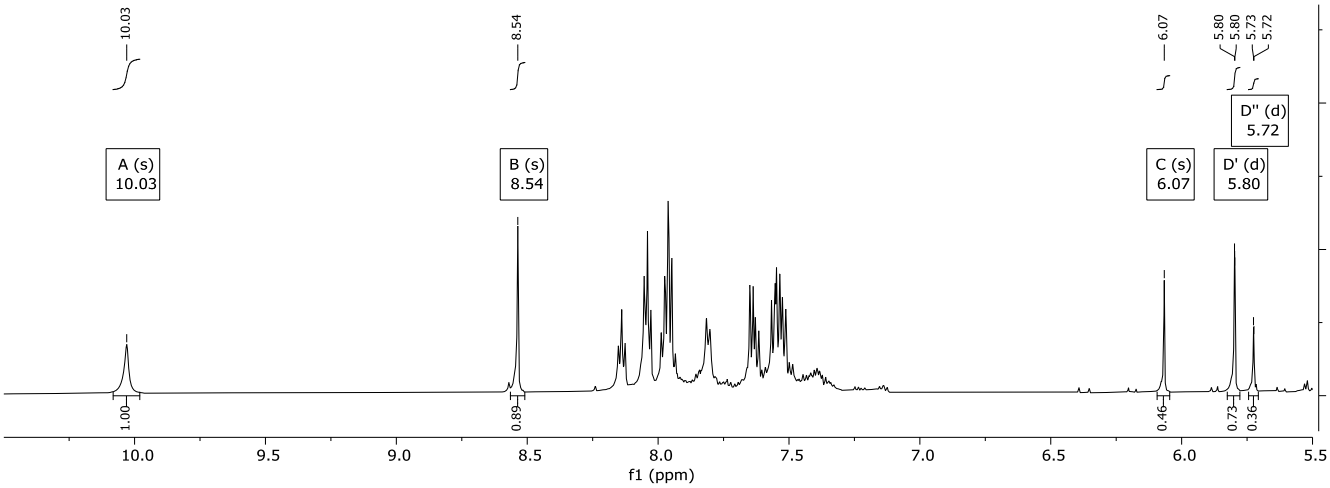

TA4C 4 (t = 16 h)

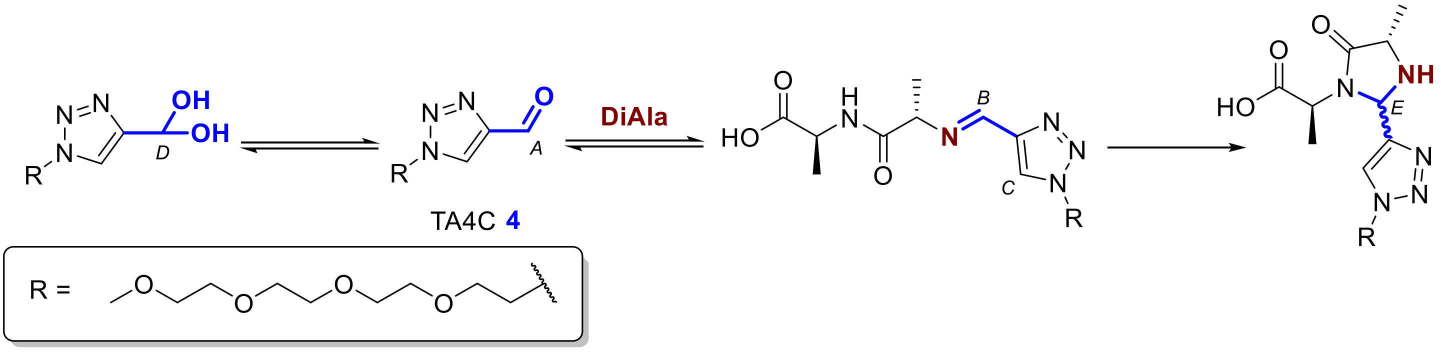

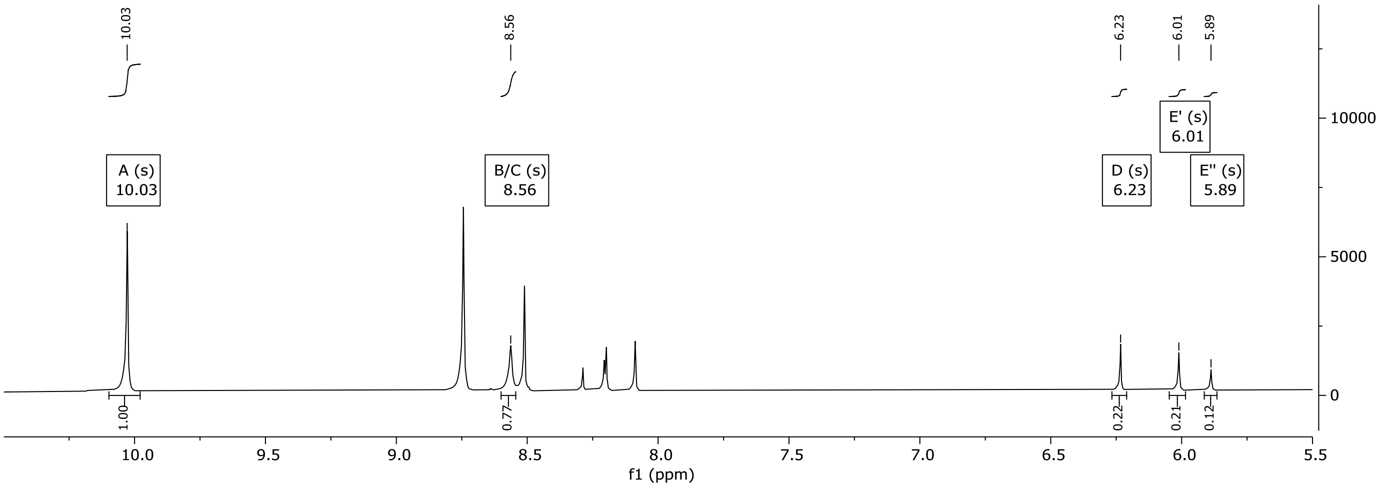

2-EBA 5 (t = 16 h)

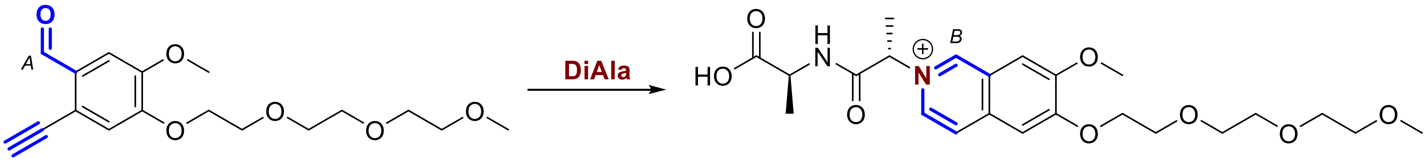

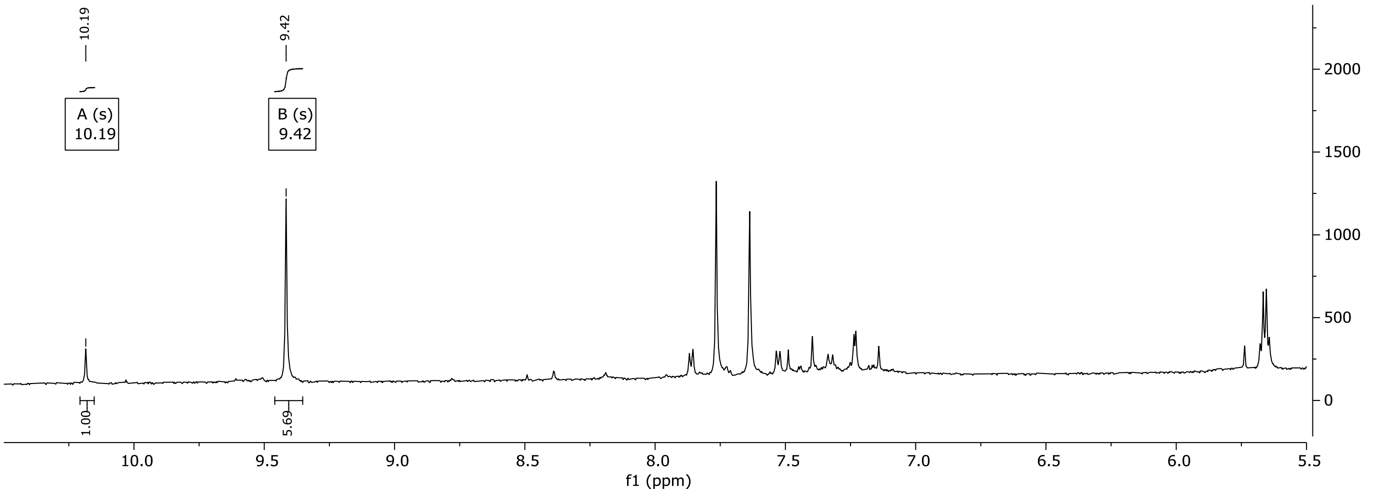

Ox 6 (t = 16 h)

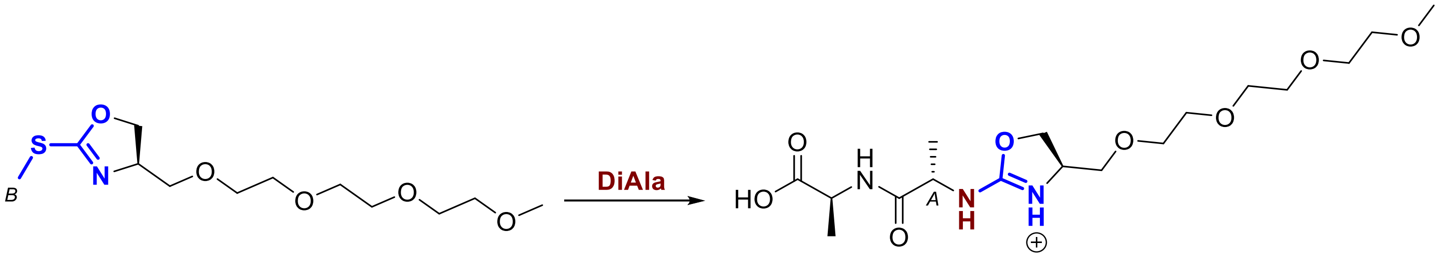

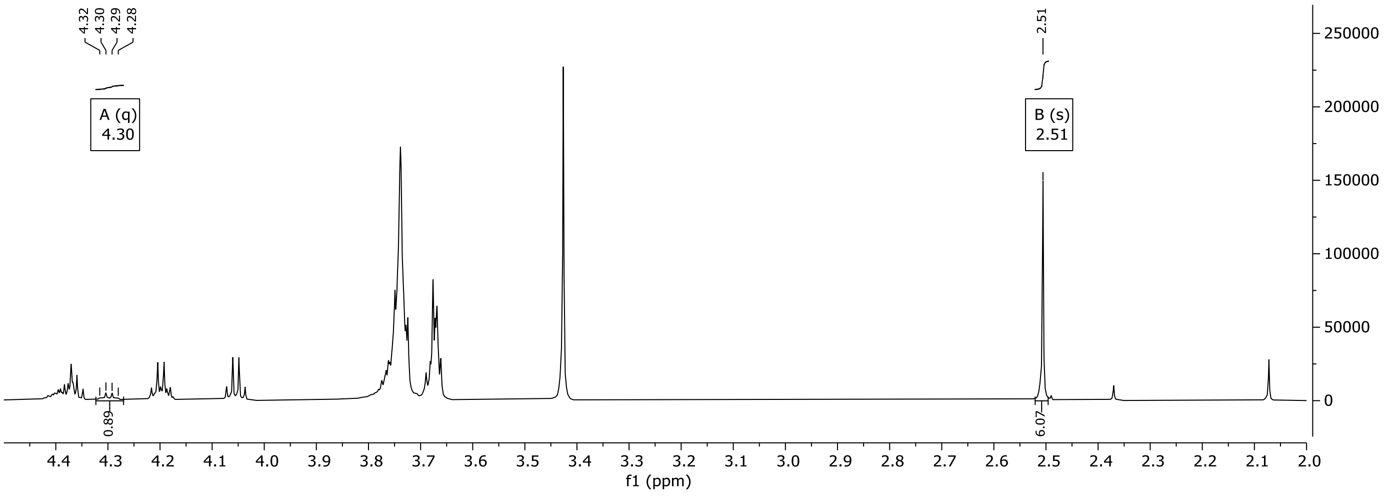

BA 7 (t = 1 h)

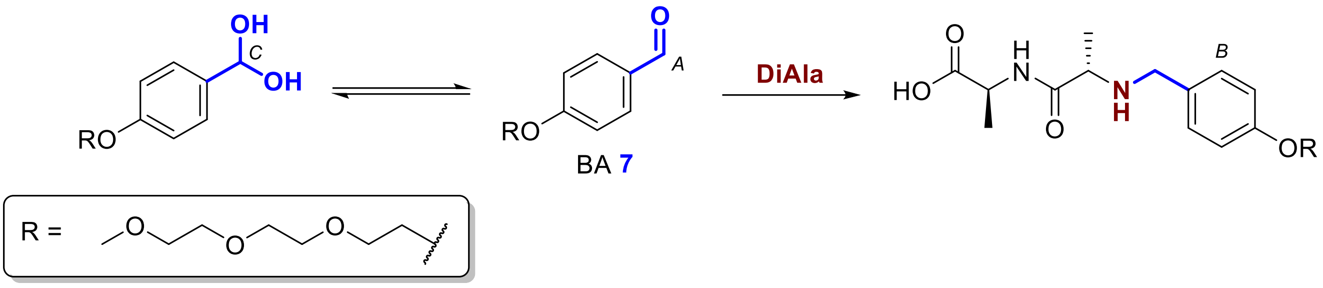

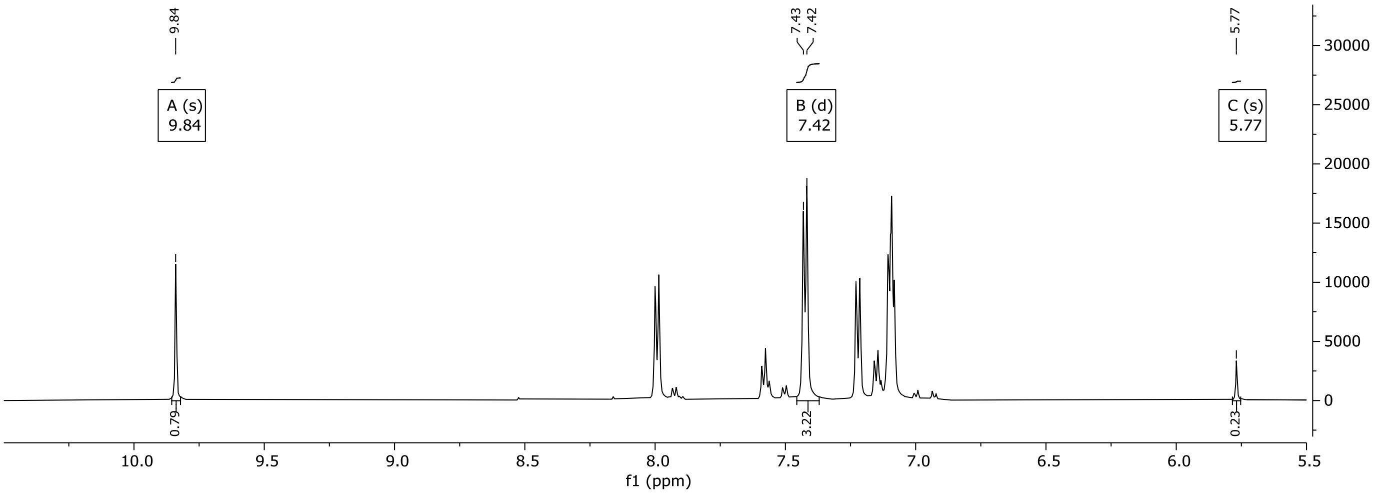

Figure S10. Representative ^{1}H NMR spectra of kinetics experiments showing the diagnostic signals used for kinetic modelling.

References

1. Wang, R. *et al.* Traceless cross-linker for photocleavable bioconjugation. *Bioconjug. Chem.* **23**, 705–713 (2012).

2. Zaia, J., Annan, R. S. & Biemann, K. The correct molecular weight of myoglobin, a common calibrant for mass spectrometry. *Rapid Commun. Mass Spectrom.* **6**, 32–36 (1992).

3. Macdonald, J. I., Munch, H. K., Moore, T. & Francis, M. B. One-step site-specific modification of native proteins with 2-pyridinecarboxyaldehydes. *Nat. Chem. Biol.* **11**, 326–331 (2015).

4. Kohmoto, S., Mori, E. & Kishikawa, K. Room-temperature discotic nematic liquid crystals over a wide temperature range: Alkali-metal-ion-induced phase transition from discotic nematic to columnar phases. *J. Am. Chem. Soc.* **129**, 13364–13365 (2007).

5. Denißen, M. *et al.* One-pot synthesis of a white-light emissive bichromophore operated by aggregation-induced dual emission (AIDE) and partial energy transfer. *Chem. Commun.* **56**, 7407–7410 (2020).

6. Wang, F., Tan, X., Lv, H. & Zhang, X. New Ruthenium Complexes Based on Tetradentate Bipyridine Ligands for Catalytic Hydrogenation of Esters. *Chem. - An Asian J.* **11**, 2103–2106 (2016).

7. Koo, B., Dolan, N. S., Wucherer, K., Munch, H. K. & Francis, M. B. Site-Selective Protein Immobilization on Polymeric Supports through N-Terminal Imidazolidinone Formation. *Biomacromolecules* **20**, 3933–3939 (2019).

8. Bartl, J., Reinke, L., Koch, M. & Kubik, S. Selective sensing of sulfate anions in water with cyclopeptide-decorated gold nanoparticles. *Chem. Commun.* **56**, 10457–10460 (2020).

9. Raj, M. & Tang, K. C. One Step Azolation strategy for site- and chemo-selective labeling of proteins with mass sensitive probes. *Angew. Chemie Int. Ed.* (2020) doi:10.1002/anie.202007608.

10. Angew Chem Int Ed - 2013 - Erbas-Cakmak - Cascading of Molecular Logic Gates for Advanced Functions A Self-Reporting .pdf.

11. Onoda, A., Inoue, N., Sumiyoshi, E. & Hayashi, T. Triazolecarbaldehyde Reagents for One-Step N-Terminal Protein Modification. *ChemBioChem* **21**, 1274–1278 (2020).

12. Deng, J. R. *et al.* N-Terminal selective modification of peptides and proteins using 2-ethynylbenzaldehydes. *Commun. Chem.* **3**, 1–9 (2020).

13. Chen, D., Disotuar, M. M., Xiong, X., Wang, Y. & Chou, D. H. C. Selective N-terminal functionalization of native peptides and proteins. *Chem. Sci.* **8**, 2717–2722 (2017).

^{1}H NMR (400 MHz, DMSO-d_6)

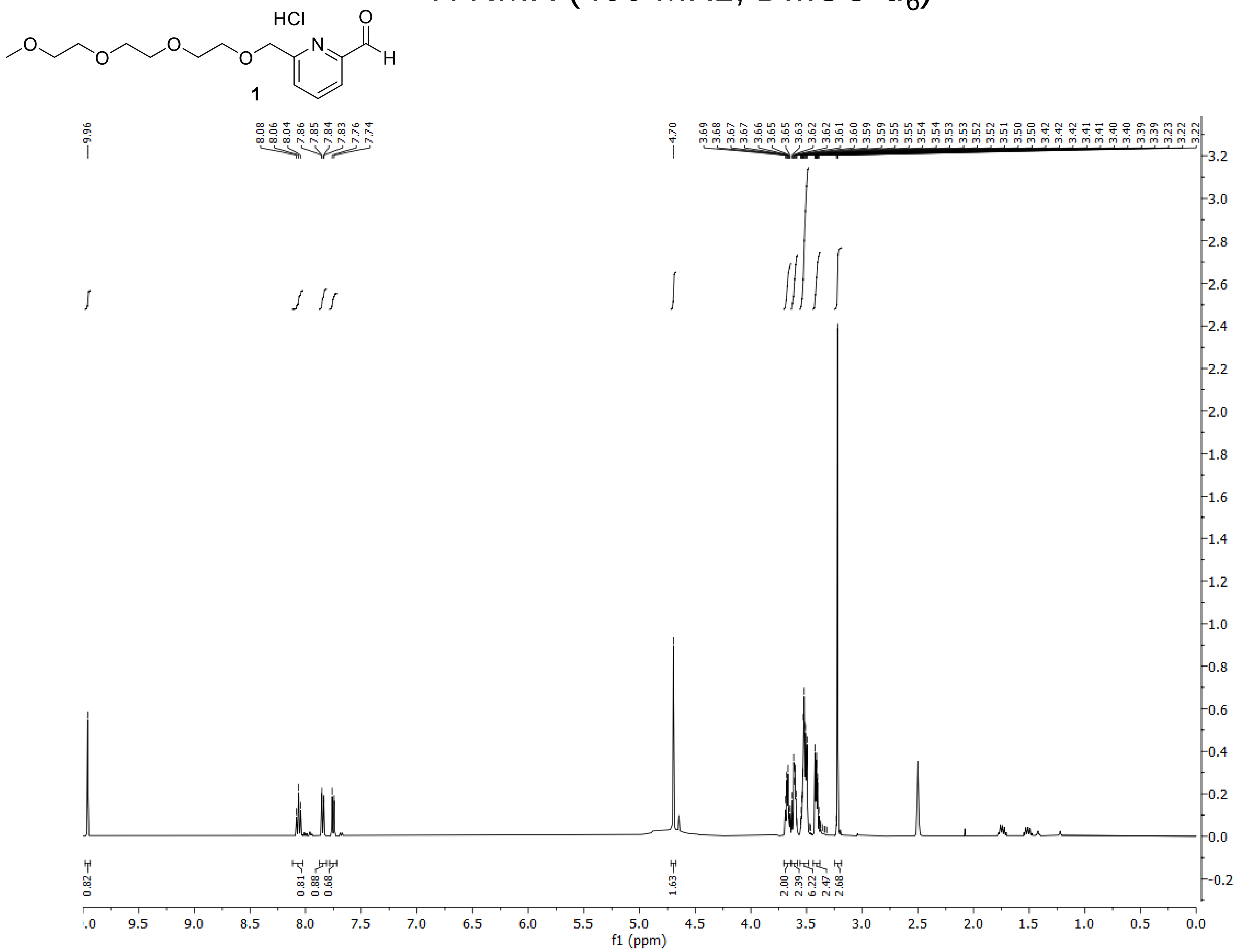

^{13}C NMR (101 MHz, DMSO-d_6)

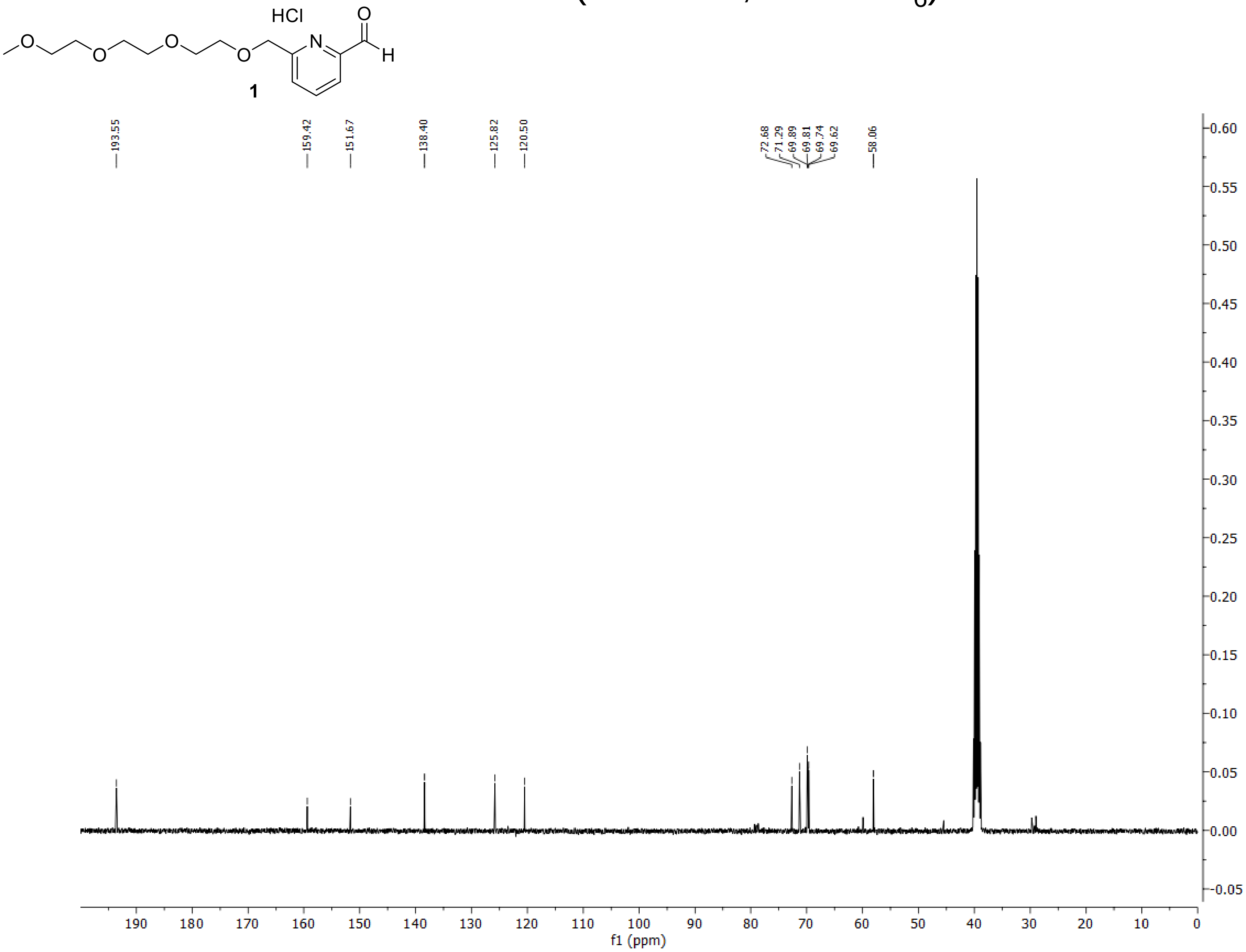

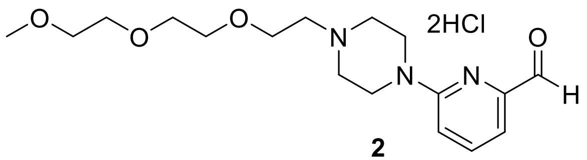

2

^{1}H NMR (500 MHz, DMSO-d_6)

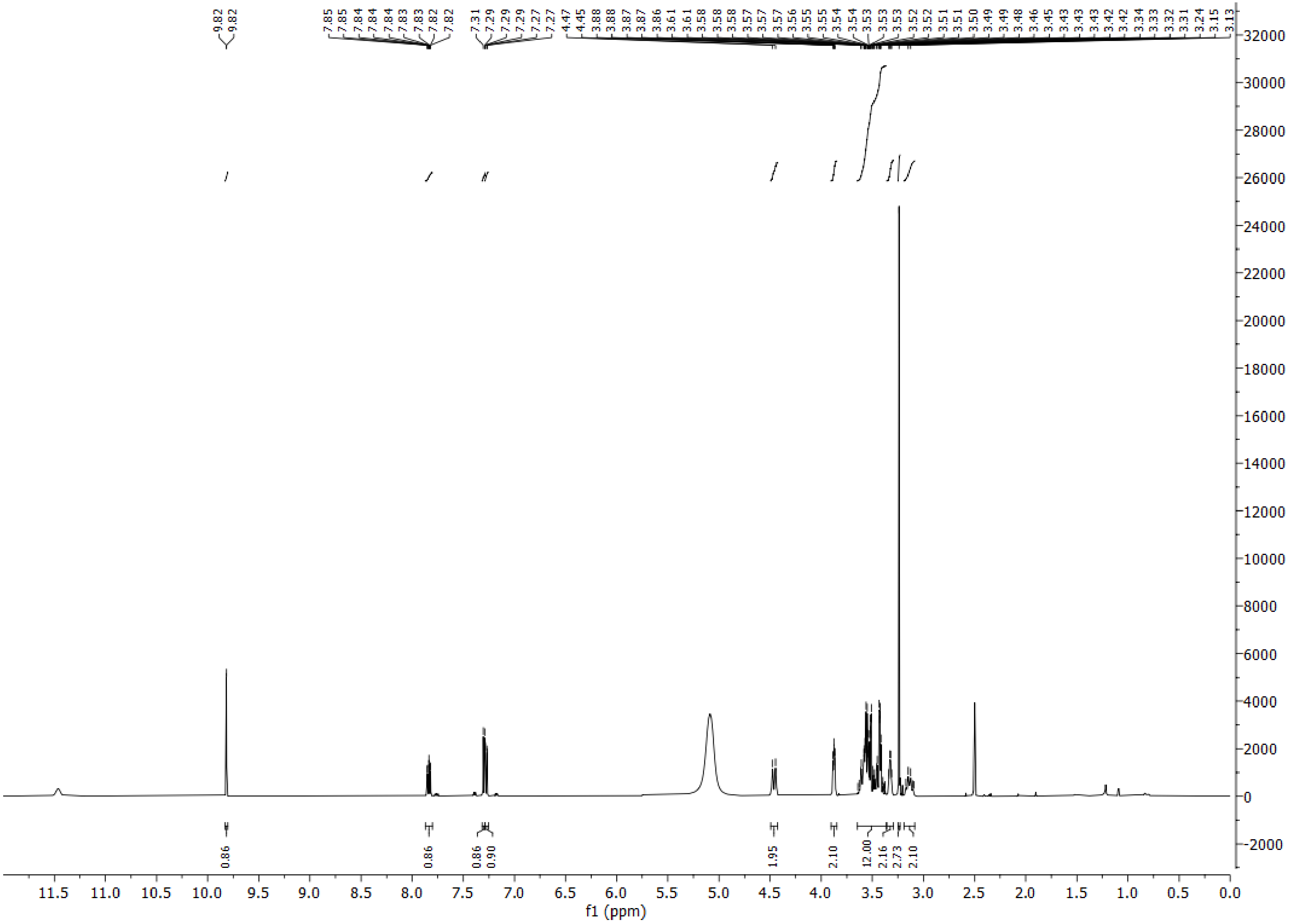

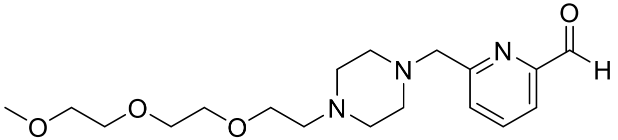

3

^1H NMR (400 MHz, $CDCl_3$)

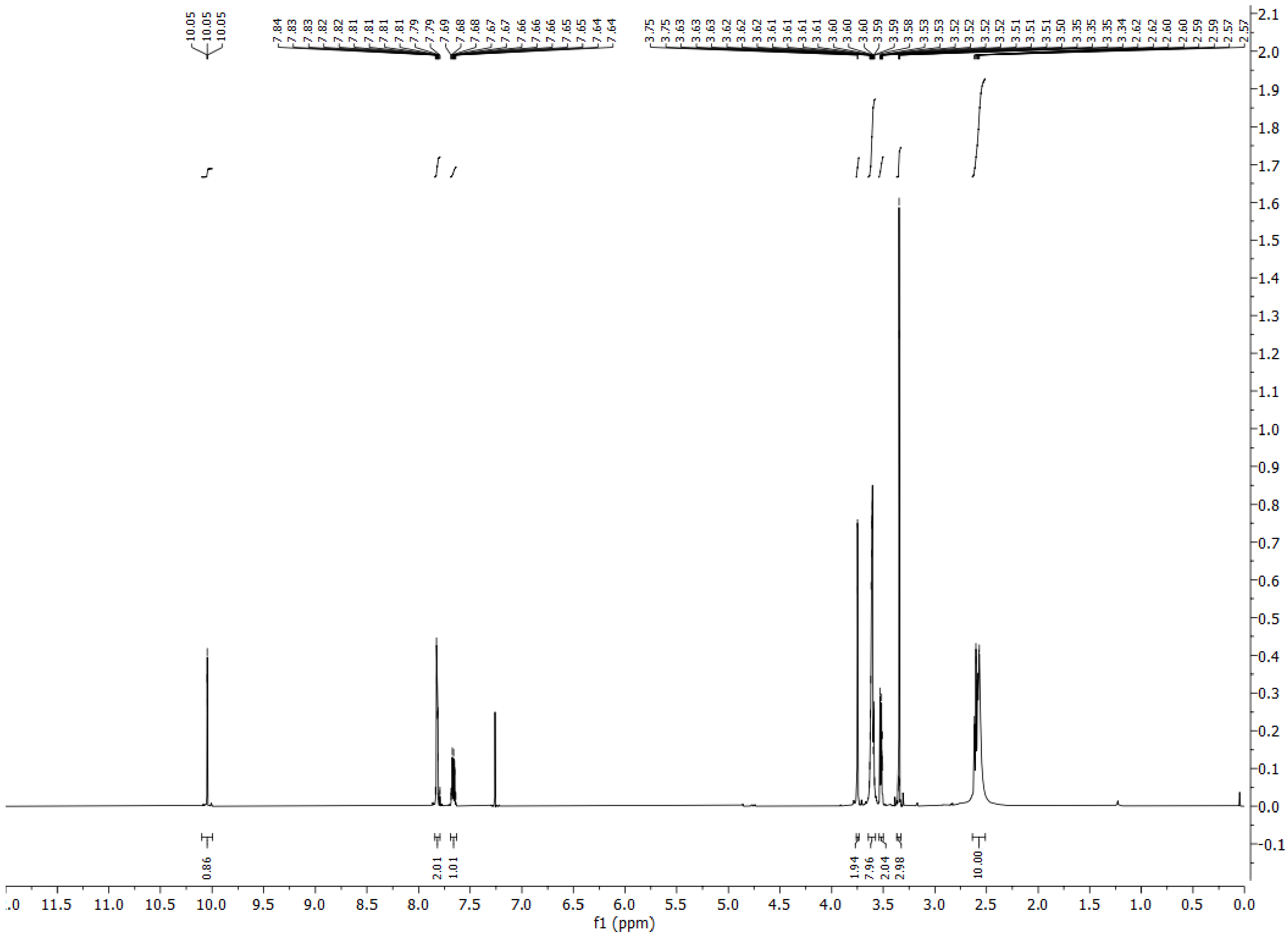

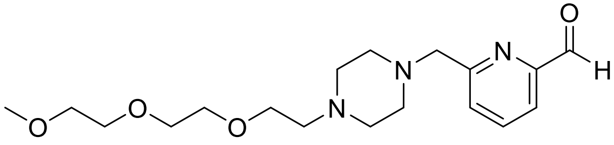

3

^{13}C NMR (101 MHz, $CDCl_3$)

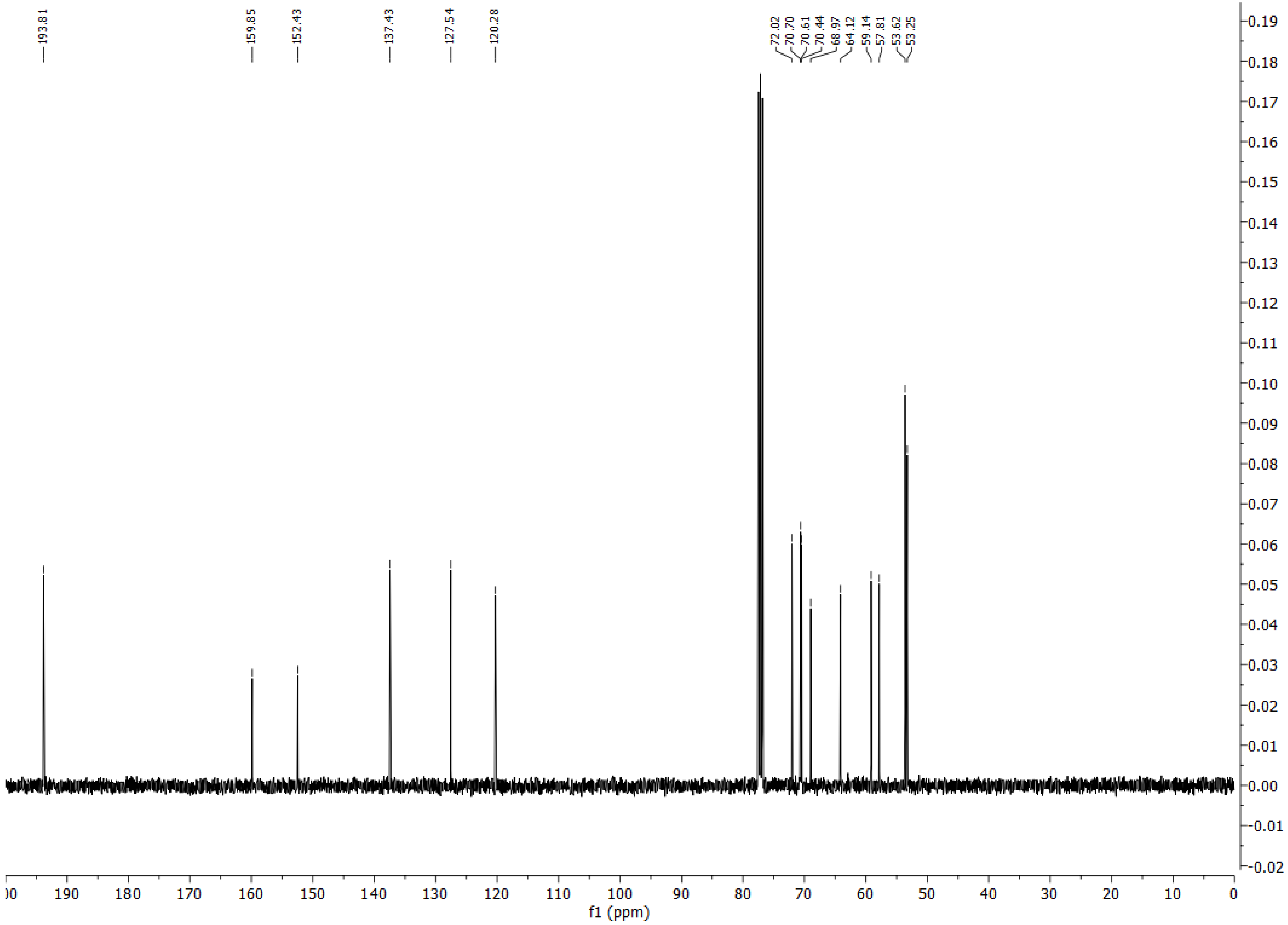

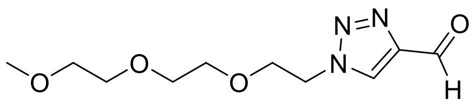

4

^{1}H NMR (300 MHz, $CDCl_3$)

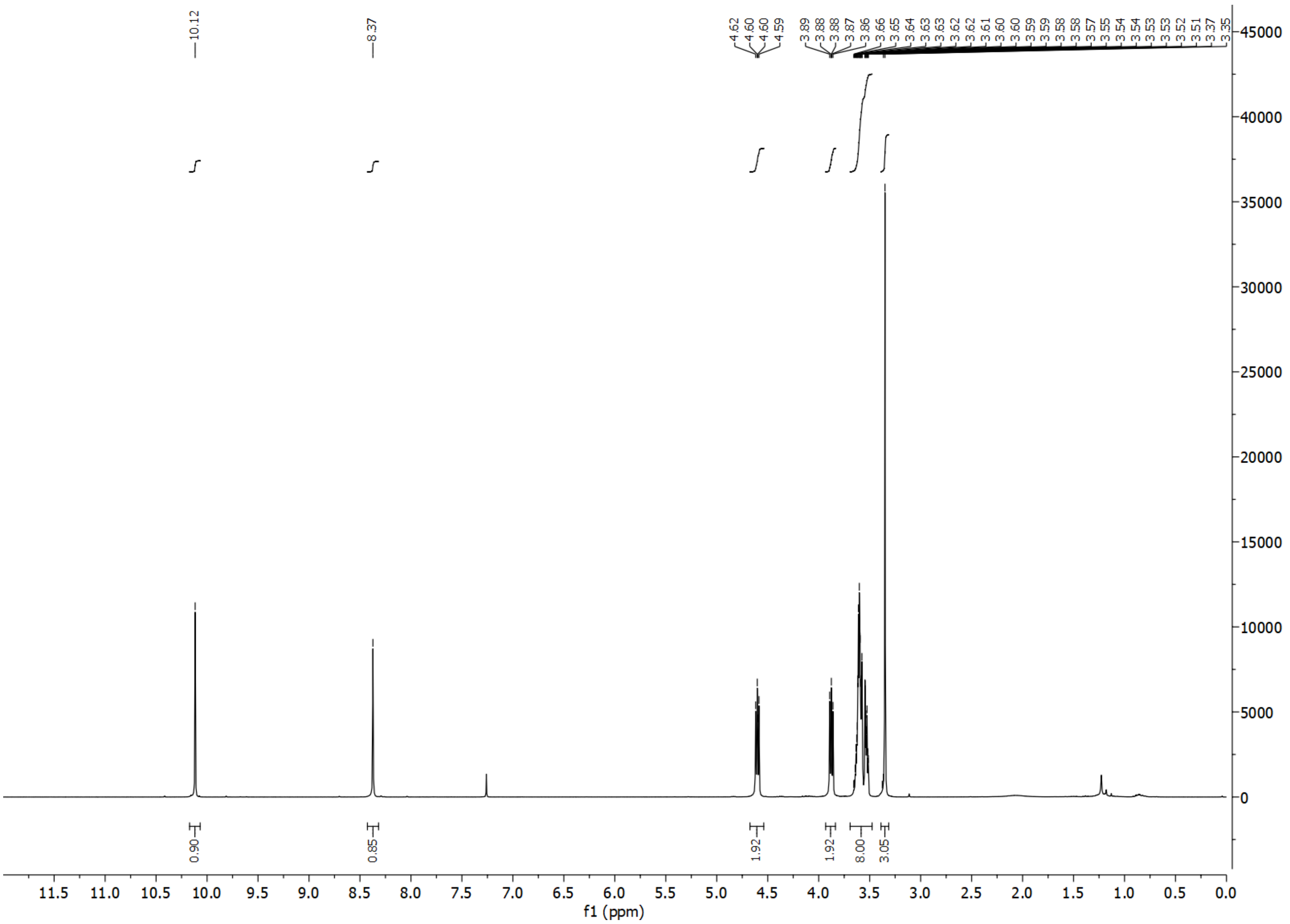

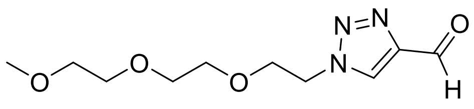

4

^{13}C NMR (75 MHz, $CDCl_3$)

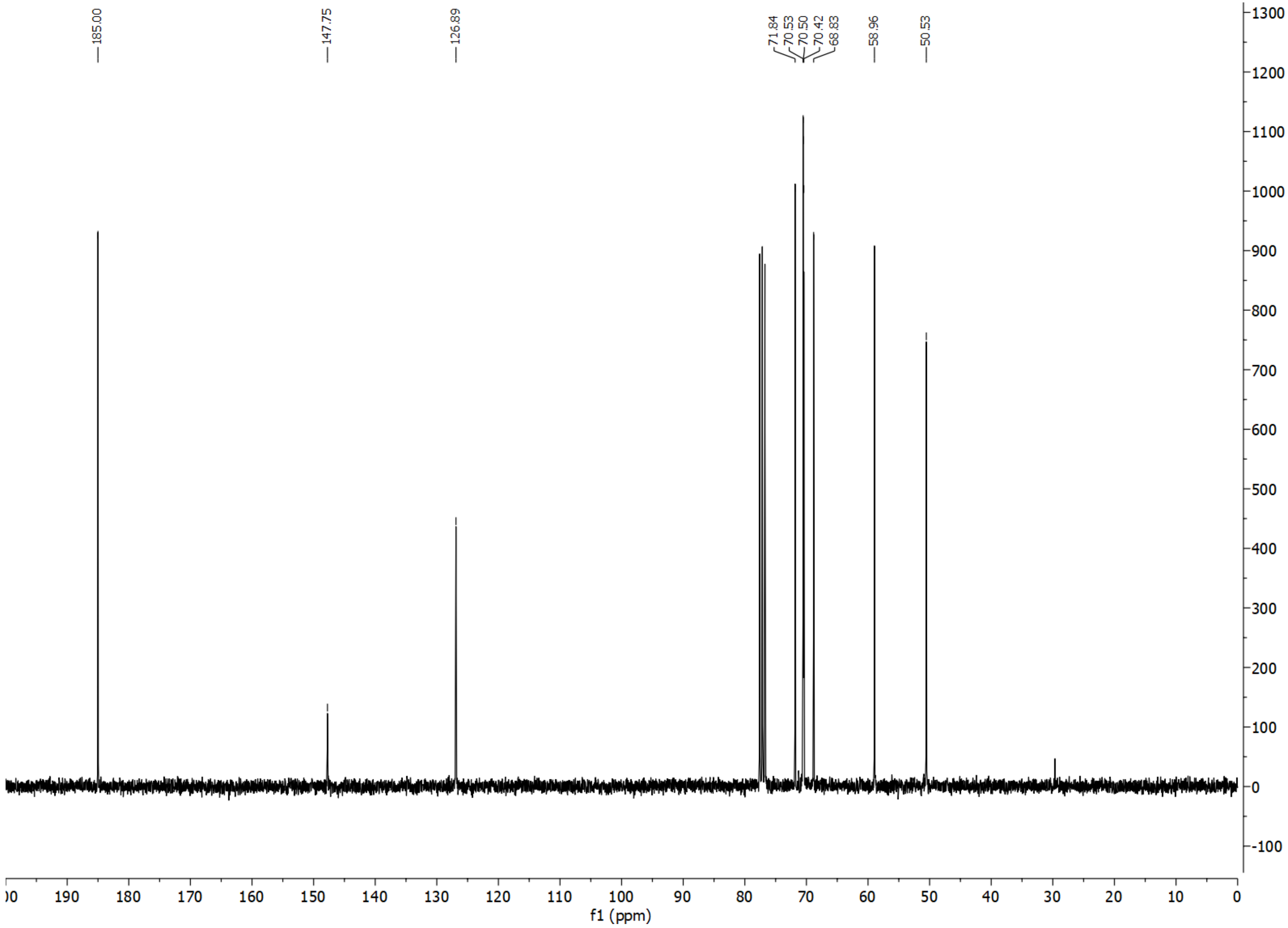

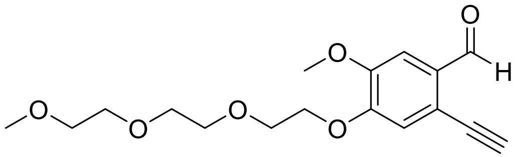

5

^{1}H NMR (300 MHz, CD_3OD)

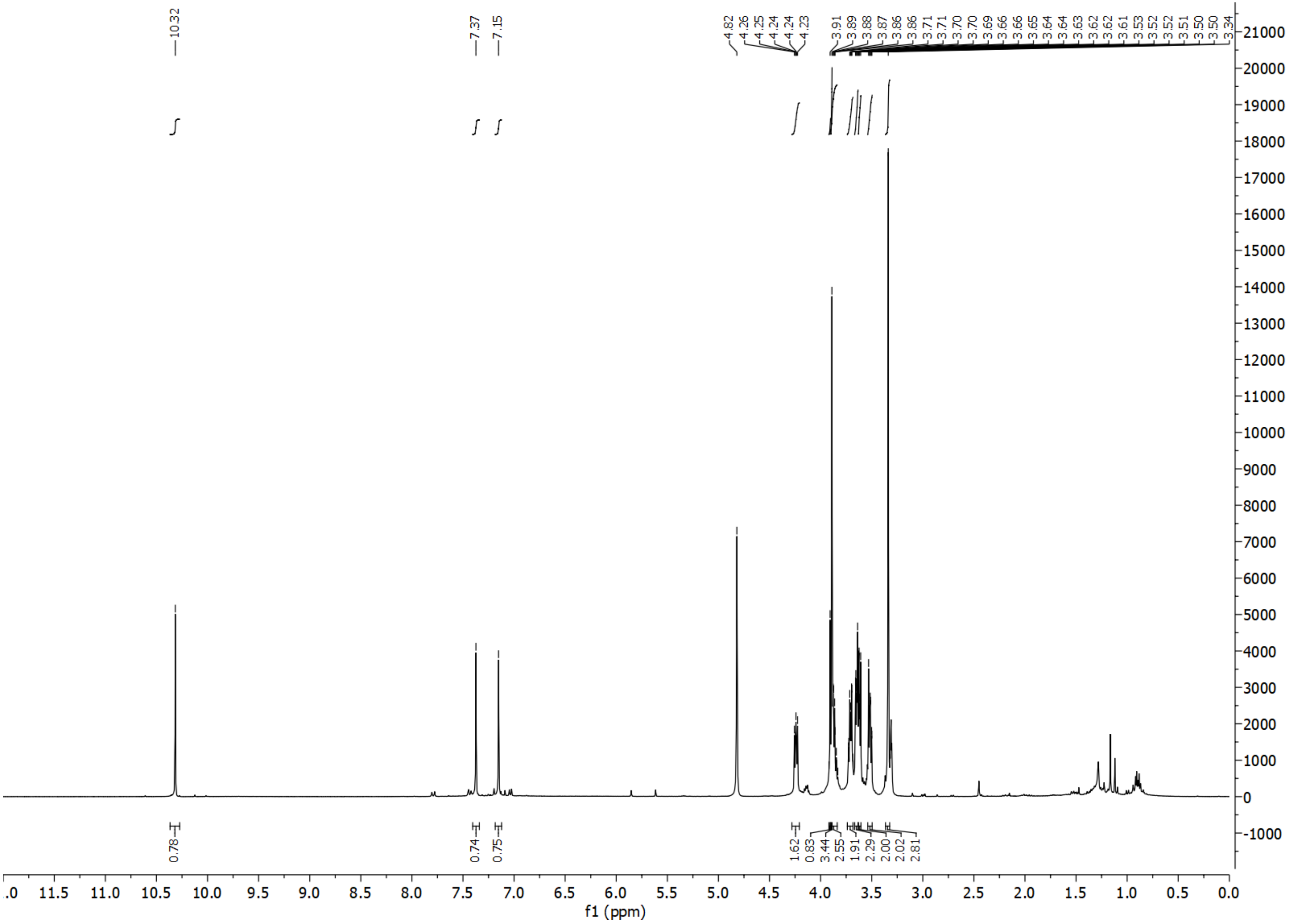

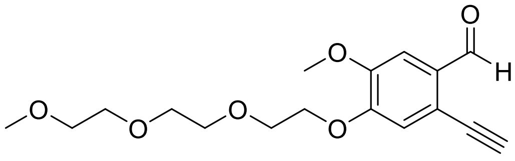

5

^{13}C NMR (75 MHz, CD_3OD)

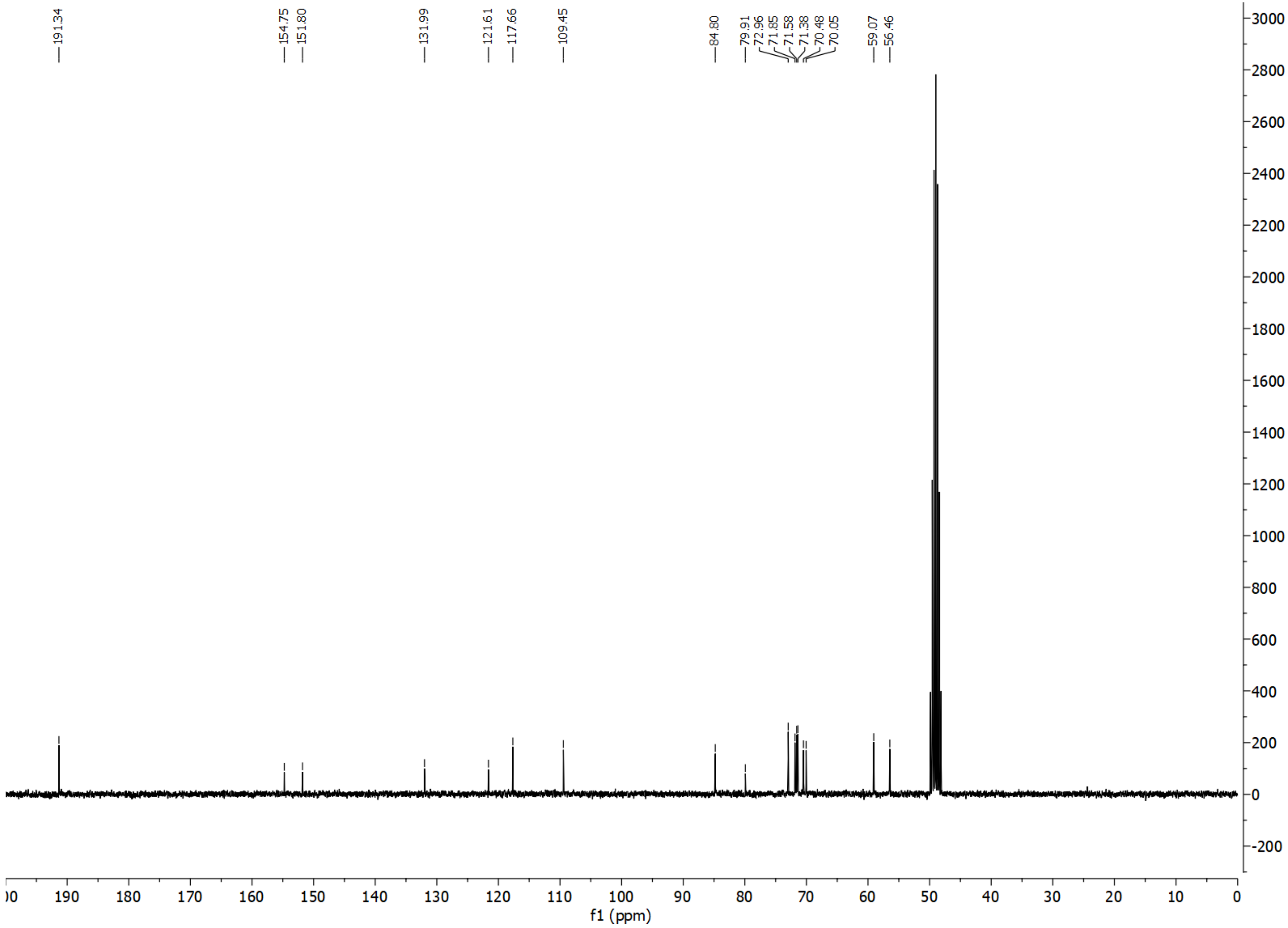

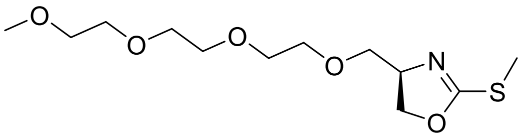

6

^1H NMR (300 MHz, $CDCl_3$)

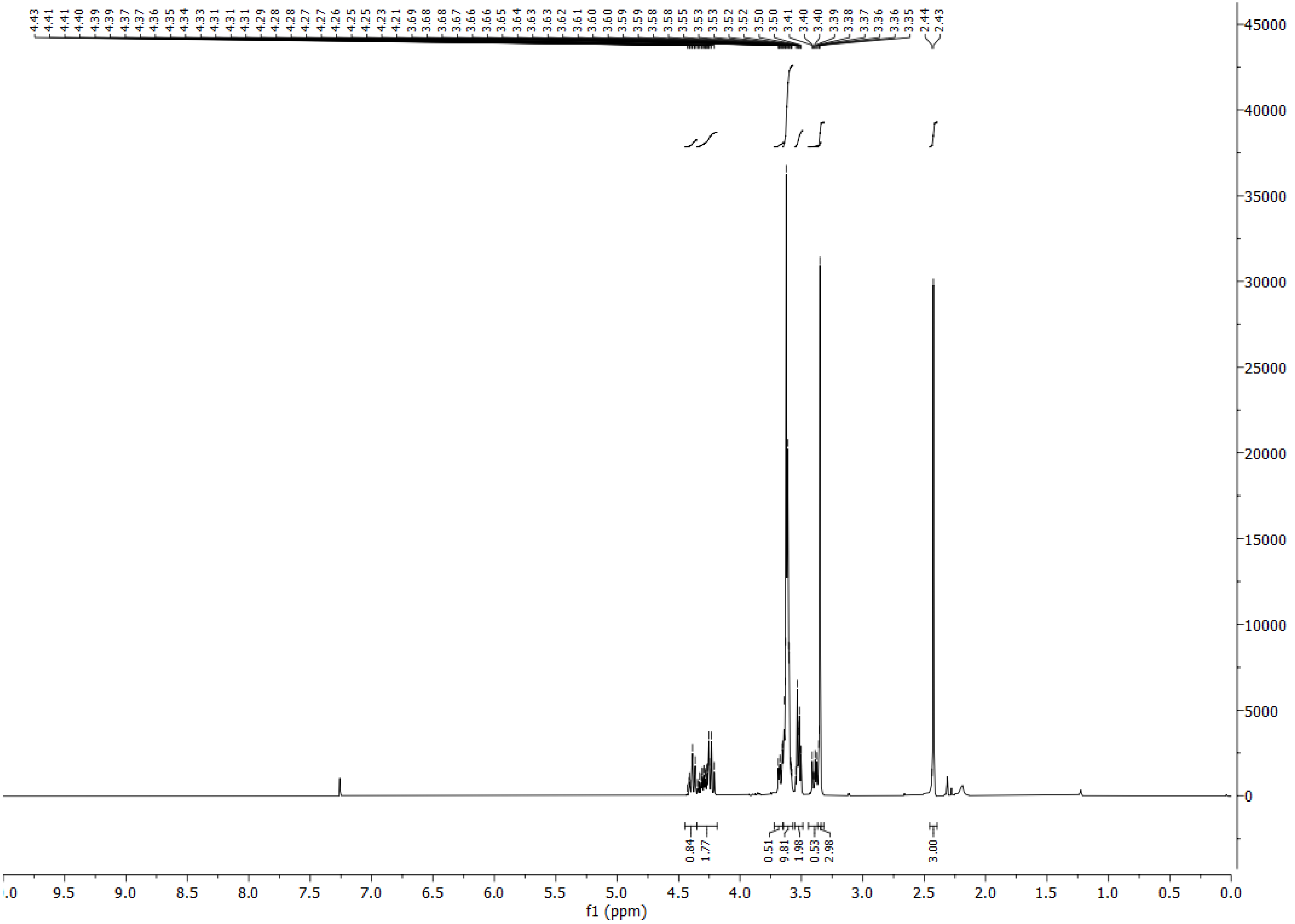

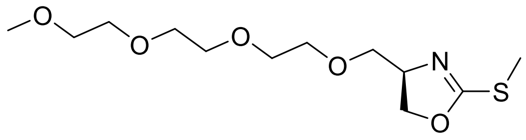

6

^{13}C NMR (75 MHz, $CDCl_3$)

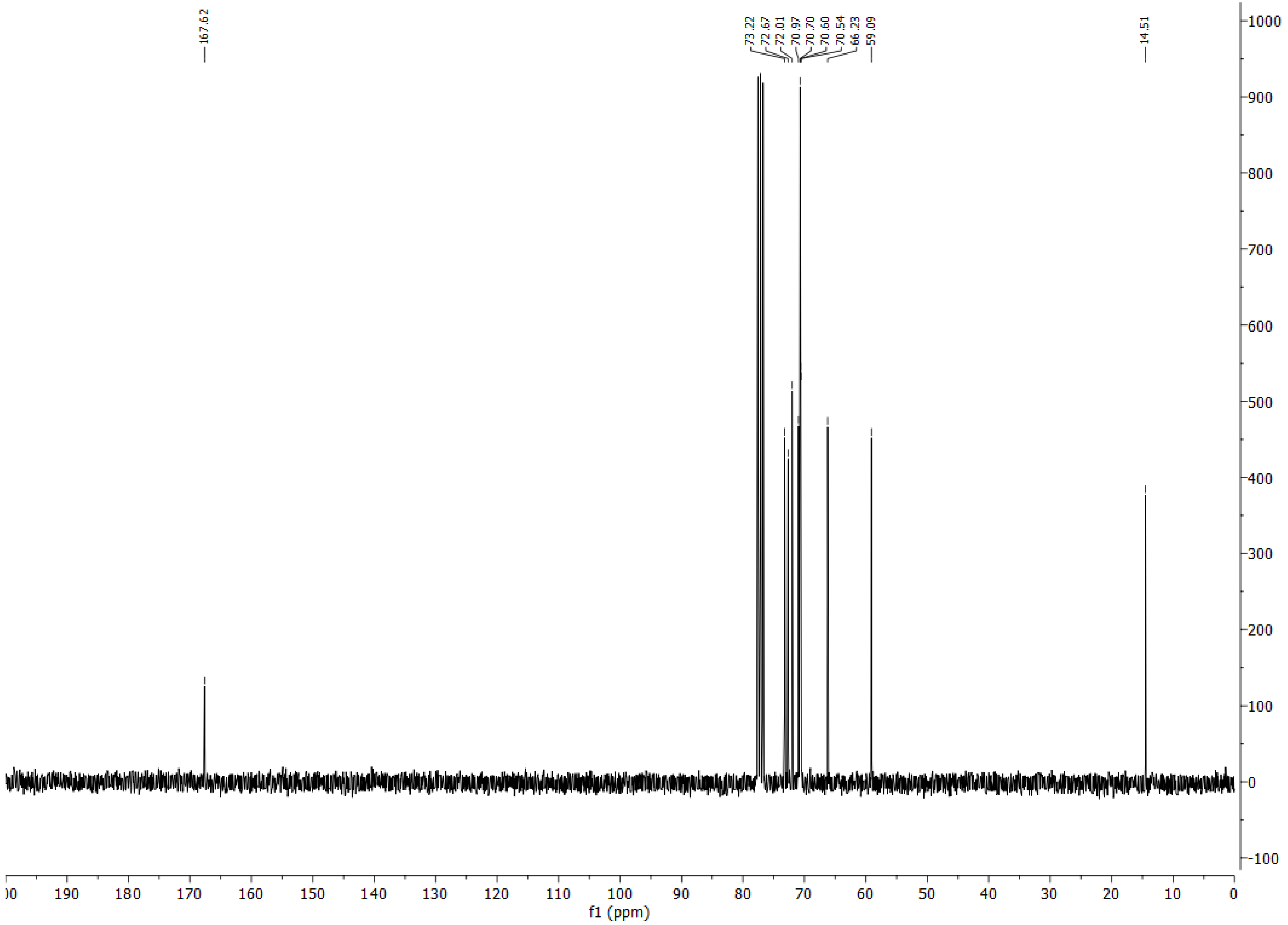

^1H NMR (400 MHz, $CDCl_3$)

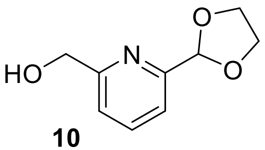

10

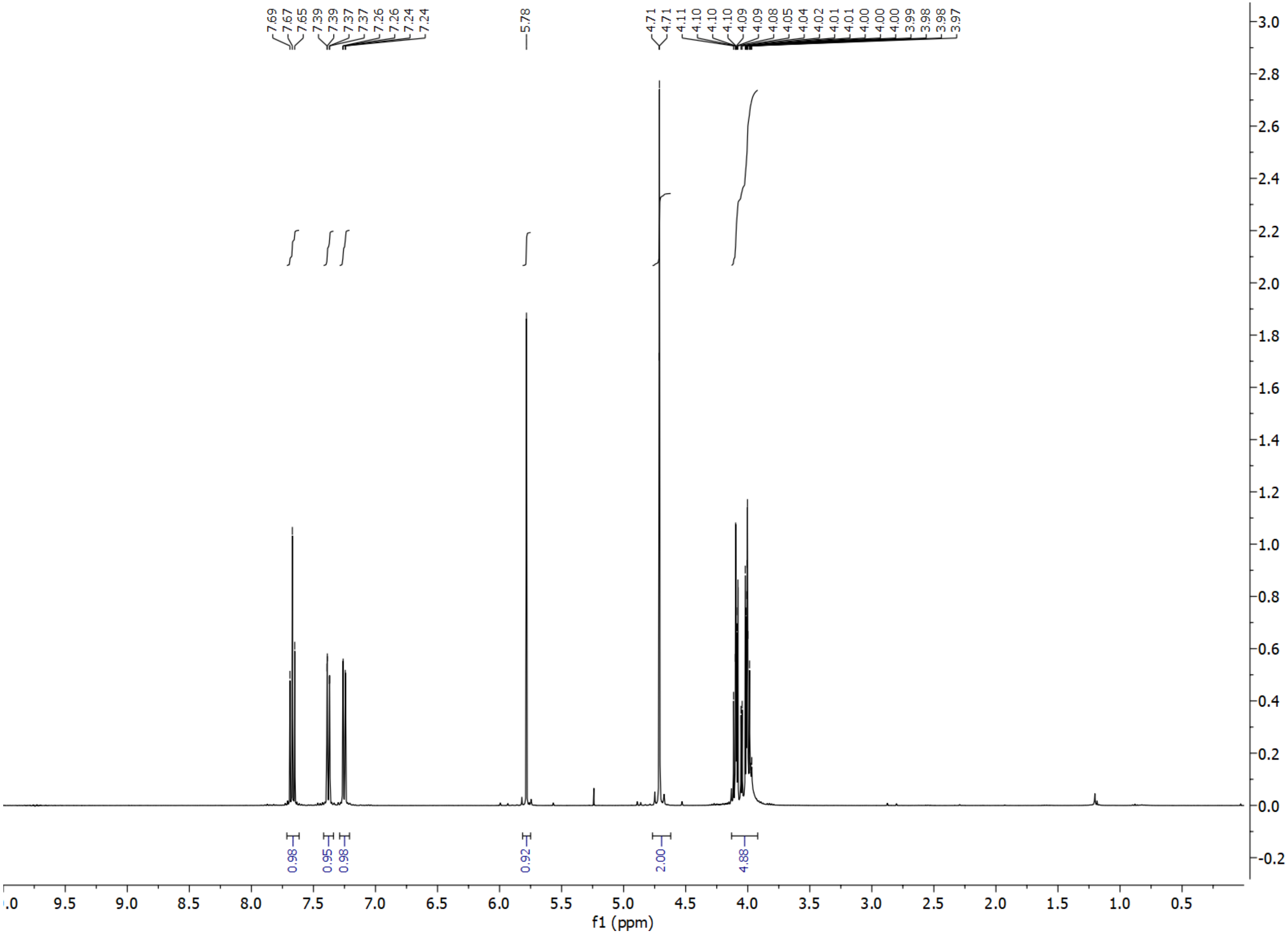

^{13}C NMR (101 MHz, $CDCl_3$)

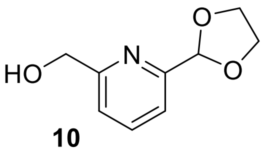

10

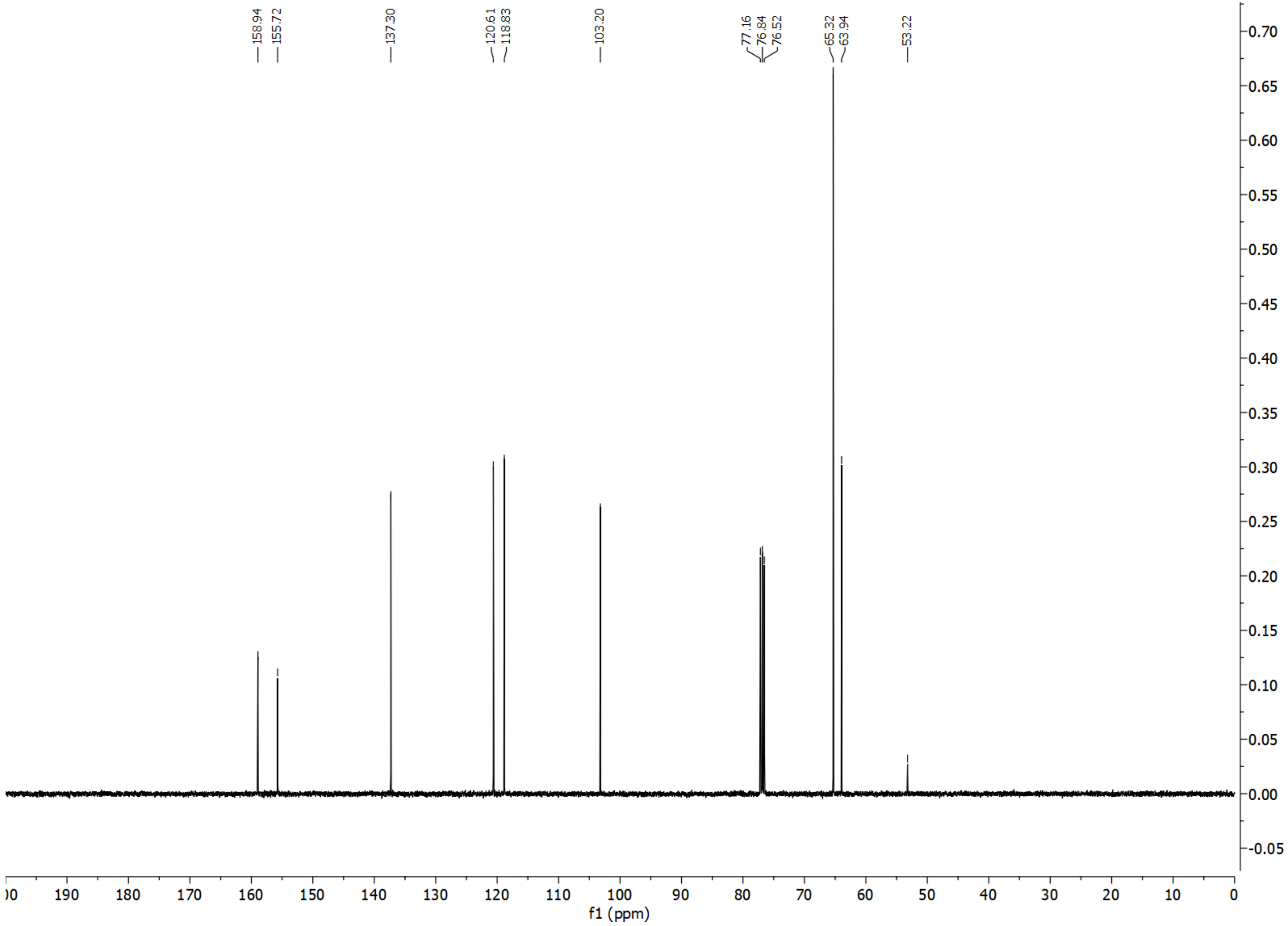

^{1}H NMR (300 MHz, $CDCl_3$)

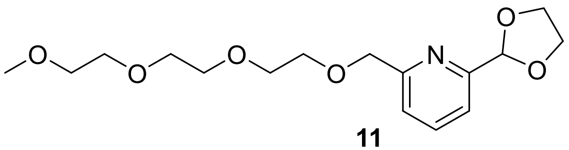

11

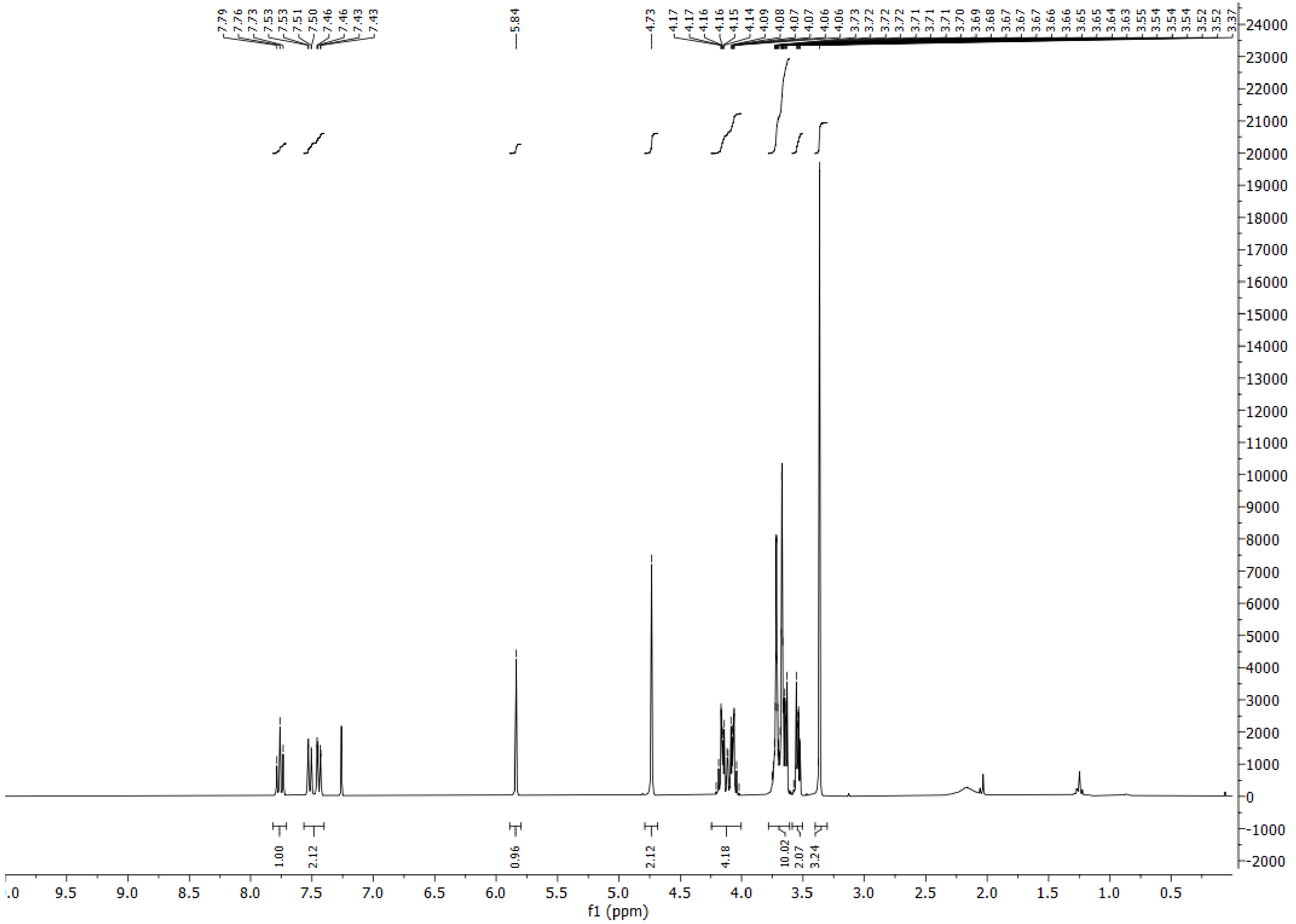

^{13}C NMR (75 MHz, $CDCl_3$)

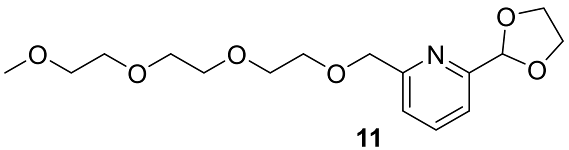

11

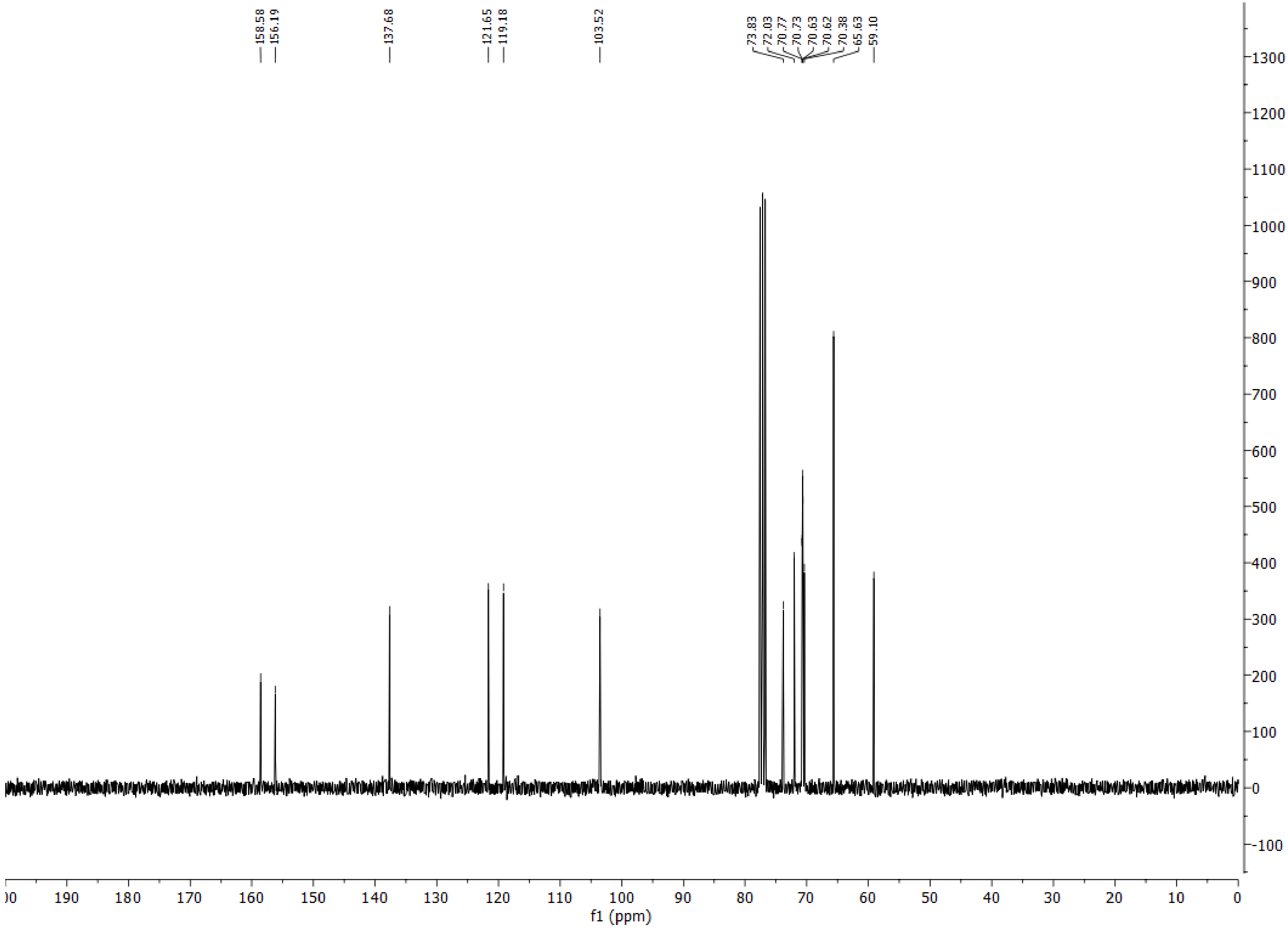

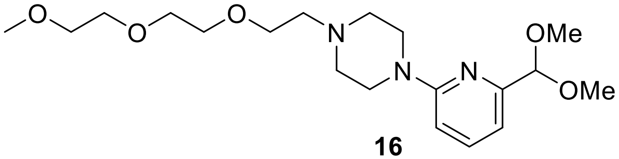

16

^{1}H NMR (300 MHz, $CDCl_3$)

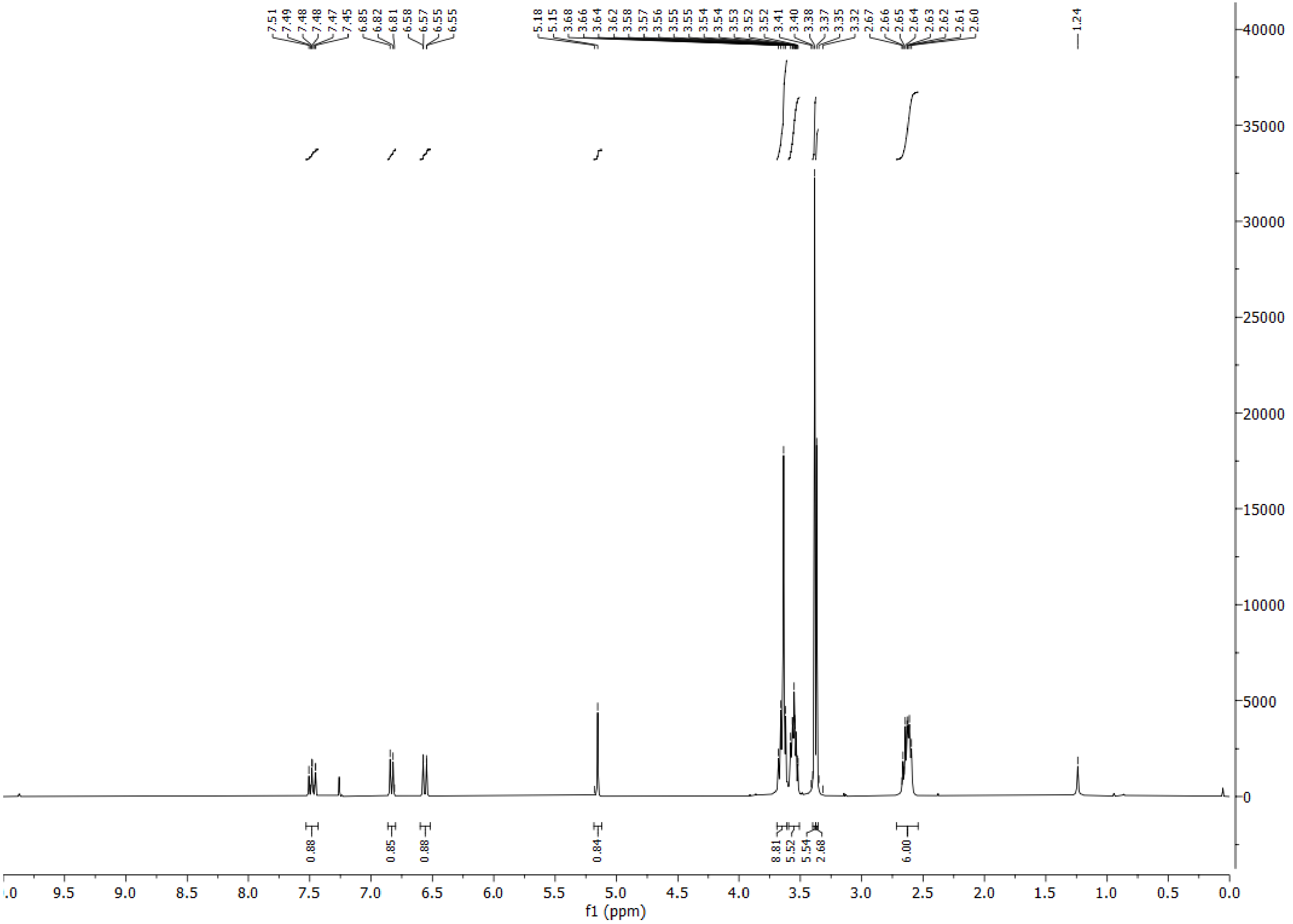

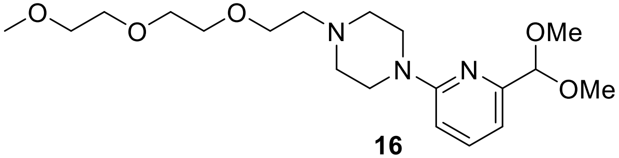

16

^{13}C NMR (75 MHz, $CDCl_3$)

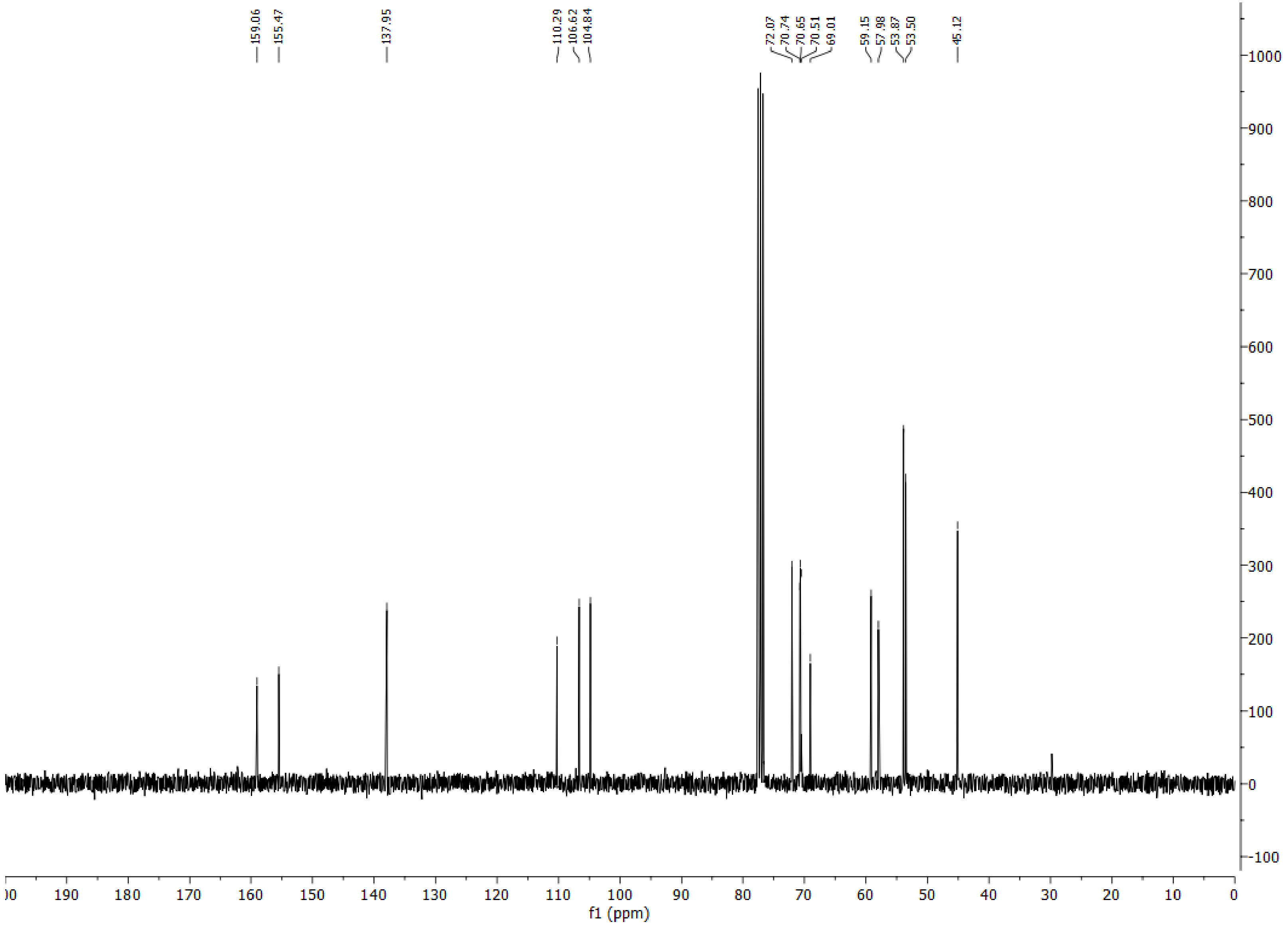

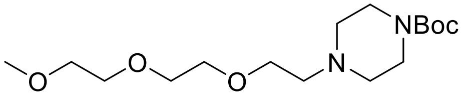

17

^1H NMR (400 MHz, $CDCl_3$)

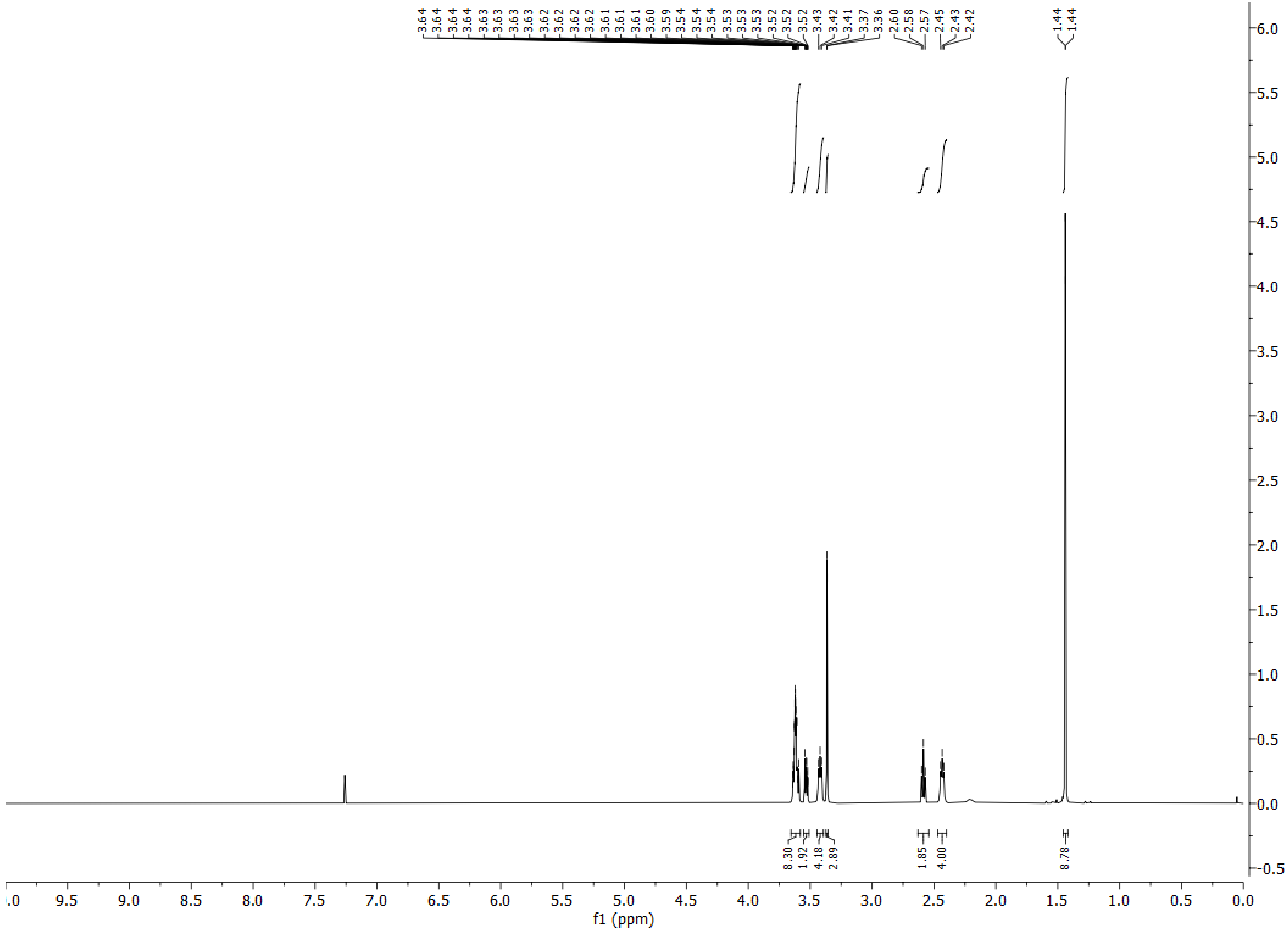

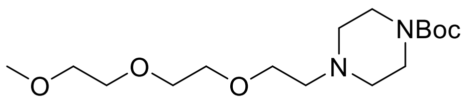

17

^{13}C NMR (101 MHz, $CDCl_3$)

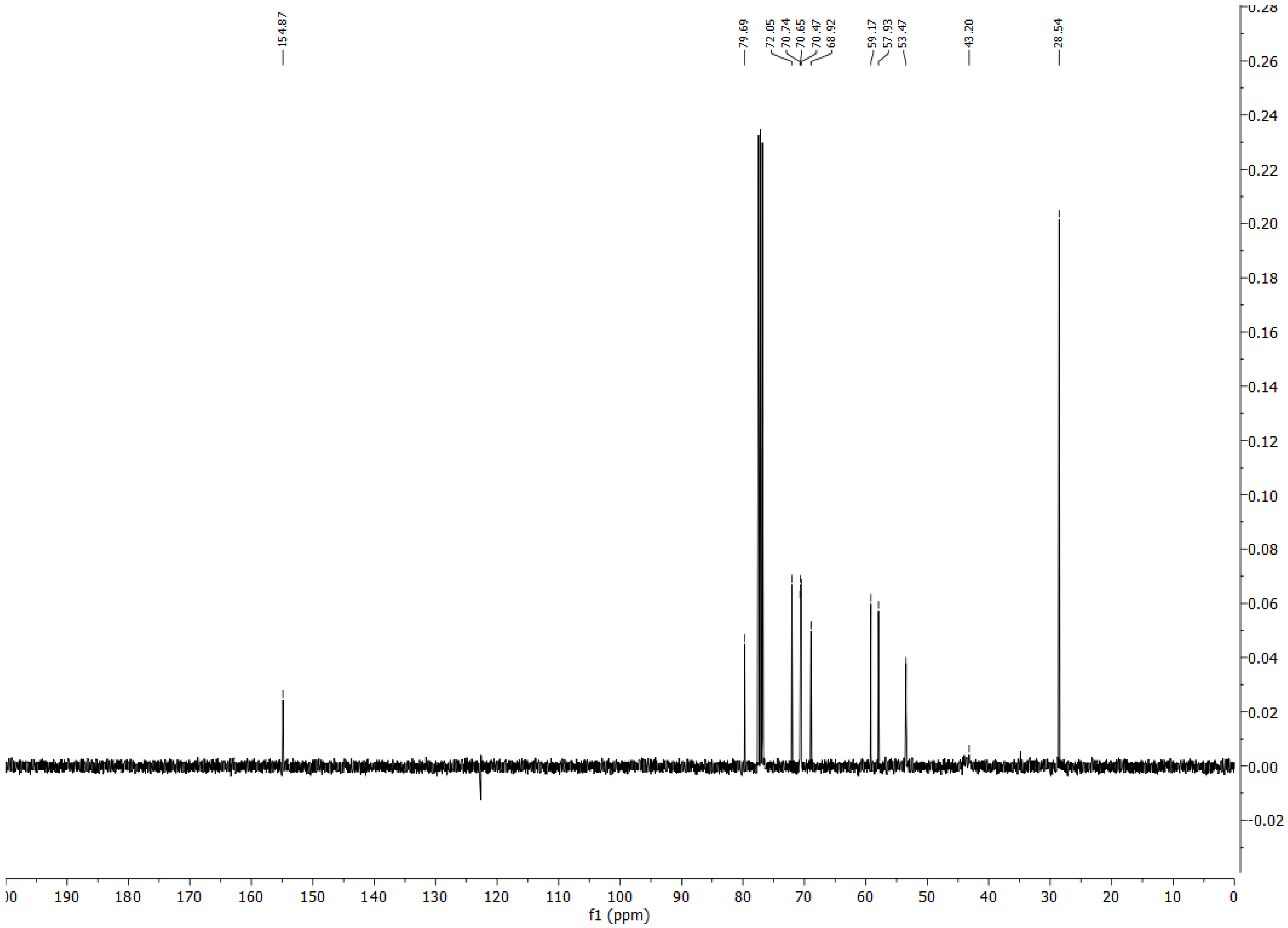

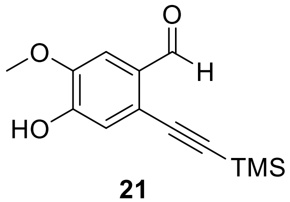

21

^1H NMR (400 MHz, $CDCl_3$)

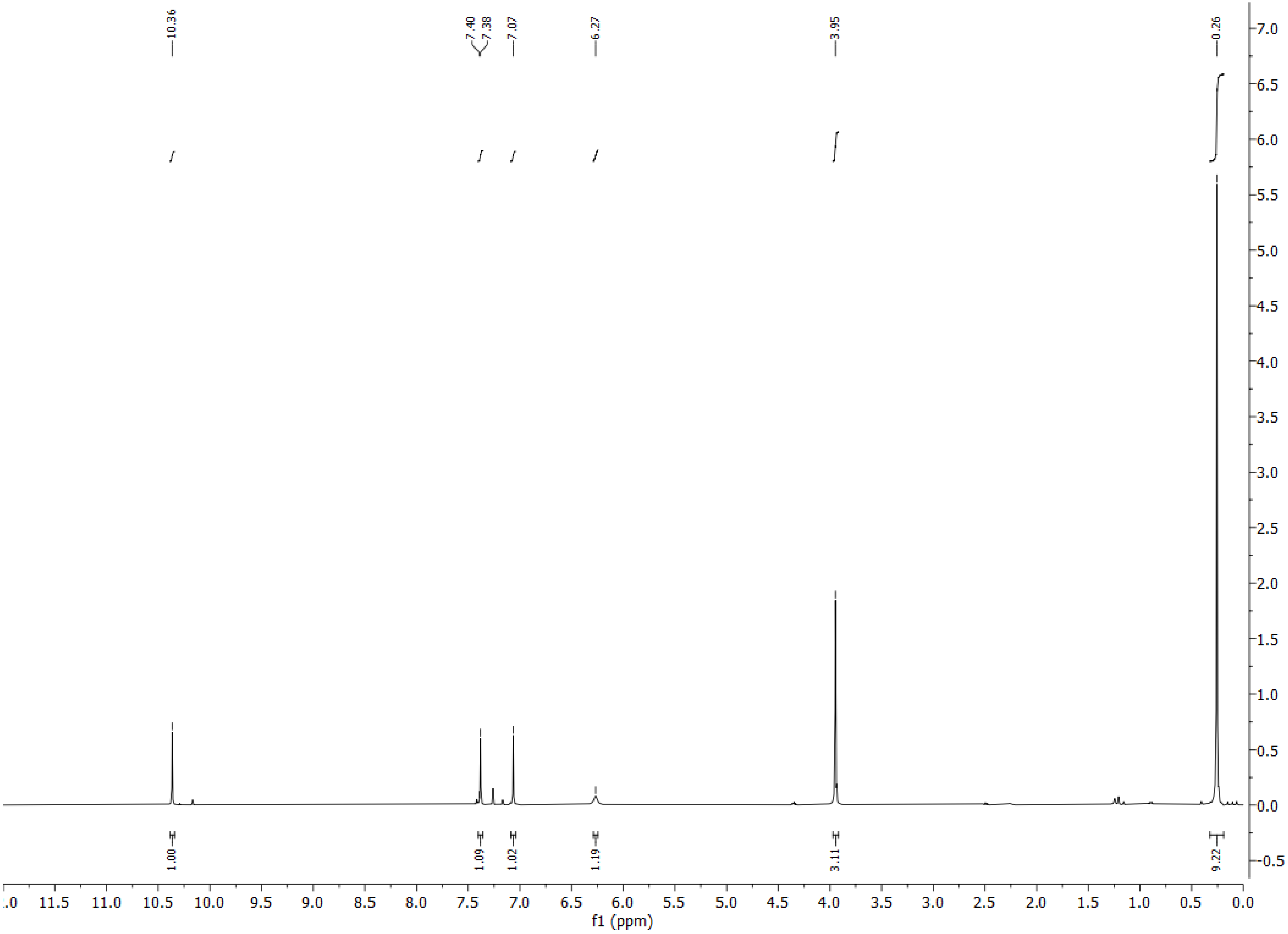

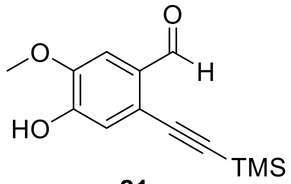

21

^{13}C NMR (101 MHz, $CDCl_3$)

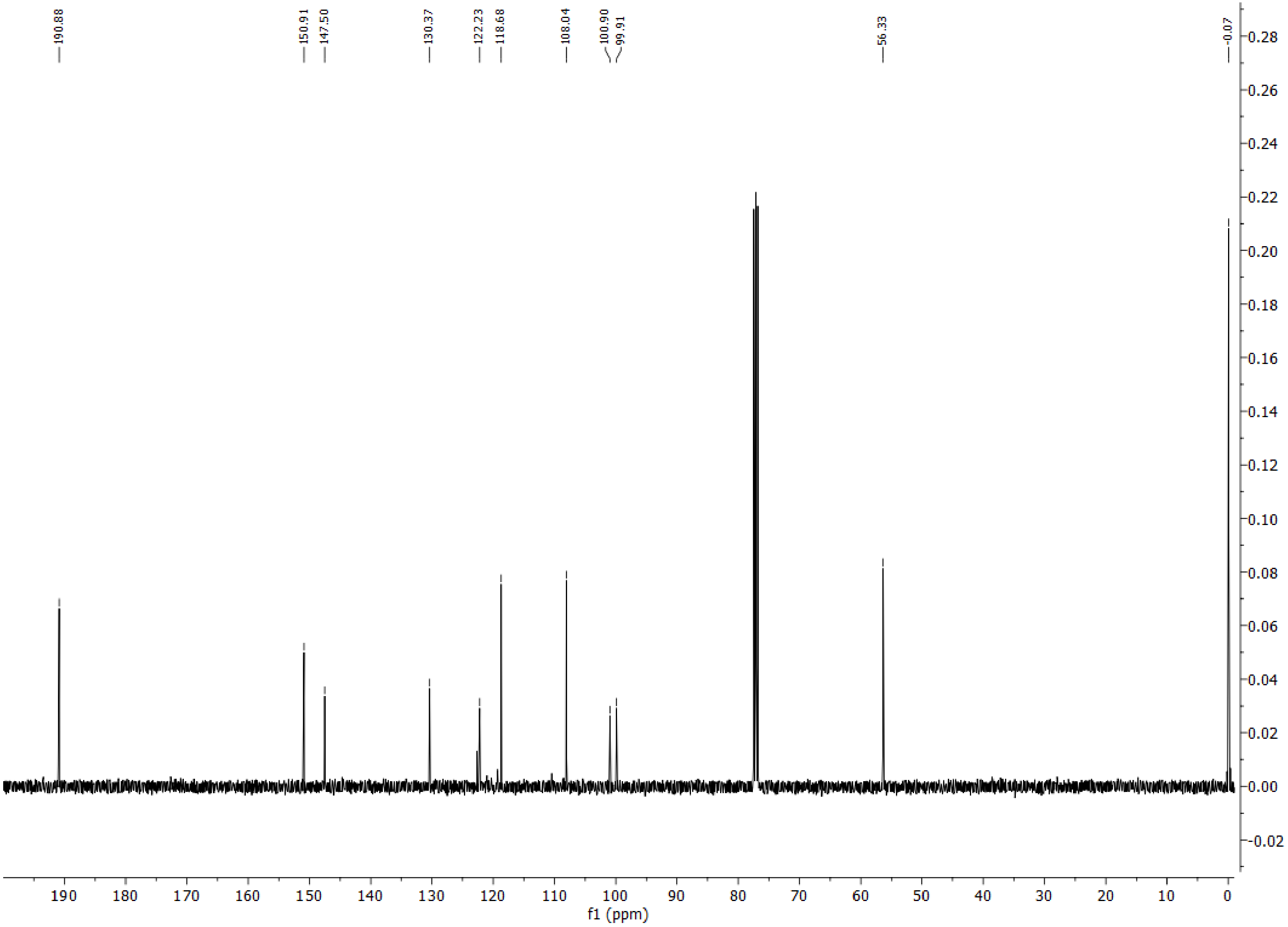

Supplement: CB-004-D2CB00203E-s001 [file CB-004-D2CB00203E-s001.pdf]
